# Evolutionarily Conserved Herpesviral Protein Interaction Networks

**DOI:** 10.1371/journal.ppat.1000570

**Published:** 2009-09-04

**Authors:** Even Fossum, Caroline C. Friedel, Seesandra V. Rajagopala, Björn Titz, Armin Baiker, Tina Schmidt, Theo Kraus, Thorsten Stellberger, Christiane Rutenberg, Silpa Suthram, Sourav Bandyopadhyay, Dietlind Rose, Albrecht von Brunn, Mareike Uhlmann, Christine Zeretzke, Yu-An Dong, Hélène Boulet, Manfred Koegl, Susanne M. Bailer, Ulrich Koszinowski, Trey Ideker, Peter Uetz, Ralf Zimmer, Jürgen Haas

**Affiliations:** 1 Max-von-Pettenkofer Institut, Ludwig-Maximilians-Universität, München, Germany; 2 Division of Pathway Medicine, University of Edinburgh, Edinburgh, United Kingdom; 3 Institut für Informatik, Ludwig-Maximilians-Universität, München, Germany; 4 Institut für Genetik, Forschungszentrum Karlsruhe, Karlsruhe, Germany; 5 J. Craig Venter Institute, Rockville, Maryland, United States of America; 6 Medizinische Biochemie und Molekularbiologie, Universität des Saarlandes, Homburg, Germany; 7 Deutsches Krebsforschungszentrum, Heidelberg, Germany; 8 Program in Bioinformatics, University of California San Diego, La Jolla, United States of America; 9 Department of Bioengineering, University of California San Diego, La Jolla, United States of America; University of California at Los Angeles, United States of America

## Abstract

Herpesviruses constitute a family of large DNA viruses widely spread in vertebrates and causing a variety of different diseases. They possess dsDNA genomes ranging from 120 to 240 kbp encoding between 70 to 170 open reading frames. We previously reported the protein interaction networks of two herpesviruses, varicella-zoster virus (VZV) and Kaposi's sarcoma-associated herpesvirus (KSHV). In this study, we systematically tested three additional herpesvirus species, herpes simplex virus 1 (HSV-1), murine cytomegalovirus and Epstein-Barr virus, for protein interactions in order to be able to perform a comparative analysis of all three herpesvirus subfamilies. We identified 735 interactions by genome-wide yeast-two-hybrid screens (Y2H), and, together with the interactomes of VZV and KSHV, included a total of 1,007 intraviral protein interactions in the analysis. Whereas a large number of interactions have not been reported previously, we were able to identify a core set of highly conserved protein interactions, like the interaction between HSV-1 UL33 with the nuclear egress proteins UL31/UL34. Interactions were conserved between orthologous proteins despite generally low sequence similarity, suggesting that function may be more conserved than sequence. By combining interactomes of different species we were able to systematically address the low coverage of the Y2H system and to extract biologically relevant interactions which were not evident from single species.

## Author Summary

Herpesvirus proteins interact with each other in a complex manner throughout the infectious cycle. This is probably best exemplified in the process where a large number of viral proteins come together to form new viral particles which are subsequently released from the infected cell. A more detailed understanding of how viral proteins interact with each other might assist the development of drugs which may inhibit these interactions and consequently block viral replication. Here we present three genome-wide studies of protein-protein interactions in the herpesviruses herpes simplex virus I, murine cytomegalovirus and Epstein-Barr virus. Altogether we identified 735 interactions in the three viruses, most of which have not previously been reported. By combining these studies with our previously published studies for Kaposi's sarcoma-associated herpesvirus and varicella-zoster virus we were able to perform a comparative analysis of interactions in five related viral species. We observed that a high proportion of interactions were conserved between the different species, despite a low degree of sequence conservation. This implies that by comparing interaction data, we were able to increase the coverage of our viral networks and thus obtain a better and more complete picture of interactions between herpesviral proteins.

## Introduction

**Figure 1:**
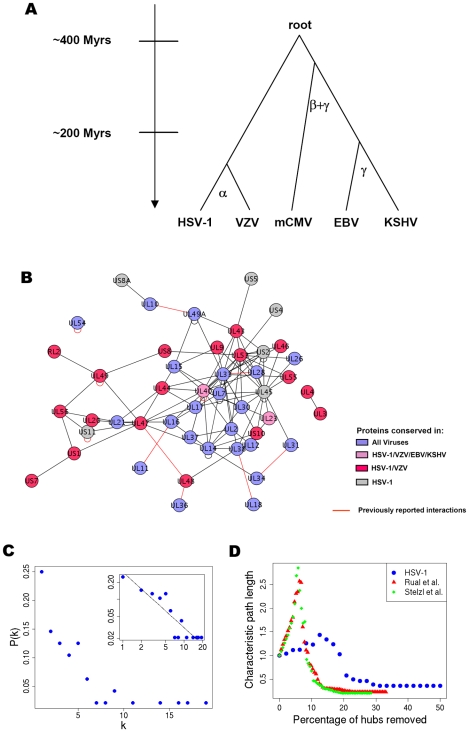
Intraviral protein interactions in HSV-1. (A) Phylogeny of the five investigated herpesviruses and their classification into the three subfamilies α, β, and γ. A timeline is added to indicate approximately when the different subfamilies were separated based on findings by McGeoch and colleagues [6],[7]. (B) Intraviral protein-protein interaction network for HSV-1. The proteins are coloured according to their conservation in the herpesvirus phylogeny: the blue nodes are core proteins conserved in all five viruses, two nodes (pink) are conserved in α and γ herpesviruses, several red ones in α herpesviruses and the grey ones are specific to HSV-1. Edges indicate observed interactions in HSV-1, and red edges indicate previously reported interactions. The protein interaction network was generated using the Cytoscape software (www.cytoscape.org) [58].. (C) node degree distribution on a linear or logarithmic (inset) scale. The herpesviral networks can be approximated by power law distributions (Table S3). (D) Simulations of deliberate attack on HSV-1 in comparison to two human networks by removing their most highly connected nodes in decreasing order. After each node is removed, the new network characteristic path length (average distance between any two nodes) of the remaining network is plotted as a multiple or fraction of the original parameters. The herpesviral networks consistently exhibited a higher attack tolerance, as the increase in path length is considerably smaller.

**Figure 2:**
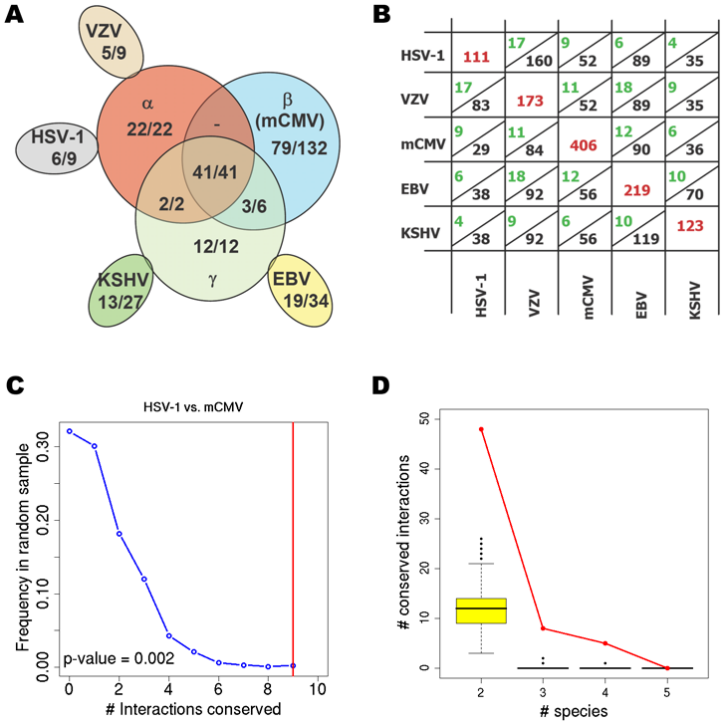
Overlap of herpesviral protein-protein interaction networks. (A) shows the sunflower structure induced by the protein sets of the five viruses and their intersections. For each overlapping area the number of shared proteins with detected interactions, in addition to the total number of shared proteins are indicated. All shared proteins in the various subgroups are interacting (with the exception of the β+γ subgroup where only three out of six shared proteins are interacting) while for the individual viruses between 50% and 70% of the proteins have observed interactions. (B) Comparison of conserved interactions between orthologous proteins for any two herpesvirus species. In each rectangle, the value above the lines indicates the observed number of homologous interactions detected in both herpesviruses (in green). The value below the line (in black) gives the total number of interactions detected in the first species (indicated in columns) between proteins which have orthologs in the second species (indicated in rows). On the diagonal, the total number of interactions is shown for each virus. (C) Distribution of the number of conserved interactions between HSV-1 and mCMV for 1000 random orthology assignments (blue line) in comparison to the true number of conserved interactions (red vertical line). For each pairwise comparison, subnetworks were selected between proteins conserved in both viruses and then the orthology assignments between the proteins were randomized. Accordingly, the size and degree distribution of the subnetworks does not change. (D) Comparison of the number of interactions conserved in 2, 3, 4 and 5 species for 1000 random orthology assignments (yellow boxes) to the true number of interactions conserved in that many viruses (red line). Random orthology assignments were created in a similar way as for Figure 2C.

Herpesviruses are subdivided into three taxonomic subfamilies (α, β and γ) based on both genomic composition and biology according to a well-known phylogeny [1],[2],[3] (Figure 1A). While all herpesviruses are structurally similar, the different subfamilies are highly divergent in genome size, content and organization. The genome size ranges from 120 kbp for varicella-zoster virus (VZV), which belongs to the α-herpesviruses, to 240 kbp for human cytomegalovirus (hCMV), a member of the β-herpesviruses [4],[5]. Gene-coding potential is reflected in the size of the genomes with VZV containing 70 open reading frames (ORFs) and hCMV containing ∼170 ORFs. The overlap between the protein sets of the five viruses clearly supports the known phylogeny, but there are also some proteins shared among viruses not consistent with the phylogeny (Figure 2A). Although the three subfamilies are thought to have diverged from a common ancestor around 400 million years ago (McGeoch 2006), they still contain a set of 41 core orthologs present in all herpesviruses [6],[7]. Herpesviral core proteins are generally involved in fundamental aspects of viral morphogenesis (e.g. DNA replication, DNA packaging, structure and egress), and are consequently often essential for replication in cell culture [8],[9],[10].

Several genome-wide yeast-two-hybrid (Y2H) studies of protein-protein interactions in eukaryotes have been published over the last years, including *Saccharomyces cerevisiae* [11], *Caenorhabditis elegans* [12], *Drosophila melanogaster* [13], *Plasmodium falciparum* [14] and *Homo sapiens* [15],[16]. The first complete genome-wide interaction study, however, was published for the *E.coli* phage T7 [17]. With their relatively small genomes and few genes, viruses seem the ideal candidates for studying protein-protein interactions on a genome-wide level and to address the generally low coverage of Y2H measurements in a systematic way. It is therefore surprising that not more genome-wide studies of intraviral interactions have been performed to date. With the exception of bacteriophage T7 [17] and Vaccinia virus [18], most of the studies of viral interactions have been performed on small RNA viruses [19],[20],[21],[22]. Recently more studies have been focusing on larger DNA viruses. In addition to our previous studies of VZV and Kaposi's sarcoma-associated herpesvirus (KSHV) [23], a study by Calderwood and colleagues identified 43 interactions between viral proteins in Epstein-Barr virus (EBV) [24]. Two Y2H studies on herpes simplex virus 1 (HSV-1) and KSHV have also focused on interactions between structural components of these viruses [25],[26]. To add to our understanding of intraviral interactions in herpesviruses we present in this article the first interactomes for herpes simplex virus I and murine cytomegalovirus (mCMV), in addition to a second and independent interactome for Epstein-Barr virus. Based on these data we are able to compare five related interactomes, obtained using a standardized experimental setup for all five species. From the comparison and extensive experimental testing by CoIP we conclude that i) genome-wide interaction studies are sufficiently sensitive for between-species comparisons to identify the basic sunflower structure of the interaction networks and their common core, ii) interactions are to a large degree conserved between orthologs in herpesviruses, iii) comparing interactomes from several species can improve the low coverage of individual Y2H measurements and iv) biologically relevant interactions which may not be apparent from the interactome of a single species, often become obvious when multiple interactomes are aligned and compared.

## Results

### Comparison of herpesviral interactomes

To study intraviral protein-protein interactions of herpesviruses we recombinatorially cloned the individual open reading frames of HSV-1, mCMV and EBV into the yeast-two-hybrid (Y2H) vectors pGBKT7-DEST and pGADT7-DEST and tested all pairwise intraviral protein interactions using an array-based Y2H strategy [27]. To address the issue of false negative interactions, viral proteins containing transmembrane domains were cloned both as full-length and as intracellular and/or extracellular domains. From the mCMV Y2H analysis we observed that 33% of the tested preys, and 40% of the baits, gave positive interactions. Similar results were observed with HSV-1 and EBV with ∼1/3 of the clones yielding positive interactions (Table S1). In total, the Y2H analysis revealed 111 interactions for HSV-1, 406 for mCMV and 218 for EBV (Figures 1B, S1 and S2, Tables S2 and S13). Combined with our previously published interactomes for VZV (173 interactions) and KSHV (123 interactions), we obtained altogether 1,031 intraviral interactions in five herpesviral species (Tables S2 and S13). To evaluate the coverage of our five interactomes we performed an extensive literature search which identified 257 previously published interactions for these herpesviruses (including human cytomegalovirus (hCMV) homologues). Of these 257 interactions we were able to detect 24 (9.3%) in at least one virus (Figures S3 and S4 and Tables S3 and S14). When comparing our EBV interactome with the recently published EBV network by Calderwood et al., 6 out of 43 (13.9%) interactions could be confirmed [24]. Such low confirmation rates are common to Y2H studies, even for studies within the same species, which in general suffer from low coverage [28],[29],[30]. For instance, in a previous study of human interactions only 2.3–8.4% of known interactions were identified [15]. On the other hand, this implies that ∼3% of the interactions found in the present study have been published so far in the literature or identified in previous genome-wide screens in the case of EBV. In the case of HSV-1 our study added 102 new interactions to the network of already known interactions (coloured grey in Figure 1B). As is typical for such interaction networks, no apparent structure can easily be recognized.

A comparison of the five herpesviral networks revealed that the degree distribution differed from cellular networks, local clustering was not as high as expected in *small-world* networks of this size (Figures 1C and S5 and Table S4), and attack tolerance and robustness were increased compared to cellular networks (Figure 1D and S6), probably reflecting that the viral interactome in itself only represents a minor part of the complete interactome of the infected cell. In a previous study we observed that the topology of the KSHV and VZV networks approached that of cellular networks as the viral interactomes were connected into a human interactome [23]. The observations presented here confirm our previous findings, and indicate that herpesviral PPI networks share an evolutionarily conserved topology.

Apart from general topological features, herpesviral interactomes were also compared on the level of individual interactions. For this purpose, we used the orthology assignments based on sequence similarity and gene order (Table S5) [31]. Species within the same subfamily are generally characterized by higher sequence similarity between orthologous proteins. They also share more orthologous proteins with each other than species from different subfamilies (Figure 2A). In previous inter-species comparisons [32], very few interactions were found to be shared between different species (yeast, worm, fly). Unlike the previous comparative studies, the five different interactomes analysed in this study were obtained using exactly the same experimental protocols. Nevertheless, we still observed little overlap between the networks of the five herpesviruses. Of 488 (409 non-redundant, i.e. conserved interactions are only counted once) interactions between proteins conserved in more than one species, 140 (61 non-redundant) (28.7% or 14.9% non-redundant, respectively) interactions were conserved between at least two species. For any two herpesvirus species, we compared the number of interactions between proteins conserved in both species against the number of interactions found in both species (Figure 2B). Although the pair wise overlaps observed were small, they were nevertheless significantly higher than observed with randomized orthology assignments (Figures 2C and S7). Randomized orthology assignments for each pair of herpesviruses were obtained by first selecting the sub-network of conserved proteins between the two species, and then randomizing the orthology assignments for these sub-networks.

A similar analysis was performed for all five networks taken together. First, networks were divided into interactions conserved within a subfamily or between different subfamilies, and the number of interactions conserved in 2, 3 or 4 species in each category was evaluated. We furthermore compared the number of interactions conserved in 2, 3, 4 and 5 species against the results for randomized orthology assignments and found in each case a significant enrichment (Figure 2D). This shows that despite the low coverage of the Y2H system significant conservation can still be observed.

### Interactions among core proteins are conserved

**Figure 3:**
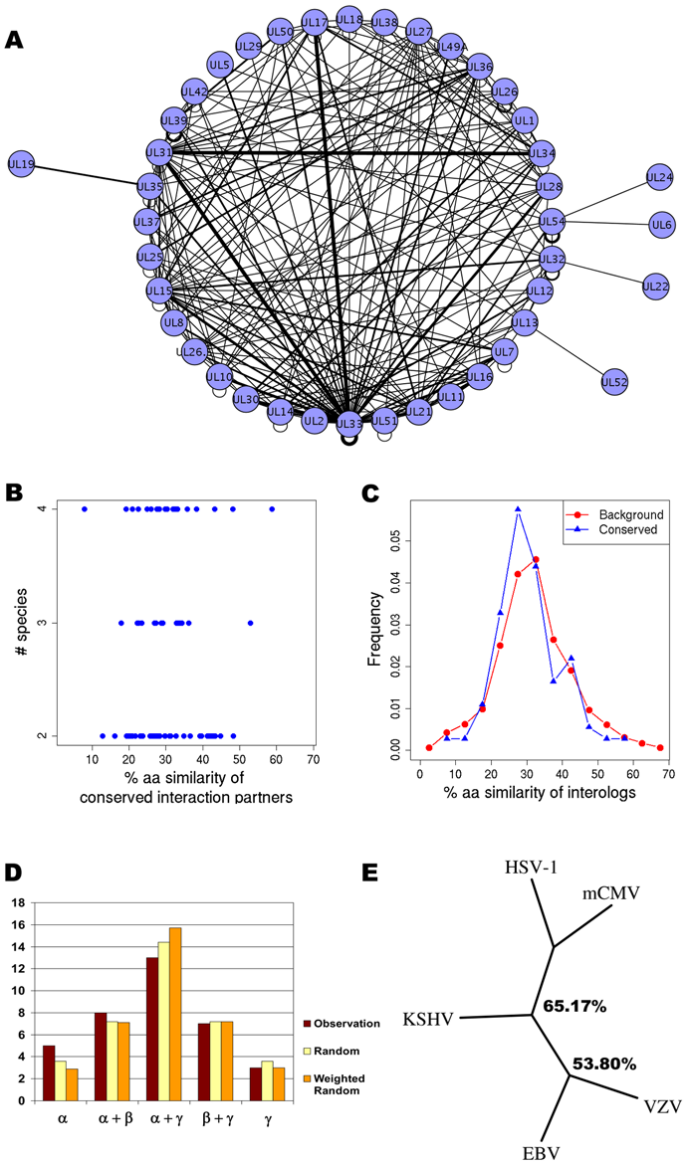
The core network in herpesviruses. (A) Core interaction network between the 41 core orthologous proteins. The width of the edges indicates the number of species in which the interaction was detected. Proteins are labelled with the HSV-1 protein names. Networks were illustrated using Cytoscape [58]. (B) The left plot of the figure shows the number of species for which an interaction is observed (y-axis), plotted against the sequence similarity of the two interacting proteins (x-axis). No correlation between sequence similarity and number of conserved interactions was detected. This is further quantified in (C) showing the distribution of similarities of the interacting proteins as compared to the background distribution of similarities derived from sequence similarities of protein pairs not interacting. (D) Compares the distribution of interactions conserved in two species within or across subfamilies against the random expectation if all possible combinations are equally likely or weighted based on the number of interactions in the core of each species. Interactions are not conserved preferentially between closely related species and no significant difference to the random expectation can be observed. (E) Phylogenetic tree for the herpesviral core network. Bootstrap values are indicated on the internal nodes.

**Figure 4:**
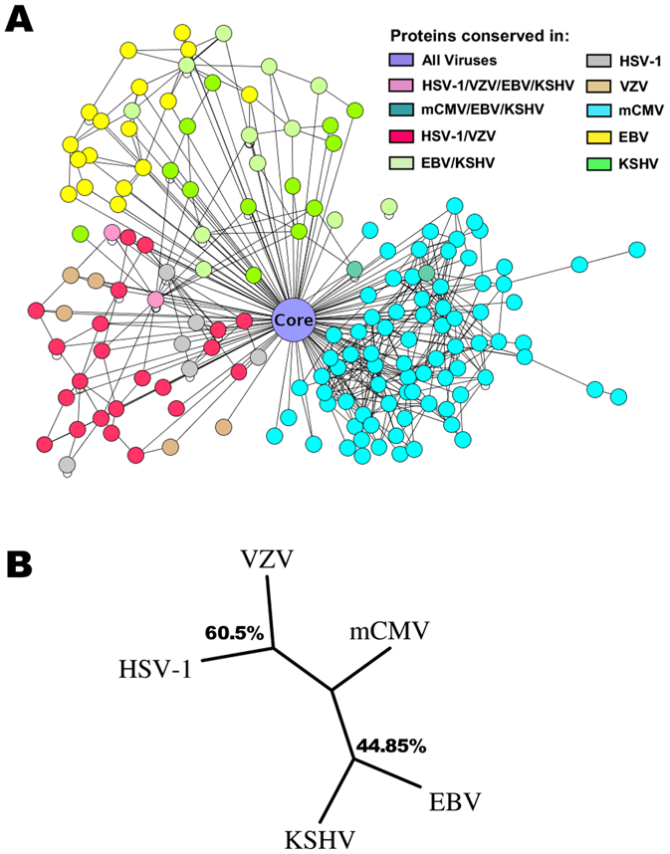
The non-core network in herpesviruses. (A) Interaction networks of the non-core proteins of the five herpesviruses. All core proteins and the interactions between them are reduced to one central node (the core) surrounded by the leaves formed by the subgroup and individual interaction networks. The colour code is the same as in Figure 1B. (B) Phylogenetic tree based on the complete herpesvirus protein networks. Bootstrap values are indicated on the internal nodes.

Herpesviruses share a set of 41 core orthologous proteins which are conserved throughout the three subfamilies (Table S5) [31]. These core orthologs comprise approximately half of the genome of HSV-1, VZV, EBV and KSHV but less than 25% of mCMV. They can be further subdivided into a group of 31 orthologs with relatively high sequence similarity (approximately 30–60% sequence similarity), and a group of 10 orthologs with little similarity (approximately 16–30% similarity) (Table S6). Based on this orthology assignment, we generated an overlay of all protein interactions between the core orthologs detected in any of the five herpesviruses (Core network, Figure 3A). Of a total of 283 (218 non-redundant) core protein interactions detected, 113 (48 non-redundant, 39.9%) were found in more than one species (Table S7). For the core network, we did not observe a correlation between sequence similarity and the number of conserved interactions detected (Figures 3B and 3C). For example, the interaction between the two tegument proteins UL11 and UL16 in HSV-1 was also detected in mCMV and EBV, although sequence similarity of UL11 and its orthologs across subfamilies is quite low (28%). This interaction was interestingly also observed for HSV-1 in a recent report by Vittone and colleagues [33]. In addition, interactions were *not* preferentially conserved between closely related species (Figures 3D and S8). Accordingly, overlaps between the interaction sets in the core network were not correlated to the true phylogeny of herpesviruses (Figure 3E). Indeed, the highest overlap was observed between HSV-1 (α-subfamily) and mCMV (β) which belong to lineages separated early in herpesvirus evolution [7]. However, since our phylogenetic trees are based on relative overlaps between the different species, we cannot exclude that a more complete set of core interactions might have allowed for better separation of the subfamilies. In contrast, when also including subfamily- and species specific interactions (i.e. the complete interaction network of the five herpesviruses with the characteristic sunflower structure, see Figure 4A), the analysis yielded a phylogeny that was consistent with the known evolutionary relationships (Figure 4B). This indicates that the presence of conserved subfamily specific interactions provides sufficient conserved and non-conserved interactions to accurately separate the subfamilies from each other.

In the overlay of all five herpesviral networks (Figure 4A, sunflower structure), the core network is indicated as a central node common to all herpesviruses. Subfamily- and species-specific networks are attached (as leafs) to this core. Only few connections exist between the subfamily-specific networks due to few shared proteins outside of the core. Our data provides evidence that the viral core network is extremely dense while the non-core network appears relatively sparse. However, since non-core interactions were tested in at most two species, and not in five as the core interactions, the non-core network may be equally dense. Indeed, no consistent difference was observed between the number of intraviral core and non-core interactions when considering each network separately (Table S8).

**Figure 5:**
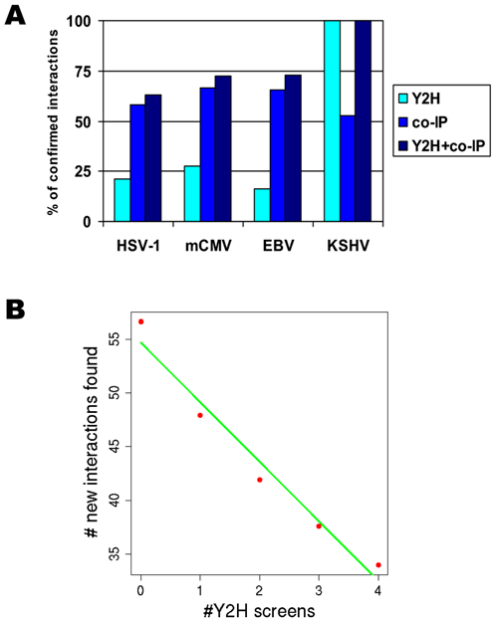
Core interactions are conserved. (A) Histogram indicating the percentage of interactions confirmed either by Y2H, CoIP or Y2H and CoIP. We tested in total 92 interactions in HSV-1, mCMV and EBV predicted from 55 interactions detected in KSHV between proteins conserved in at least one other species. For all species more than 50% of the predicted interactions could be confirmed by CoIP, while the numbers confirmed by Y2H were significantly lower. (B) Illustrates how the discovery of new interactions decreases for each new interactome. Average number of new interactions found after 1, 2, 3, 4 and 5 Y2H screens (red points) is plotted, where averages are taken over all possible sequences of Y2H screens. The green line shows a linear best fit line. While there is a clear decrease in the number of new interactions discovered for each additional Y2H analysis, there seems to be a significant number of interactions still to be found.

To further evaluate whether interactions between orthologous proteins are conserved we used co-immunoprecipitation to test 92 interactions predicted from 55 interactions detected in KSHV for the corresponding orthologs in HSV-1, mCMV and EBV. 11/19 (58%) of the predicted interactions could be confirmed by CoIP in HSV-1, 12/18 (67%) in mCMV and 36/55 (65%) in EBV, in comparison to 29/55 (53%) in KSHV itself (Figures 5A and S9A and Table S9). The percentage of core-derived orthologs that were confirmed by CoIP significantly correlated with the number of species in which the interactions were detected in Y2H screens, suggesting that the accuracy increases with the number of positive assays (Figure S9B, Table S10). As negative controls, ten interactions which were not detected in any of the Y2H screens were tested in four viruses (39 interactions in total, Table S10). Although the confirmation rate of these negative controls seems relatively high (6/39 (15%)), it is still significantly smaller than for the predicted interactions and correlates well with the confirmation rates of interactions observed in 2, 3 and 4 species (Figure S9B). Due to the low coverage of the Y2H system many (particular weak) interactions were most likely missed, and the positively tested controls may be examples of such interactions. It also suggests that, although our core interactome is very dense, it has not yet reached full coverage. Since the confirmation rate by CoIP for the Y2H interactions in KSHV is not higher than for the predicted interactions in HSV-1, mCMV and EBV, we conclude that a high percentage of interactions between core orthologs are conserved despite low sequence similarity of some of the orthologs across subfamilies.

To further assess the level of completeness of our core network, we evaluated the average number of new interactions added to the core network with each new Y2H screen (Figure 5B). If core interactions indeed are conserved, as indicated by our predicted interactions, we would expect the coverage to increase with each new herpesviral interactome. Although the number of newly discovered interactions steadily decreased with each new screen, saturation does not seem to be reached yet. Thus, although coverage for the core network could be increased, a significant fraction of interactions might still be missing.

Finally, to determine if conserved intra-viral interactions allow viral proteins to interact across different species, we tested four interactions which were detected in at least two herpesviruses in the original screens by Y2H and LUMIER (luminescence-based mammalian interactome mapping) pull-down assays (Figure S10A and B) [34]. While Y2H in general yielded few cross-species interactions, we detected a larger number of interactions by LUMIER (Figure S10B). The cross-species interactions between the HSV-1 UL11 and UL16 tegument and between the HSV-1 UL19 and UL35 capsid orthologs were mainly observed within a specific subfamily, in accordance with previous observations by Schnee et al. [35]. For the two other interactions, involving orthologs with both a high and low degree of sequence similarity based on Table S6, we saw a more promiscuous interaction pattern. HSV1 UL14 for example was able to interact with HSV-1 UL33 and its orthologs in all five species, suggesting that sequence similarity might be a poor predictor of interspecies interactions in herpesviruses. Additionally, we tested 4 core and 4 noncore VZV baits against prey libraries of all five viruses. As expected, the intra-species analysis (VZV baits against VZV preys) yielded the highest fraction of positive interactions (2.8%), compared to 0.5% positive interactions in the cross-species screens. Of the positive cross-species interactions we observed 4 core-core, 15 core-noncore and 2 noncore-noncore interactions (Table S11). When the number of positive interactions was correlated to the number of interactions tested for each class, we observed a significant enrichment of positive interactions for the core-core and core-noncore classes compared to the noncore-noncore class (Figure S11).

### M51 interacts with the nuclear egress complex

**Figure 6:**
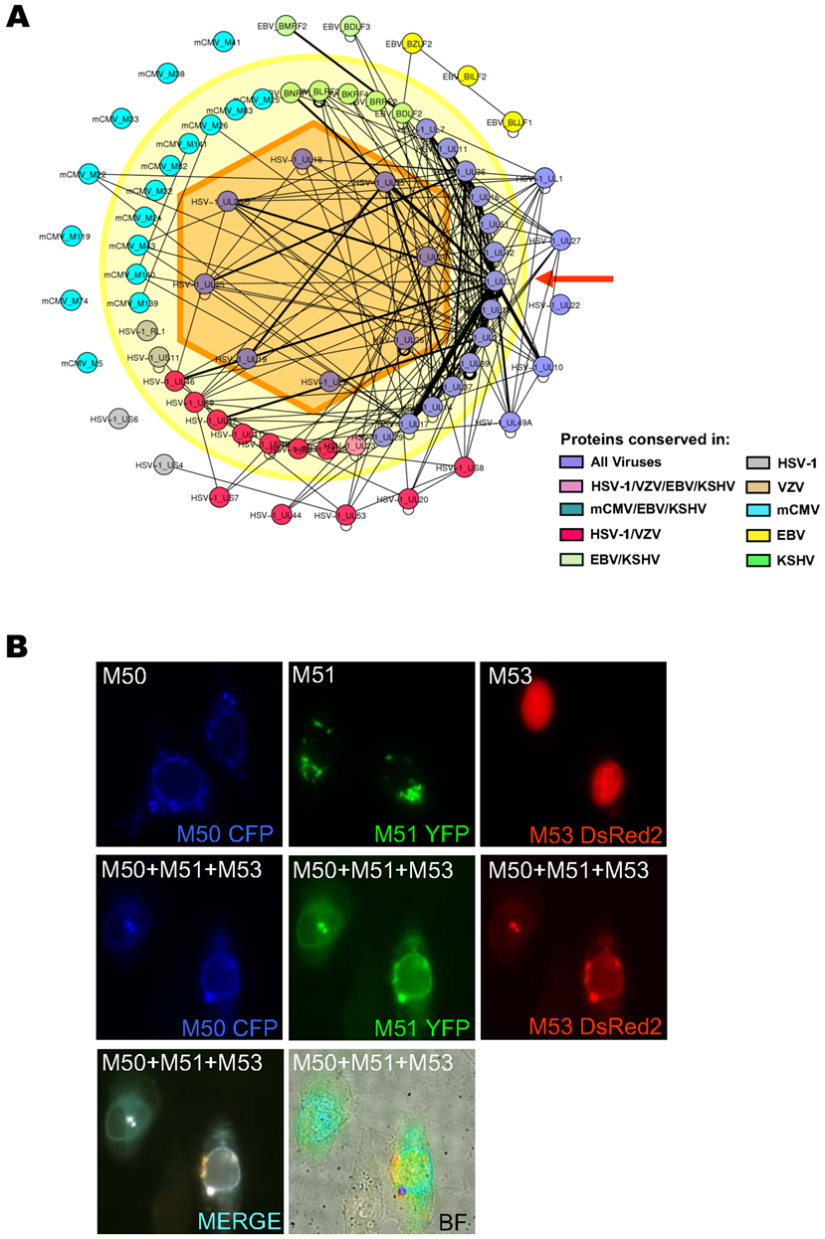
High-confidence protein interactions in herpesvirus virions. (A) Schematic map of protein interactions within herpesvirus virions. The viral proteins indicated (or their orthologs) have been shown to be present in virus particles by proteomic analyses [59],[60]. The central nodes indicate capsid proteins, the middle layer tegument proteins and the outer layer glycoproteins. The colour code is the same as in Figure 1B. Indicated are all interactions detected in one of the five species and the width of the edges indicates the number of species in which the interactions were detected. M51 is marked by the red arrow. (B) mCMV M50, M51 and M53 co-localize at the nuclear membrane. CFP-tagged M50, YFP-tagged M51 and DsRed2-tagged M53 were expressed either alone (upper row) or together (lower 2 rows) and analysed by fluorescence or bright field (BF) microscopy.

Most core proteins are essential, and a majority can be found in herpesvirus virions composed of an icosahedral capsid of 162 capsomers, an amorphous tegument layer and a lipid bilayer membrane with embedded glycoproteins. Using the high-coverage core network, a map of conserved protein interactions in herpesviral particles was generated (Figure 6A and S12). One outstanding example for a highly connected protein in this virion map is the mCMV M51 ortholog (HSV-1 UL33, VZV Orf25, EBV BFRF4 and KSHV Orf67.5), which interacted with 14 tegument proteins. Since (i) 11 of the 14 interactions (79%) of this protein were found in more than 1 species, (ii) most Y2H interactions were confirmed even under high concentrations of the competitive HIS3 inhibitor 3-amino-1,2,4-triazole which can be used to suppress non-specific Y2H interactions (Figure S13A and S13B), and (iii) a majority of interactions were confirmed by CoIP (Table S12), we considered them as high-confidence interactions. Furthermore, 4 of the 5 interactions conserved in 4 herpesviral species are M51 interactions. One example is the interaction of mCMV M51 orthologs (HSV-1_UL33/VZV_25/EBV_BFRF4/KSHV_67.5) with M53 orthologs (HSV-1_UL31/VZV_27/EBV_BFLF2/KSHV_69), which also interact in 4 species with M50 orthologs (HSV-1_UL34/VZV_24/EBV_BFRF1/KSHV_67). M50 and M53 and their orthologs are involved in the nuclear egress of viral capsids and are well-characterised in mCMV, HSV-1, EBV and pseudorabiesvirus [36],[37],[38],[39]. Both M50 in mCMV and its ortholog UL34 in HSV-1 recruit protein kinase C to the nuclear membrane, which subsequently phosphorylates lamins to dissolve the nuclear lamina allowing the capsids to reach the inner nuclear envelope [38],[40]. In mCMV, we confirmed 17/22 (>75%) (Table S12) of M51 interactions by CoIP, and showed that M51 is targeted to the nuclear membrane by M50 and co-localizes with both M50 and M53 (Figure 6B). Our results suggest that M51 and its orthologs are part of a larger protein complex and may be involved in nuclear egress. Since most of its interaction partners are present in the virion tegument we hypothesize that it plays a role in tegument formation, and represents a possible link between DNA packaging, nuclear egress and tegumentation.

## Discussion

Here we present an extensive study of intraviral protein-protein interactions for the three herpesviruses HSV-1, mCMV and EBV, using Y2H as the main experimental method. By combining the results with our previous studies of interactions in VZV and KSHV we were able to compare the interactomes of five related herpesviral species. Although there was little overlap between the five viral networks according to the Y2H maps, we were able to show that interactions between core orthologous proteins are to a large degree conserved between species of different subfamilies. By generating a separate network of interactions between core proteins of five herpesviruses, we were also able to overcome the coverage problem of Y2H and to identify interactions of interest from the common network which were not apparent in each single network.

While the overlaps between the different interactomes were generally quite low, there was still a significant enrichment of conserved interactions between orthologous proteins for any pair of the five species (Figures 2C and S7A to I). The same holds true for the conservation between all five viruses. Interactions observed in two, three or four species were all enriched significantly as compared to background expectations (Figure 2D). This argues that, although troubled with false negative and false positive interactions, Y2H as a technique is still sufficiently sensitive and specific to obtain data for a comparative analysis of related interactomes. Similar observations were also made for the recently published *Campylobacter jejuni* interaction network, where highly significant overlaps were found with both the *Escherichia coli* and *Helicobacter pylori* interactomes [41]. It is very likely that true overlaps between the herpesvirus interactomes are higher, but that due to false negative interactions we only observe modest overlaps. There are numerous reasons why interactions may be missed in the Y2H system, including improper folding of fusion proteins and post-translational modifications. In an attempt to address some of these issues we cloned all the viral proteins containing transmembrane domains as both full length and extra/intracellular fragments, which has been reported to increase sensitivity [42].

Our observations indicate that intraviral interactions between core proteins are conserved, and as a result we are *not* able to separate the Y2H interactomes into their phylogenetic subfamilies solely based on their core interactions. However, when interactions involving subfamily-specific proteins present in at least two of the virus species were included, we were able to generate a correct phylogenetic tree. This implies that interactions involving subfamily-specific proteins are at least partly conserved. Indeed, several of the interactions predicted from KSHV and confirmed in EBV by CoIP involved subfamily specific proteins. From published literature there are several examples of core interactions being conserved between species of different herpesviral subfamilies, e.g. the interactions between HSV-1 UL31 and UL34 [36],[37],[38],[39], HSV-1 UL15 and UL28 [43],[44] and the HSV-1 UL54 self-interaction [45],[46]. Indeed, much of what is currently known about herpesvirus biology is derived from studies of Herpes Simplex Virus and extrapolated to other species. Our study indicates that it is effectively possible to transfer intraviral interactions between orthologous proteins from one species to another. Thus, by generating an overlay network from several genome-wide Y2H screens in related species, the large number of false negative interactions within each individual analysis can be overcome and a more complete picture of the core interaction network obtained.

In general, interactions are transferred between different species based on the sequence similarity between the corresponding proteins. In addition, one might expect interactions among orthologous proteins with high sequence similarity to have a higher likelihood of being conserved. Yu and colleagues found that interactions could be confidently transferred from one species to another if the joint sequence identity of the interacting orthologs was >80% [47]. However, since none of the herpesviral core proteins shares such a high degree of sequence similarity across subfamilies, these criteria cannot be applied to herpesviruses. Furthermore, no correlation was observed between sequence similarity and the number of species in which an interaction was observed in the Y2H experiments. Thus, our results show that sequence similarity alone seems to be insufficient for predicting herpesviral interactions from one species onto another.

Our analysis of cross-species interactions indicates an enrichment of interactions involving core proteins (either core-core or core-noncore). The detailed cross-species analysis of the interaction between the major capsid protein (MCP) and the smallest capsid protein (SCP) (HSV-1 UL19-UL35, Figure S10B) only yielded 1 intraspecies interaction by Y2H, however 4 by LUMIER, indicating that this interaction is conserved despite being observed in only one species by Y2H. While capsid proteins and interactions are thought to be highly conserved, most of them were indeed only observed in one species in our genome-wide Y2H screens. However, three of the four observed capsid interactions (HSV-1 UL19-UL35, UL18-UL38, UL18-18 and UL35-UL35) have been published previously (Tables S3 and S14), and, in addition, the LUMIER analysis resulted in an increased number of cross-species interactions. The cross-species interactions between the two tegument proteins HSV-1 UL11 and UL16 (and their orthologs), as well as between the two capsid proteins HSV-1 UL19 and UL35 (and their orthologs), were mainly observed between species within the same herpesviral subfamily. A similar observation has recently been reported by Schnee and colleagues [35], and may indicate that some binding sites are more conserved within herpesvirus subfamilies. The other two interactions could be detected in a larger number of cross-species interactions by both Y2H and LUMIER. HSV-1 UL14, for example, was observed to interact with all orthologs of HSV-1 UL33 by LUMIER. Previous reports suggested that HSV-2 UL14 shares certain similarities with cellular chaperones which may account for its promiscuous binding pattern [48].

In the core network derived from the overlap of all five herpesviruses, mCMV M51, and its orthologs HSV-1 UL33, VZV ORF 25, EBV BFRF4 and KSHV ORF 67.5, show up as intraviral hubs with a number of conserved interactions. For instance, the interaction between M51 and M53 was observed in all species apart from HSV-1. Interestingly, when retesting UL33 interactions under more stringent conditions (Figure S12), the corresponding interaction between UL33 and the M53 ortholog in HSV-1, UL31, is clearly one of the positive interactions on both 2.5 and 5 mM 3AT. These interactions were not included in the HSV interactome to prevent an overrepresentation of interactions tested more than once. While not much is known about M51, M53 has been extensively documented to be involved in nuclear egress through its binding to M50 [35],[38],[49],[50]. The interaction between M53 and M50 was confirmed in this study in four viruses. In addition, from our study of interactions in VZV we observed an association between the ortholog of M51 (ORF25) with the M50 ortholog (ORF24) [23], and retesting of HSV-1 UL33 interactions also revealed its binding to HSV-1 UL34 lacking the transmembrane region (Figure S12). Finally, immunofluorescense studies indicated that M51 co-localizes with both M53 and M50 when using fluorescent fusion proteins. These results suggest a possible role for M51 in nuclear egress through its interactions with M53 and/or M50. As interactions between orthologs of M51 and M53 were observed in members of all three subfamilies, it is likely that this represents a conserved function of M51. Previous studies have indicated that the M51 ortholog in HSV-1 (UL33) is involved in packaging of DNA [51], and that it interacts with at least one of the subunits of the terminase complex (UL28) [52]. In the data presented here, UL33 was observed to interact with UL15 and UL28 in three different species. These results suggest that UL33 represents an association between packaging and egress. Studies done in HSV-1 have indicated that UL33 is associated with the external surface of capsids [53], which would make such a dual role reasonable. While it is not known exactly how UL33 associates with the capsid, the interaction observed between M51 and the smallest capsid protein (m48.2) in mCMV and EBV suggests a possible manner of association.

In summary, this study suggests that a distinctive network topology is still present in all vertebrate herpesvirus species although herpesviruses co-evolved with their hosts for millions of years. Moreover, it provides evidence (i) that interactions and hence functions of proteins may be more conserved than their sequence and (ii) that a common core of protein interactions is conserved in all herpesviruses. We hope that the data presented will inspire future herpesvirus research and facilitate the selection of potential targets for antiviral therapy [54].

## Materials and Methods

### Cloning of viral ORFeomes

The nucleotide sequences for all ORFs were obtained from the ncbi (http://www.ncbi.nlm.nih.gov/), and cloned into the Y2H vectors pGBKT7-DEST and pGADT7-DEST by recombinatorial cloning [55] (Protocol S1). All clones were sequence verified.

### Yeast two hybrid analysis

Yeast strains AH109 and Y187 were transformed using 1 µg of prey (pGADT7-DEST) or bait (pGBKT7-DEST) plasmid DNA, respectively, and grown on SD medium lacking either leucine (-leu) or tryptophane (-trp). Prey- and bait-expressing yeast were arrayed in a 384-pin format using a Biomek 2000 workstation (Beckman-Coulter) (4 replicas for each interaction tested), and mated in an all-against-all matrix approach [27]. Diploid colonies were grown for 2 days at 30°C on SD –leu-trp plates, and subsequently transferred to selective SD -leu-trp-his plates. Interactions were considered positive if at least 3 out of 4 colonies grew (Protocol S1).

### Co-immunoprecipitation

pGBKT7-DEST and pGADT7-DEST were co-transfected into HEK-293 cells by means of calcium phosphate, and superinfected with recombinant vaccinia virus (vTF-7) expressing T7 RNA polymerase (NIH AIDS repository) at a MOI of 10. After 24 h cells were lysed, and precipitation of proteins was done using 1 µg of either anti-myc (Santa Cruz) or anti-HA (Roche) antibodies in addition to protein G Sepharose beads. Precipitates were separated by SDS-PAGE, and western blots initially reacted with the anti-myc and anti-HA antibodies, and secondary, peroxidase-conjugated anti-mouse IgG or anti-rat IgG antibodies (Jackson). The CoIP was scored positive if a co-precipitate was detected in at least one direction (Protocol S1).

### Immunofluorescense

HeLa cells were grown on a cover slip until ∼50% confluence, and subsequently transfected with 1 µg of DNA for each of the fluorescent vectors analyzed, either alone or in combinations, by means of Effectene (Qiagen). Cells were incubated for 24 h, and fixed by incubating with 4% paraformaldehyde for 30 min at RT. Coverslips with fixed cells were mounted in Vectashield Mounting Medium (Vector Labs), and imaged on an OLYMPUS BX61 microscope.

### Text mining

Literature interactions were identified by combining automatic text mining and manual curation. A set of ∼87000 MEDLINE abstracts on herpesviruses was screened using ProMiner [56] for occurrences of proteins of any of the five viruses considered. Subsequently, 565 abstracts were selected containing a reference to interactions and at least two different proteins of the same virus. Physical interactions were then extracted manually from the corresponding articles.

### Network analysis

From the five individual networks an overlay network was created by merging orthologous proteins and interactions between orthologous proteins. Orthology relationships were assigned based on Davison [31]. The overlay network was then used to predict interactions between core proteins and to analyze network characteristics (Protocol S1).

### Sequence similarity

For all core orthologous proteins the average pairwise global sequence similarity across all five viruses was calculated. Global similarity was used to avoid a distortion of the results by short but high local similarities between orthologous proteins. For an interacting pair of core proteins, the similarity was calculated as the geometric mean of the average similarities for the corresponding proteins.

### Phylogenetic tree construction

The distance metric used to construct the phylogenetic tree (Figure 3E) for the complete and core network, respectively was based on the relative interaction overlaps. Accordingly, 
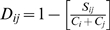
 where S_ij_ is the number of shared interactions between species i and j and C_i_ and C_j_ are the total number of interactions for species i and j. In this case, only interactions between proteins conserved in at least two species or all species for the core network were considered for each species-specific network. The phylogenetic tree was generated using the neighbour-joining algorithm of the PHYLIP package [57] with 10,000 bootstrap samples.

## Supporting Information

Protocol S1 (23 KB PDF)

Figure S1 Protein network in mCMV. The protein interaction network was generated using Cytoscape software (www.cytoscape.org) [58]. Interactions previously reported in the literature are indicated with red edges. The colours of the nodes indicate in which herpesviral species a specific protein is conserved.
(0.02 MB PDF)

Figure S2 Protein network in EBV. The protein interaction network was generated using Cytoscape software (www.cytoscape.org) [58]. Interactions previously reported in the literature are indicated with red edges. The colours of the nodes indicate in which herpesviral species a specific protein is conserved.
(0.09 MB PDF)

Figure S3 Network of reported protein interactions in HSV-1, VZV, mCMV, KSHV and EBV. Network structure of protein interactions reported previously for the five herpesvirus species which were identified by text mining and drawn with the Cytoscape software [58]. The width of the edges indicates for how many of the five species the interaction was reported. Interactions observed in our Y2H analysis are indicated with red edges.
(0.24 MB PDF)

Figure S4 Intraviral protein interactions in HSV-1, VZV, mCMV, EBV and KSHV. (A) Comparison of the number of proteins, number of proteins interacting with viral proteins, number of interactions, as well as the number of interactions previously reported in the literature with and without ortholog interactions. (B) Overlap (intersection of circles) between the Y2H results and previously reported protein interactions. Indicated are the absolute numbers of interactions reported in the literature (upper circle) or found by Y2H screens (lower circle).
(0.07 MB PDF)

Figure S5 Degree distribution in VZV, mCMV, KSHV and EBV. Node degree distribution for (A) VZV, (B) mCMV (C) EBV and (D) KSHV on a linear or logarithmic (inset) scale. The herpesviral networks can be approximated by power law distributions [23] (see also Table S3).
(0.01 MB PDF)

Figure S6 Attack tolerance of HSV-1, VZV, mCMV, KSHV and EBV networks. Simulations of deliberate attack on (A) VZV, (B) mCMV (C) EBV and (D) KSHV in comparison to two human networks by removing their most highly connected nodes (in decreasing order) [16],[17]. After each node is removed, the new network characteristic path length (average distance between any two nodes) of the remaining network is plotted as a multiple or fraction of the original parameters. The herpesviral networks consistently exhibited a higher attack tolerance, as the increase in path length is considerably smaller.
(0.01 MB PDF)

Figure S7 Overlap comparison to random orthology assignments. Distribution of the number of conserved interactions between all combinations of the five herpesvirus protein-protein interaction networks for 1000 random orthology assignments (blue line) compared to the true number of conserved interactions (red vertical line). For all combinations including (A) HSV-1 vs VZV, (B) HSV-1 vs EBV, (C) HSV-1 vs KSHV, (D) VZV vs mCMV, (E) VZV vs EBV, (F) VZV vs KSHV, (G) mCMV vs EBV, (h) mCMV vs KSHV and i) EBV vs KSHV the observed datasets show a significant increase in the number of conserved interactions.
(0.02 MB PDF)

Figure S8 Distribution of conserved interactions across subfamilies. The figure illustrates the distribution of interactions conserved in three species across subfamilies and compares it against the random expectation if all possible combinations are equally likely or weighted based on the number of interactions in the core of each species. Interactions are not preferentially conserved between closely related species and no significant difference to the random expectation can be observed.
(0.04 MB PDF)

Figure S9 Interologs in HSV-1, mCMV and EBV. (A) Interologs in HSV-1 (left), mCMV (middle) and EBV (right) were tested by CoIP using HA and myc tagged proteins expressed in 293T cells. The interaction between cellular proteins c-myc and max was used as a positive control. Positive CoIPs are indicated by asterisks. (B). Correlation between the number of species in which an interaction was found to be positive by Y2H, and the percentage of positive CoIPs. A straight line fitting the data showing a linearly increased rate of CoIP validation with the number of observed Y2H interactions.
(0.03 MB PDF)

Figure S10 Interspecies interactions between core proteins. A set of four interactions which were observed in at least two different species in the original Y2H screens were analysed for interspecies interactions in an all-against-all manner using both Y2H (A) and LUMIER (B). Positive interactions are indicated in green, while negative interactions are indicated in orange. The interactions observed in the original screens are indicated by red boxes. Two of the interactions were between structural components of the virion and are indicated as tegument and capsid. The other two interactions were chosen between proteins which either had a high or low sequence similarity (taken from Table S6).
(0.36 MB PDF)

Figure S11 Enrichment of interspecies interactions involving core proteins. For each interaction class (C = core and N = noncore) the number of positive interactions were divided on the total number of interactions tested. The fractions of positive interactions were enriched for the core-core and core-noncore classes compared to the noncore-noncore class. Significance was calculated by a chi-square test with 1 degree of freedom, and p-values<0.05 are indicated with an asterix.
(0.06 MB PDF)

Figure S12 Scheme of protein interactions in HSV-1 virions. The scheme indicates proteins that are present in HSV-1 virus particles. The central nodes indicate capsid proteins, the middle layer tegument proteins and the outer layer glycoproteins. The colour code is similar to Figure 1B. The edges indicate interactions detected in any of the five species and their width indicates the number of species in which the interaction was detected.
(0.00 MB PDF)

Figure S13 Protein interaction partners of HSV-1 UL33 tested by Y2H. HSV-1 UL33 cloned in pGADT7 (prey) was tested against a variety of interaction partners cloned in pGBKT7 (bait). As negative controls, each bait was tested against the empty pGADT7 prey vector (left plates) while the UL33 prey was tested against the empty pGBKT7 bait vector (right plates). (A) Evaluation of mated yeast clones on double and triple selective plates with empty pGADT7 vector used as a control. (B) Evaluation of mated yeast clones on increasing amounts of 3-AT (0, 2.5, 5, 10 mM) with empty pGADT7 vector as a control. Self-activation of UL15, UL16 and UL21 at 0 mM 3-AT was suppressed at 5 mM 3-AT, while the interactions with UL33 were still found to be positive.
(0.08 MB PDF)

Table S1 Summary of prey and bait hit-rates for HSV-1, mCMV and EBV. Overview of the total number of preys and baits included in the Y2H screens, including the number of preys and baits which yielded interactions. The total number of preys and baits exceed the total number of proteins tested due to many of the proteins being cloned as both fragments and full-length proteins.
(0.75 MB PDF)

Table S2 Protein interactions in HSV-1, VZV, mCMV, EBV and KSHV. List of interactions observed in the individual herpesviruses (bait-prey pairs). The interactions in VZV and KSHV have been reported by Uetz et al. [23].
(0.01 MB PDF)

Table S3 Comparison of Y2H results with published protein interactions. List of interactions found for HSV-1, VZV, HCMV, mCMV, EBV and KSHV through literature mining compared to interactions detected by Y2H.
(0.10 MB PDF)

Table S4Network parameters in herpesviruses. Topological parameters describing the network structure for all five viral interactomes. Self-interactions were not included for the computation of average clustering coefficients, characteristic path length and network diameter as well as enrichment values. For the enrichment over ES values, rewiring was performed 10^4^ times and clustering coefficients of the rewired networks were averaged. The expected clustering coefficient of the ER networks was computed as 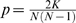 with *K* the total number of edges in the network and *N* the total number of nodes.(0.07 MB PDF)

Table S5 Ortholog proteins in five herpesvirus species. List of orthologous genes conserved between the five herpesviral species based on Davison et al [31].
(0.01 MB PDF)

Table S6 Average sequence similarity of core proteins between five herpesvirus species. Average sequence similarity for each of the core herpesviral proteins. The average similarities were calculated as specified in material and methods.
(0.02 MB PDF)

Table S7 Protein interactions between herpesvirus core orthologs. List of interactions observed between the 41 core proteins for all five species. The table indicates the number of species in which an interaction was observed, in addition to the specific species in which the interactions were observed.
(0.01 MB PDF)

Table S8 Average degree values for *core* and *non-core* proteins in all viruses. Average degree of core vs non-core proteins for all five interactomes. P-values were calculated with a Wilcoxon rank test between the degree values of core and non-core proteins.
(0.06 MB PDF)

Table S9 Ortholog protein interactions (predicted from KSHV) tested by Y2H and CoIP. List of orthologous interactions predicted from the KSHV interactome [23] which were tested by co-immunoprecipitation in HSV-1, mCMV and EBV. Results from the Y2H analysis of the predicted interactions are also indicated.
(0.00 MB PDF)

Table S10 Negatively predicted orthologous protein interactions (predicted from core interaction network) tested by Y2H and CoIP. Ten interactions were predicted to be negative, based on the fact that they were not observed in any of the five viral interactomes, and analysed by co-immunoprecipitation.
(0.02 MB PDF)

Table S11 Analysis of interspecies interactions. VZV core and noncore baits were analysed for Y2H interactions against prey libraries of VZV, HSV-1, mCMV, EBV and KSHV. The species of the interacting prey is included, in addition to whether it was a core or a noncore protein.
(0.02 MB PDF)

Table S12 M51 interactions tested by CoIP. Interactions observed with mCMV M51, or with other orthologs of M51, with a subset of interaction partners from the Y2H analysis. Tegument proteins, and other virion components, were determined based on whether they were reported to be present in the CMV virion [59],[60].
(0.01 MB PDF)

Table S13 Flat file containing the protein interactions in HSV-1, VZV, mCMV, EBV and KSHV. XL-sheet with the interactions observed in the individual herpesviruses (bait-prey pairs). The interactions in VZV and KSHV have been reported by Uetz et al. [23].
(0.01 MB XLS)

Table S14 Overview of previously published protein-protein interactions in HSV, VZV, HCMV, mCMV, EBV and KSHV. XL-sheet of previously published interactions mined from the literature (see material and methods). Each interaction is indicated with the method used and the pubmed ID of the publication.
(0.08 MB XLS)

## References

[1] Buckmaster AE, Scott SD, Sanderson MJ, Boursnell ME, Ross NL (1988). Gene sequence and mapping data from Marek's disease virus and herpesvirus of turkeys: implications for herpesvirus classification.. J Gen Virol.

[2] Fukuchi K, Sudo M, Lee YS, Tanaka A, Nonoyama M (1984). Structure of Marek's disease virus DNA: detailed restriction enzyme map.. J Virol.

[3] Cebrian J, Kaschka-Dierich C, Berthelot N, Sheldrick P (1982). Inverted repeat nucleotide sequences in the genomes of Marek disease virus and the herpesvirus of the turkey.. Proc Natl Acad Sci U S A.

[4] Davison AJ, Scott JE (1986). The complete DNA sequence of varicella-zoster virus.. JGenVirol.

[5] Chee MS, Bankier AT, Beck S, Bohni R, Brown CM (1990). Analysis of the protein-coding content of the sequence of human cytomegalovirus strain AD169.. CurrTopMicrobiolImmunol.

[6] McGeoch DJ, Gatherer D (2005). Integrating reptilian herpesviruses into the family herpesviridae.. J Virol.

[7] McGeoch DJ, Rixon FJ, Davison AJ (2006). Topics in herpesvirus genomics and evolution.. Virus Res.

[8] Yu D, Silva MC, Shenk T (2003). Functional map of human cytomegalovirus AD169 defined by global mutational analysis.. Proc Natl Acad Sci U S A.

[9] Dunn W, Chou C, Li H, Hai R, Patterson D (2003). Functional profiling of a human cytomegalovirus genome.. Proc Natl Acad Sci U S A.

[10] Song MJ, Hwang S, Wong WH, Wu TT, Lee S (2005). Identification of viral genes essential for replication of murine gamma-herpesvirus 68 using signature-tagged mutagenesis.. Proc Natl Acad Sci U S A.

[11] Uetz P, Giot L, Cagney G, Mansfield TA, Judson RS (2000). A comprehensive analysis of protein-protein interactions in Saccharomyces cerevisiae.. Nature.

[12] Li S, Armstrong CM, Bertin N, Ge H, Milstein S (2004). A map of the interactome network of the metazoan C. elegans.. Science.

[13] Giot L, Bader JS, Brouwer C, Chaudhuri A, Kuang B (2003). A protein interaction map of Drosophila melanogaster.. Science.

[14] LaCount DJ, Vignali M, Chettier R, Phansalkar A, Bell R (2005). A protein interaction network of the malaria parasite Plasmodium falciparum.. Nature.

[15] Rual JF, Venkatesan K, Hao T, Hirozane-Kishikawa T, Dricot A (2005). Towards a proteome-scale map of the human protein-protein interaction network.. Nature.

[16] Stelzl U, Worm U, Lalowski M, Haenig C, Brembeck FH (2005). A human protein-protein interaction network: a resource for annotating the proteome.. Cell.

[17] Bartel PL, Roecklein JA, SenGupta D, Fields S (1996). A protein linkage map of Escherichia coli bacteriophage T7.. Nat Genet.

[18] McCraith S, Holtzmann T, Moss B, Fields S (2000). Genome-wide analysis of vaccinia virus protein-protein interactions.. Proc Natl Acad Sci U S A.

[19] von Brunn A, Teepe C, Simpson JC, Pepperkok R, Friedel CC (2007). Analysis of Intraviral Protein-Protein Interactions of the SARS Coronavirus ORFeome.. PLoS ONE.

[20] Flajolet M, Rotondo G, Daviet L, Bergametti F, Inchauspe G (2000). A genomic approach of the hepatitis C virus generates a protein interaction map.. Gene.

[21] Choi IR, Stenger DC, French R (2000). Multiple interactions among proteins encoded by the mite-transmitted wheat streak mosaic tritimovirus.. Virology.

[22] Guo D, Rajamaki ML, Saarma M, Valkonen JP (2001). Towards a protein interaction map of potyviruses: protein interaction matrixes of two potyviruses based on the yeast two-hybrid system.. J Gen Virol.

[23] Uetz P, Dong Y, Zeretzke C, Atzler C, Baiker A (2006). Herpesviral protein networks and their interaction with the human proteome.. Science.

[24] Calderwood MA, Venkatesan K, Xing L, Chase MR, Vazquez A (2007). Epstein-Barr virus and virus human protein interaction maps.. Proc Natl Acad Sci U S A.

[25] Lee JH, Vittone V, Diefenbach E, Cunningham AL, Diefenbach RJ (2008). Identification of structural protein-protein interactions of herpes simplex virus type 1.. Virology.

[26] Rozen R, Sathish N, Li Y, Yuan Y (2008). Virion-wide protein interactions of Kaposi's sarcoma-associated herpesvirus.. J Virol.

[27] Cagney G, Uetz P, Fields S (2000). High-throughput screening for protein-protein interactions using two-hybrid assay.. Methods Enzymol.

[28] Huang H, Jedynak BM, Bader JS (2007). Where have all the interactions gone? Estimating the coverage of two-hybrid protein interaction maps.. PLoS Comput Biol.

[29] Grigoriev A (2003). On the number of protein-protein interactions in the yeast proteome.. Nucleic Acids Res.

[30] Hart GT, Ramani AK, Marcotte EM (2006). How complete are current yeast and human protein-interaction networks?. Genome Biol.

[31] Davison AJ (2004). Compendium of Human Herpesvirus gene names;.

[32] Gandhi TK, Zhong J, Mathivanan S, Karthick L, Chandrika KN (2006). Analysis of the human protein interactome and comparison with yeast, worm and fly interaction datasets.. NatGenet.

[33] Vittone V, Diefenbach E, Triffett D, Douglas MW, Cunningham AL (2005). Determination of interactions between tegument proteins of herpes simplex virus type 1.. J Virol.

[34] Barrios-Rodiles M, Brown KR, Ozdamar B, Bose R, Liu Z (2005). High-throughput mapping of a dynamic signaling network in mammalian cells.. Science.

[35] Schnee M, Ruzsics Z, Bubeck A, Koszinowski UH (2006). Common and specific properties of herpesvirus UL34/UL31 protein family members revealed by protein complementation assay.. J Virol.

[36] Fuchs W, Klupp BG, Granzow H, Osterrieder N, Mettenleiter TC (2002). The interacting UL31 and UL34 gene products of pseudorabies virus are involved in egress from the host-cell nucleus and represent components of primary enveloped but not mature virions.. J Virol.

[37] Reynolds AE, Ryckman BJ, Baines JD, Zhou Y, Liang L (2001). U(L)31 and U(L)34 proteins of herpes simplex virus type 1 form a complex that accumulates at the nuclear rim and is required for envelopment of nucleocapsids.. J Virol.

[38] Muranyi W, Haas J, Wagner M, Krohne G, Koszinowski UH (2002). Cytomegalovirus recruits cellular kinases to dissolve the nuclear lamina.. Science.

[39] Gonnella R, Farina A, Santarelli R, Raffa S, Feederle R (2005). Characterization and intracellular localization of the Epstein-Barr virus protein BFLF2: interactions with BFRF1 and with the nuclear lamina.. J Virol.

[40] Park R, Baines JD (2006). Herpes simplex virus type 1 infection induces activation and recruitment of protein kinase C to the nuclear membrane and increased phosphorylation of lamin B.. J Virol.

[41] Parrish JR, Yu J, Liu G, Hines JA, Chan JE (2007). A proteome-wide protein interaction map for Campylobacter jejuni.. Genome Biol.

[42] Boxem M, Maliga Z, Klitgord N, Li N, Lemmens I (2008). A protein domain-based interactome network for C. elegans early embryogenesis.. Cell.

[43] Abbotts AP, Preston VG, Hughes M, Patel AH, Stow ND (2000). Interaction of the herpes simplex virus type 1 packaging protein UL15 with full-length and deleted forms of the UL28 protein.. J Gen Virol.

[44] Thoma C, Borst E, Messerle M, Rieger M, Hwang JS (2006). Identification of the interaction domain of the small terminase subunit pUL89 with the large subunit pUL56 of human cytomegalovirus.. Biochemistry.

[45] Lischka P, Thomas M, Toth Z, Mueller R, Stamminger T (2007). Multimerization of human cytomegalovirus regulatory protein UL69 via a domain that is conserved within its herpesvirus homologues.. J Gen Virol.

[46] Zhi Y, Sciabica KS, Sandri-Goldin RM (1999). Self-interaction of the herpes simplex virus type 1 regulatory protein ICP27.. Virology.

[47] Yu H, Luscombe NM, Lu HX, Zhu X, Xia Y (2004). Annotation transfer between genomes: protein-protein interologs and protein-DNA regulogs.. Genome Res.

[48] Yamauchi Y, Wada K, Goshima F, Daikoku T, Ohtsuka K (2002). Herpes simplex virus type 2 UL14 gene product has heat shock protein (HSP)-like functions.. J Cell Sci.

[49] Lotzerich M, Ruzsics Z, Koszinowski UH (2006). Functional domains of murine cytomegalovirus nuclear egress protein M53/p38.. J Virol.

[50] Bubeck A, Wagner M, Ruzsics Z, Lotzerich M, Iglesias M (2004). Comprehensive mutational analysis of a herpesvirus gene in the viral genome context reveals a region essential for virus replication.. J Virol.

[51] al-Kobaisi MF, Rixon FJ, McDougall I, Preston VG (1991). The herpes simplex virus UL33 gene product is required for the assembly of full capsids.. Virology.

[52] Beard PM, Taus NS, Baines JD (2002). DNA cleavage and packaging proteins encoded by genes U(L)28, U(L)15, and U(L)33 of herpes simplex virus type 1 form a complex in infected cells.. The Journal of Virology.

[53] Wills E, Scholtes L, Baines JD (2006). Herpes simplex virus 1 DNA packaging proteins encoded by UL6, UL15, UL17, UL28, and UL33 are located on the external surface of the viral capsid.. J Virol.

[54] Loregian A, Palu G (2005). Disruption of protein-protein interactions: towards new targets for chemotherapy.. J Cell Physiol.

[55] Uetz P, Dong YA, Zeretzke C, Atzler C, Baiker A (2006). Herpesviral protein networks and their interaction with the human proteome.. Science.

[56] Hanisch D, Fundel K, Mevissen HT, Zimmer R, Fluck J (2005). ProMiner: rule-based protein and gene entity recognition.. BMC Bioinformatics.

[57] Felsenstein J (1989). PHYLIP: phylogeny interference package (version 3.2).. Cladistics.

[58] Shannon P, Markiel A, Ozier O, Baliga NS, Wang JT (2003). Cytoscape: a software environment for integrated models of biomolecular interaction networks.. Genome Res.

[59] Kattenhorn LM, Mills R, Wagner M, Lomsadze A, Makeev V (2004). Identification of proteins associated with murine cytomegalovirus virions.. J Virol.

[60] Varnum SM, Streblow DN, Monroe ME, Smith P, Auberry KJ (2004). Identification of proteins in human cytomegalovirus (HCMV) particles: the HCMV proteome.. J Virol.

