# Supplementary material for: Evolutionarily Conserved Herpesviral Protein Interaction Networks
Source: PLoS Pathog. 2009 Sep 4;5(9):e1000570. doi: 10.1371/journal.ppat.1000570 (PMC2731838; doi:10.1371/journal.ppat.1000570)
Supplement: Protocol S1 — (23 KB PDF) [file ppat.1000570.s001.pdf]

SUPPORTING INFORMATION

Cloning of viral ORFeomes

The nucleotide sequences for all ORFs were obtained from (http://www.ncbi.nlm.nih.gov/) with accession numbers NC_001806 (HSV-1 strain 17), NC_004065 (mCMV strain Smith), and AJ507799 (EBV strain B95-8). Each viral ORF was amplified by nested polymerase chain reaction (PCR) using either pHSVbluelox [1], pC3X [2] or B95.8/F [3] viral BAC DNA as template. The first part of the nested PCR was performed using the internal forward (AAAAAGCAGGCTCCGCCATGX_{18-30}) and reverse (AGAAAGCTGGGTT/C/ATAY_{18-30}) primers containing the internal attB1 and attB2 recombination sites. The gene-specific 5` forward sequence (X) matched the initiating codon continuing 15-27 nucleotides downstream, while the 3` reverse sequence (Y) matched 15-27 nucleotides ending in the stop codon. The second PCR was performed using external forward (GGGGACAAGTTTGTACAAAAAAGCAGGCT) and reverse (GGGGACCACTTTGTACAAGAAAGCTGGGT) primers which included the external parts of the attB1 and attB2 recombination sites. The resulting PCR-fragments, flanked by complete attB1 and attB2 sites, were cloned by BP-clonase into the gentamycin resistant entry vector pDONR207 (Gateway, Invitrogen). Viral proteins containing transmembrane domains were cloned both as full length and fragments.

Cloning destination vectors

The Y2H vectors pGBKT7-DEST and pGADT7-DEST were derived from pGBKT7 and pGADT7 by introducing a GATEWAY™ conversion cassette into the SmaI sites of each vector. Fluorescent fusion protein vectors pEYFP-N-DEST and pECFP-N-DEST were generated by PCR-amplifying EYFP and ECFP and, and cloning them into the BamHI and EcoRI sites of the pCR3 vector. The vectors were then made GATEWAY™

compatible by inserting a rfB-cassette (Invitrogen) into the EcoRV site of pCR3. pDsRed2-N-DEST (Clontech) was generated by cloning the rfB-cassette (Invitrogen) into the SmaI site of the vector.

Yeast-two-hybrid assay

Yeast strains AH109 and Y187 were transformed using 1 µg of prey (pGADT7-DEST) or bait (pGBKT7-DEST) plasmid DNA, respectively. Yeast cells were incubated for 1 h in 750 µl PEG/Bicine solution (40 % PEG 1000, 200 mM Bicine pH 8.35) at 30°C, followed by 5 min at 45°C. Subsequently, the yeast was pelleted and resuspended in 1 ml NP-buffer (0.15 M NaCl, 10 mM Bicine pH 8.35), pelleted a second time and resuspended in 200 µl NP-buffer and plated on to SD medium (+2 % agar) lacking either leucine (leu) (prey) or tryptophane (trp) (bait). Colonies were visible after 2-3 days. The yeast strains AH109 and Y187 expressing prey and bait proteins were arrayed in 96-deep-well plates with SD liquid media lacking leu or trp. A liquid bait culture was transferred onto SD medium plates lacking trp and the prey arrays were transferred onto the same plates for mating, using either a 384-pin replica tool (Nunc) or a Biomek 2000 workstation (Beckman-Coulter). Diploid colonies were grown for 2 days at 30°C and subsequently transferred to selective SD -leu-trp-his plates. Interactions were considered positive if at least 3 out of 4 colonies grew. A second reporter was not used as Braun and colleagues recently reported that this can reduce sensitivity without increasing specificity [4]. The cellular genes c-myc (pGBKT7) and max (pGADT7) were included as a positive control, while empty pGADT7 and pGBKT7 vectors were used as negative controls. Viral proteins acting as self-activating baits were analyzed on increasing amounts of 3-amino-1,2,4-triazole (Sigma) (3 mM, 10 mM, 25 mM) and excluded from the results if no clear positive interactions could be determined [5].

LUMIER

The LUMIER experiments were performed according to the protocol first described by Barrios-Rodiles and colleagues, with the exception of using a protein A tag (zz-domain) instead of a Flag tag [6].

Specificity of HSV-1 UL33 Y2H interactions

HSV-1 UL33 cloned in pGADT7 (prey) was tested against a variety of interaction partners cloned in pGBKT7 (bait) using increasing amounts of 3-AT (0, 2.5, 5, 10 mM). Mating with empty pGBKT7 was included as a negative control. The transformed yeast was incubated, mated and selected as mentioned above except being manually transferred between the different plates using a sterile inoculation loop. The positive interactions were not included in to the genome-wide HSV-1 interaction screen, and subsequently are not a part of any of the comparative analysis.

Co-immunoprecipitation

pGBKT7-DEST and pGADT7-DEST were co-transfected into HEK-293 cells using calcium phosphate, and superinfected with recombinant vaccinia virus (vTF-7) expressing T7 RNA polymerase (NIH AIDS repository) at a MOI of 10 in serum-free medium. As a positive control cells were transfected with cellular c-myc (pGBKT7) and max (pGADT7). After 24h cells were lysed by incubation in NP-40 lysis-buffer (1 % NP-40, 140 mM NaCl, 5mM $MgCl_2$, 20 mM Tris pH 7.6, 1 mM PMSF) on ice for 30 min. Lysates were centrifuged at 20.500 g for 10 min and precleared using 50 µl pre-equilibrated protein G-sepharose (1:1 slurry, Pharmacia). Lysates were precipitated using 1 µg of either anti-myc (Santa Cruz) or anti-HA (Roche) antibodies in addition to 50 µl protein G Sepharose beads (1:1 slurry) and incubated ON at 4°C. The beads were

washed 3 times in cold NP-40 buffer and resuspended in 2xSDS protein sample buffer. Precipitates were separated by SDS-PAGE using 8 – 15% gels. Western blots were first reacted with the anti-myc and anti-HA antibodies, and secondary, peroxidase-conjugated anti-mouse IgG or anti-rat IgG antibodies (Jackson). The CoIP was scored positive if a coprecipitate was detected in at least one direction.

Network analysis

From the five individual networks an overlay network was created by merging orthologous proteins and interactions between orthologous proteins. Orthology relationships were assigned based on Davison [7]. The overlay network was then used to predict interactions between *core* proteins and to analyze network characteristics. Overlaps between species were significantly increased compared to random orthology assignments (p-value < 0.001). No significant association between or within functional groups was observed using Fisher's exact test. As tegument proteins have a reputation of being sticky, we compared for each species the number of interactions of tegument proteins against all other proteins using the Mann-Whitney U test. We did not observe a significant difference in the amount of interaction partners of tegument proteins compared to other proteins for any of the five viruses which argues against tegument proteins being merely sticky in this study.

References

1. Nagel CH, Dohner K, Fathollahy M, Strive T, Borst EM, et al. (2008) Nuclear egress and envelopment of herpes simplex virus capsids analyzed with dual-color fluorescence HSV1(17+). J Virol 82: 3109-3124.
2. Messerle M, Crnkovic I, Hammerschmidt W, Ziegler H, Koszinowski UH (1997) Cloning and mutagenesis of a herpesvirus genome as an infectious bacterial artificial chromosome. Proc Natl Acad Sci U S A 94: 14759.

3. Delecluse HJ, Hilsendegen T, Pich D, Zeidler R, Hammerschmidt W (1998) Propagation and recovery of intact, infectious Epstein-Barr virus from prokaryotic to human cells. Proc Natl Acad Sci U S A 95: 8245.
4. Braun P, Tasan M, Dreze M, Barrios-Rodiles M, Lemmens I, et al. (2009) An experimentally derived confidence score for binary protein-protein interactions. Nat Methods 6: 91-97.
5. Cagney G, Uetz P, Fields S (2000) High-throughput screening for protein-protein interactions using two-hybrid assay. Methods Enzymol 328: 3.
6. Barrios-Rodiles M, Brown KR, Ozdamar B, Bose R, Liu Z, et al. (2005) High-throughput mapping of a dynamic signaling network in mammalian cells. Science 307: 1621.
7. Davison AJ. Compendium of Human Herpesvirus gene names; 2004; Reno.
