# Supplementary figures and images for: Evolutionarily Conserved Herpesviral Protein Interaction Networks
Source: PLoS Pathog. 2009 Sep 4;5(9):e1000570. doi: 10.1371/journal.ppat.1000570 (PMC2731838; doi:10.1371/journal.ppat.1000570)

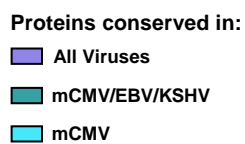

Supplement: Figure S1 — Protein network in mCMV. The protein interaction network was generated using Cytoscape software (www.cytoscape.org) [58]. Interactions previously reported in the literature are indicated with red edges. The colours of the nodes indicate in which herpesviral species a specific protein is conserved. (0.02 MB PDF) [file ppat.1000570.s002.pdf]

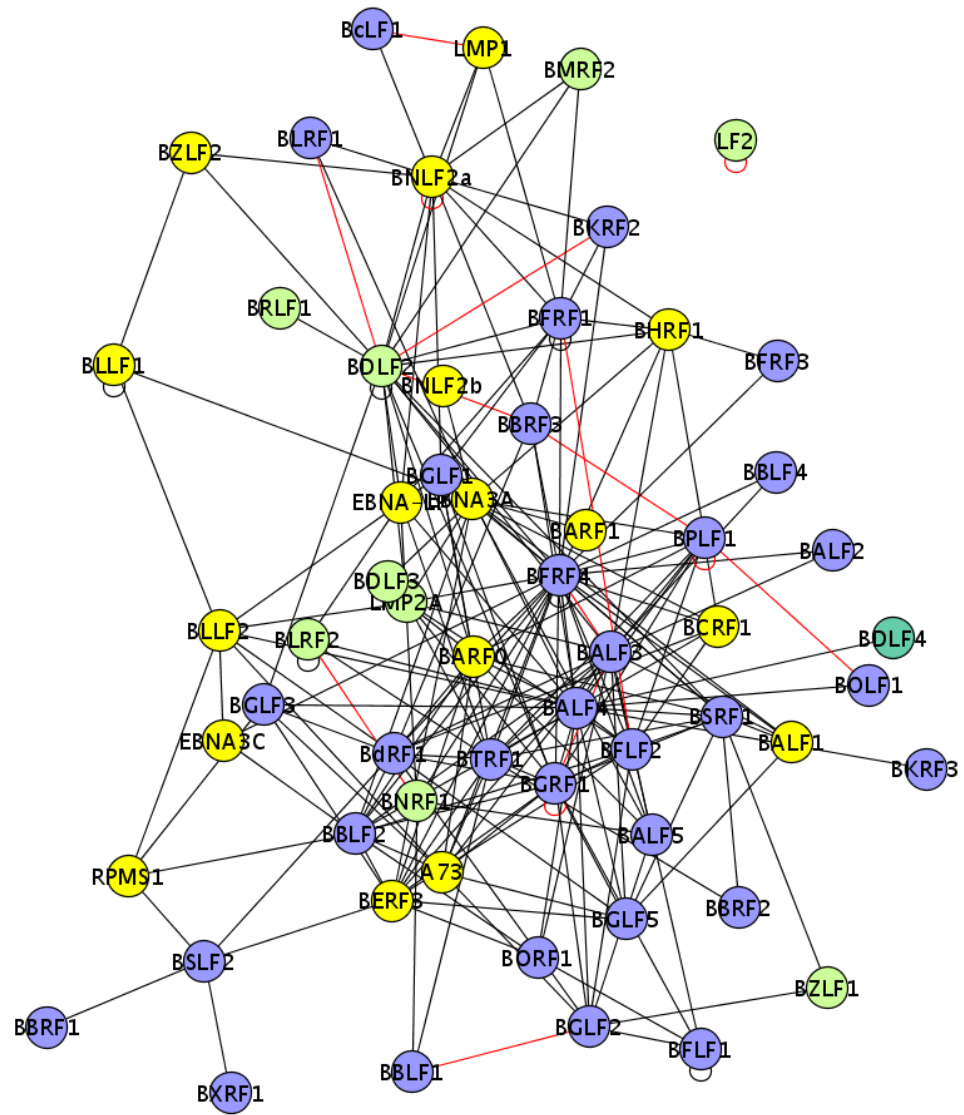

Proteins conserved in:

- All Viruses
- mCMV/EBV/KSHV
- EBV/KSHV
- EBV

Supplement: Figure S2 — Protein network in EBV. The protein interaction network was generated using Cytoscape software (www.cytoscape.org) [58]. Interactions previously reported in the literature are indicated with red edges. The colours of the nodes indicate in which herpesviral species a specific protein is conserved. (0.09 MB PDF) [file ppat.1000570.s003.pdf]

A

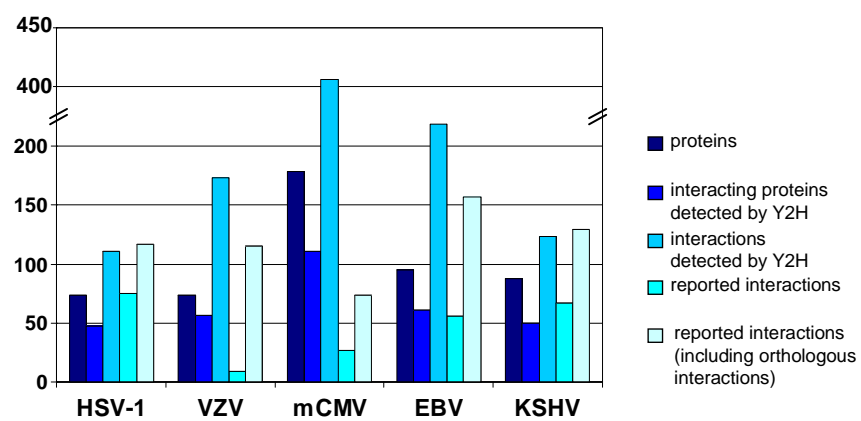

B

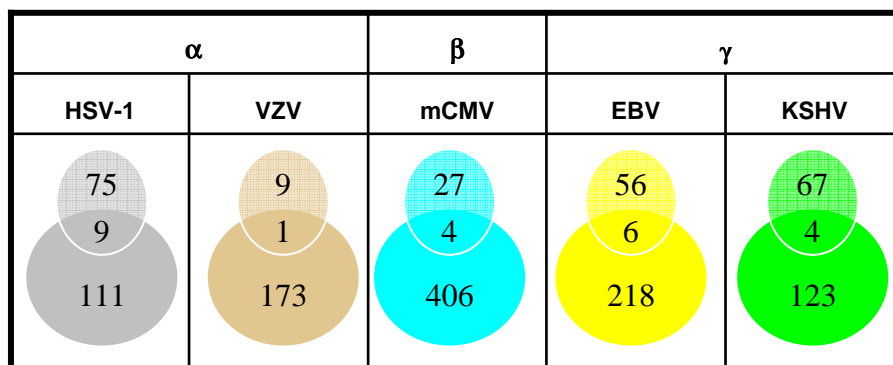

Supplement: Figure S4 — Intraviral protein interactions in HSV-1, VZV, mCMV, EBV and KSHV. (A) Comparison of the number of proteins, number of proteins interacting with viral proteins, number of interactions, as well as the number of interactions previously reported in the literature with and without ortholog interactions. (B) Overlap (intersection of circles) between the Y2H results and previously reported protein interactions. Indicated are the absolute numbers of interactions reported in the literature (upper circle) or found by Y2H screens (lower circle). (0.07 MB PDF) [file ppat.1000570.s005.pdf]

A

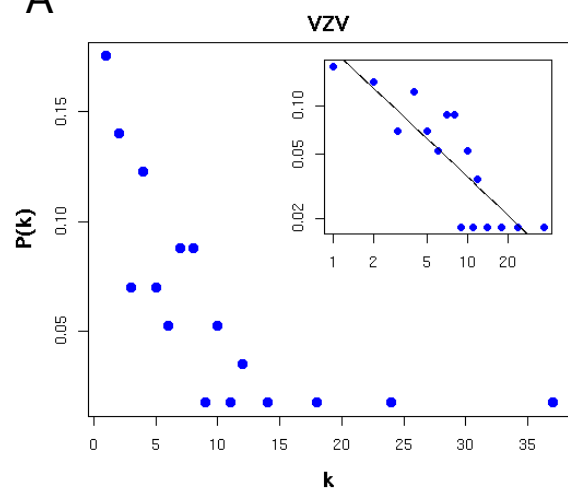

B

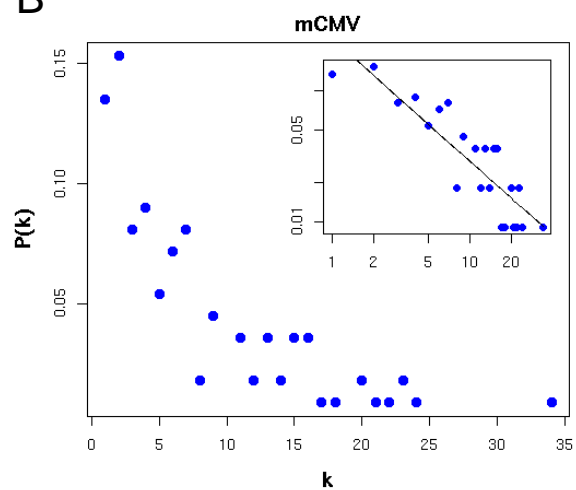

C

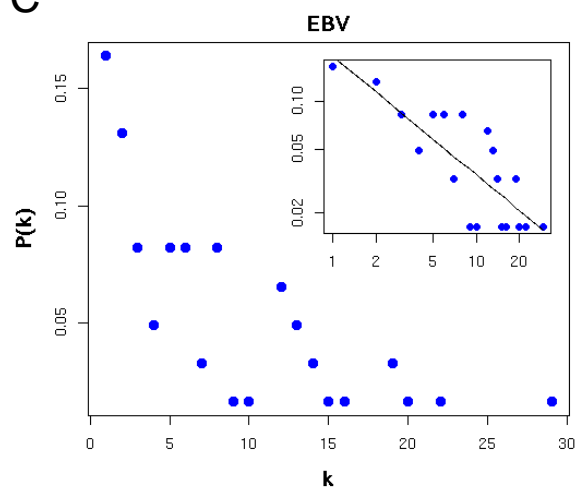

D

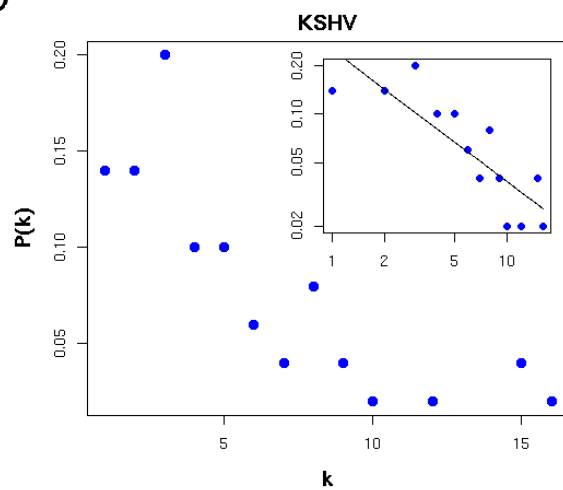

Supplement: Figure S5 — Degree distribution in VZV, mCMV, KSHV and EBV. Node degree distribution for (A) VZV, (B) mCMV (C) EBV and (D) KSHV on a linear or logarithmic (inset) scale. The herpesviral networks can be approximated by power law distributions [23] (see also Table S3). (0.01 MB PDF) [file ppat.1000570.s006.pdf]

A

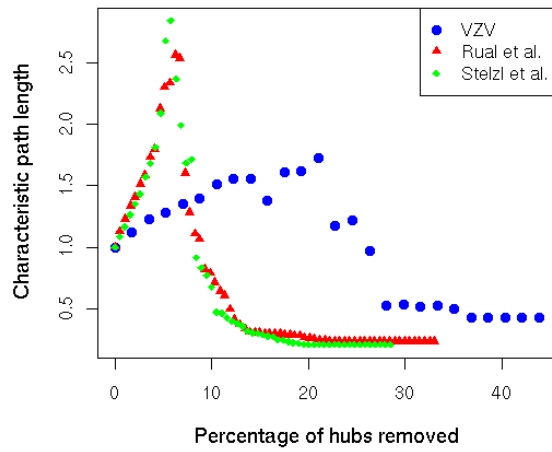

B

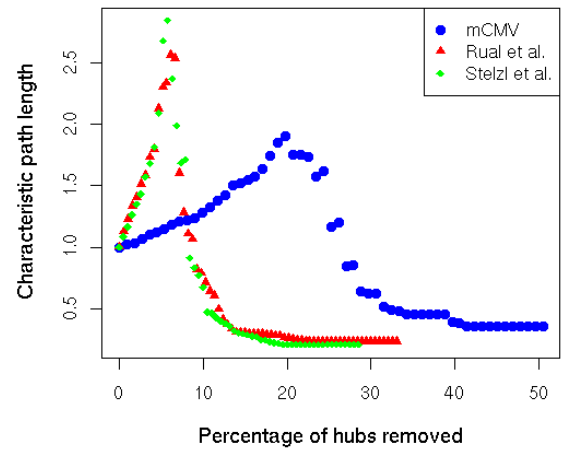

C

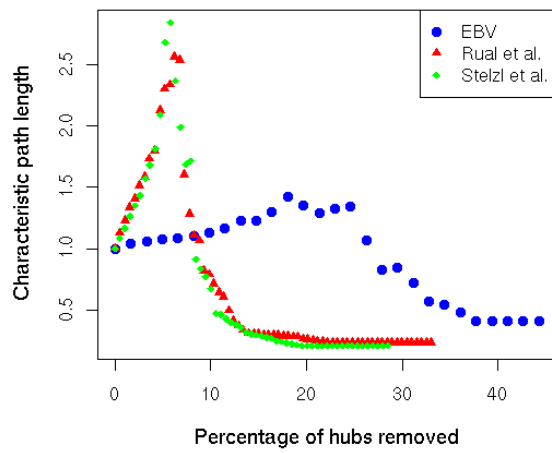

D

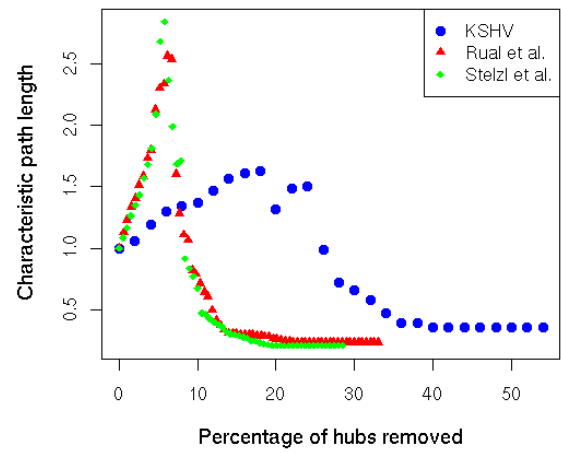

Supplement: Figure S6 — Attack tolerance of HSV-1, VZV, mCMV, KSHV and EBV networks. Simulations of deliberate attack on (A) VZV, (B) mCMV (C) EBV and (D) KSHV in comparison to two human networks by removing their most highly connected nodes (in decreasing order) [16],[17]. After each node is removed, the new network characteristic path length (average distance between any two nodes) of the remaining network is plotted as a multiple or fraction of the original parameters. The herpesviral networks consistently exhibited a higher attack tolerance, as the increase in path length is considerably smaller. (0.01 MB PDF) [file ppat.1000570.s007.pdf]

A

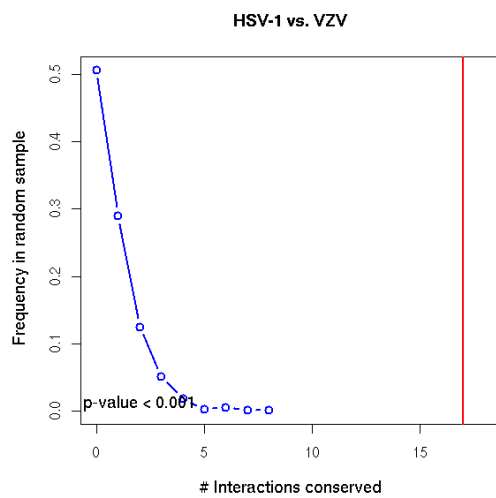

B

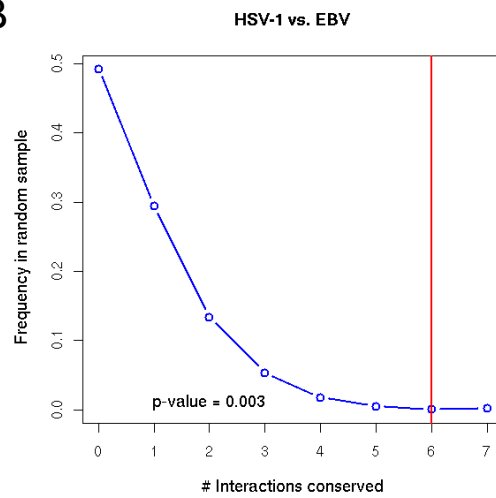

C

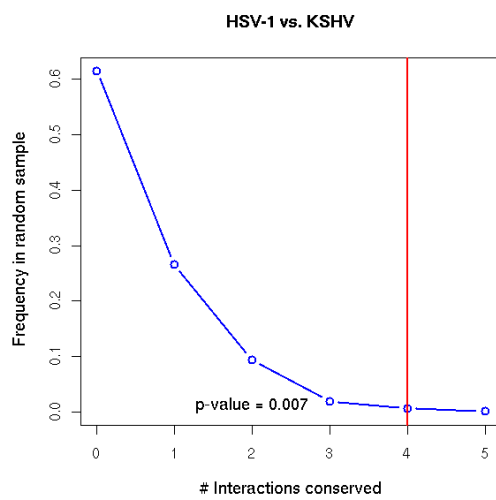

D

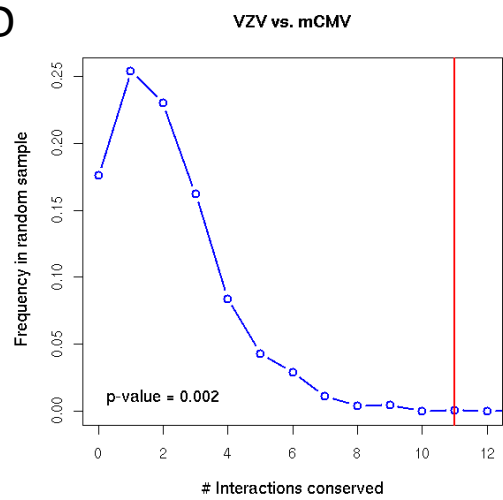

E

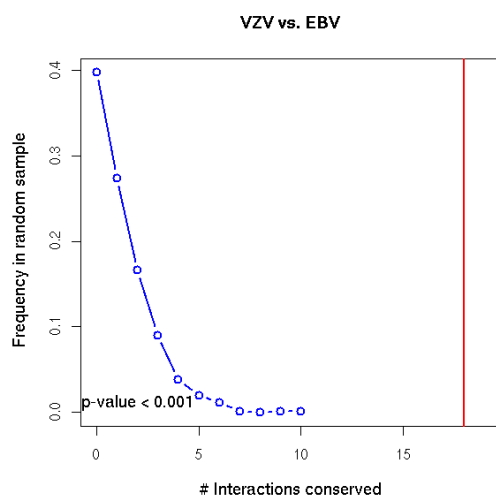

F

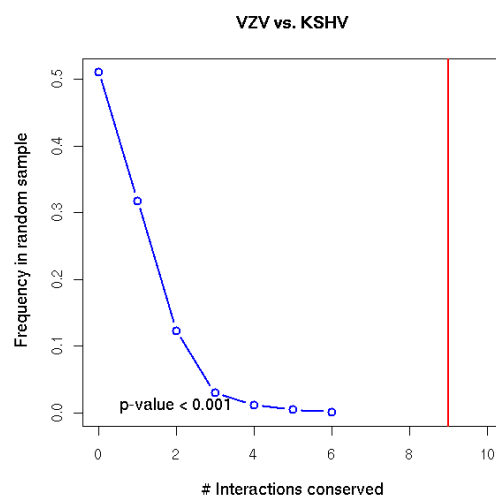

G

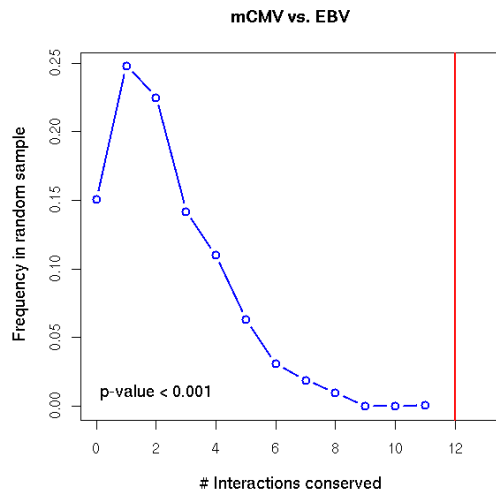

H

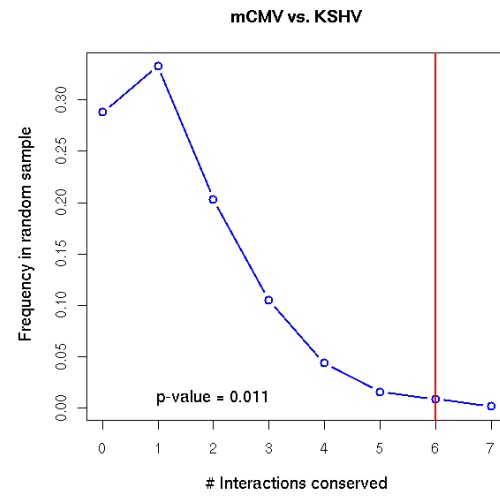

I

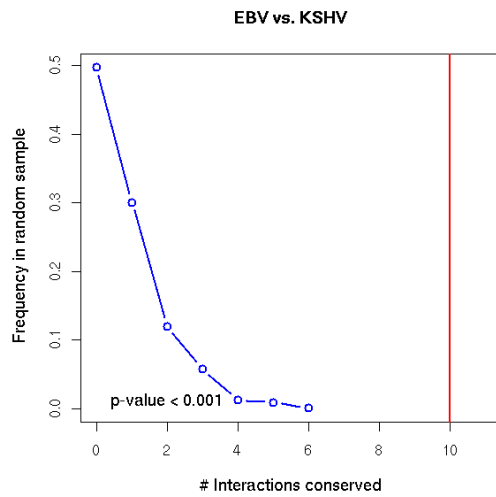

Supplement: Figure S7 — Overlap comparison to random orthology assignments. Distribution of the number of conserved interactions between all combinations of the five herpesvirus protein-protein interaction networks for 1000 random orthology assignments (blue line) compared to the true number of conserved interactions (red vertical line). For all combinations including (A) HSV-1 vs VZV, (B) HSV-1 vs EBV, (C) HSV-1 vs KSHV, (D) VZV vs mCMV, (E) VZV vs EBV, (F) VZV vs KSHV, (G) mCMV vs EBV, (h) mCMV vs KSHV and i) EBV vs KSHV the observed datasets show a significant increase in the number of conserved interactions. (0.02 MB PDF) [file ppat.1000570.s008.pdf]

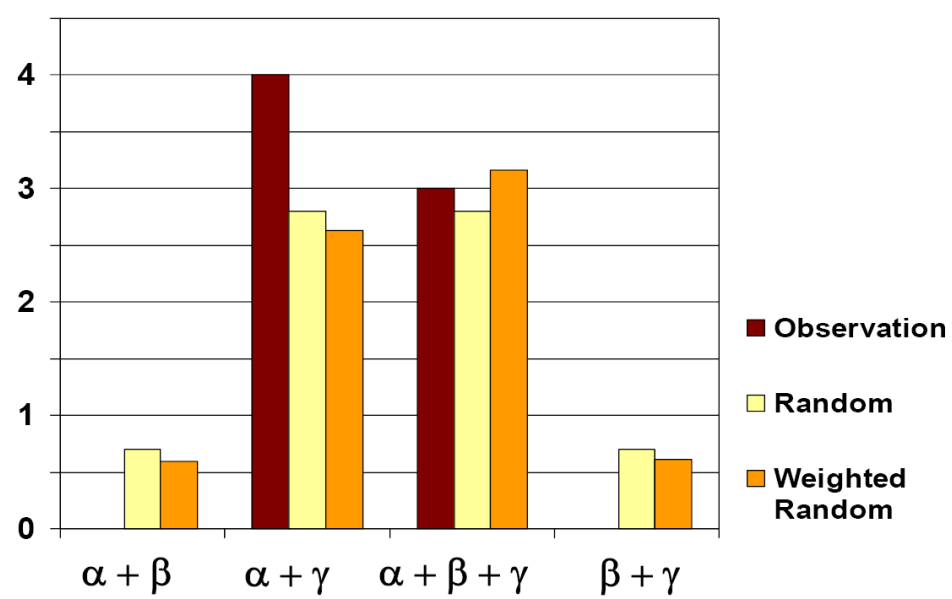

Supplement: Figure S8 — Distribution of conserved interactions across subfamilies. The figure illustrates the distribution of interactions conserved in three species across subfamilies and compares it against the random expectation if all possible combinations are equally likely or weighted based on the number of interactions in the core of each species. Interactions are not preferentially conserved between closely related species and no significant difference to the random expectation can be observed. (0.04 MB PDF) [file ppat.1000570.s009.pdf]

**A**

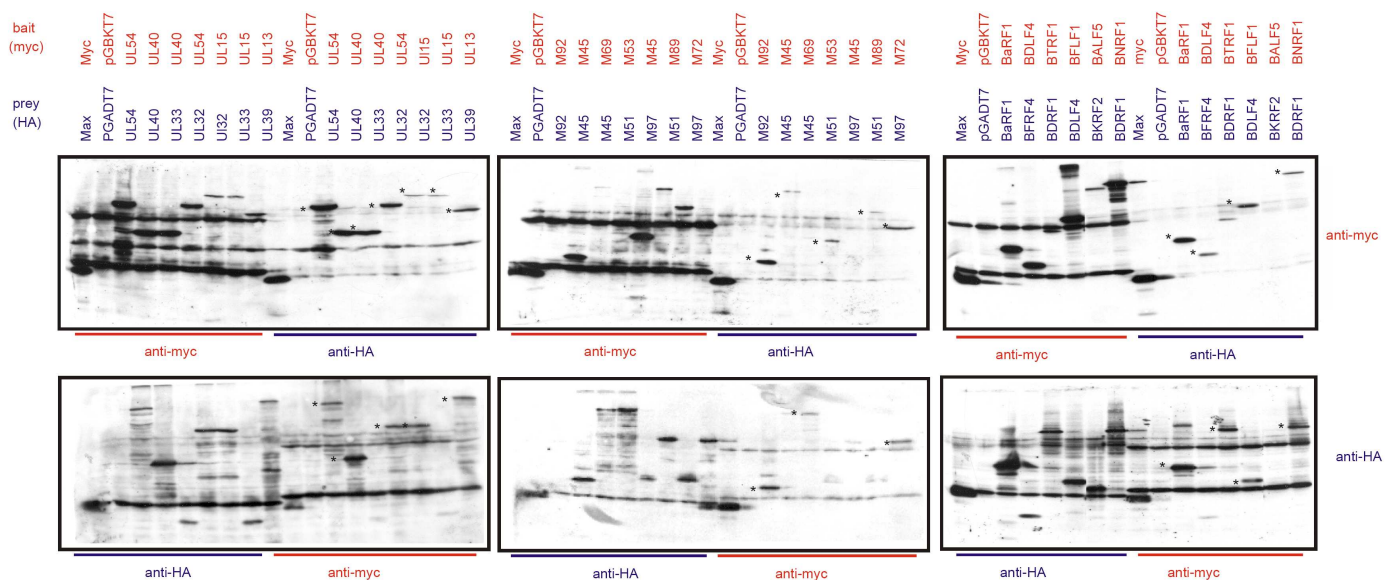

**B**

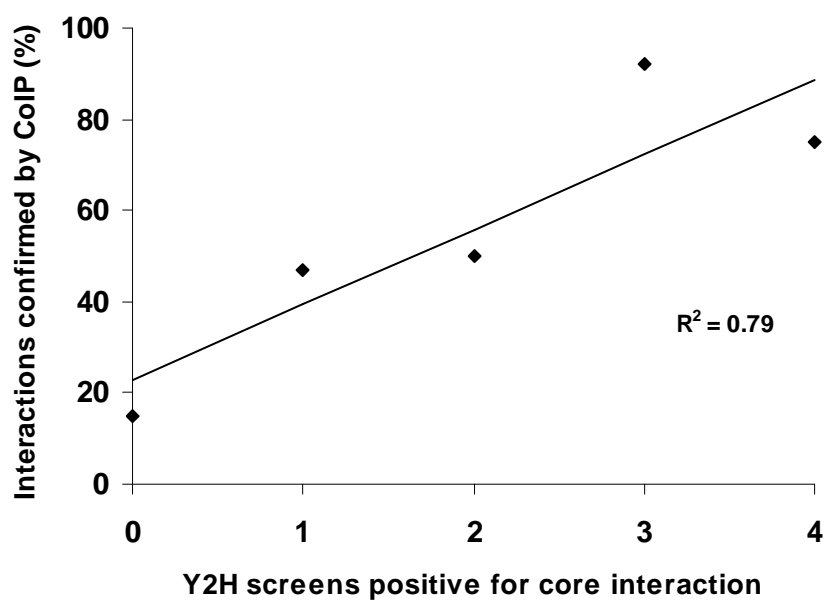

Supplement: Figure S9 — Interologs in HSV-1, mCMV and EBV. (A) Interologs in HSV-1 (left), mCMV (middle) and EBV (right) were tested by CoIP using HA and myc tagged proteins expressed in 293T cells. The interaction between cellular proteins c-myc and max was used as a positive control. Positive CoIPs are indicated by asterisks. (B). Correlation between the number of species in which an interaction was found to be positive by Y2H, and the percentage of positive CoIPs. A straight line fitting the data showing a linearly increased rate of CoIP validation with the number of observed Y2H interactions. (0.03 MB PDF) [file ppat.1000570.s010.pdf]

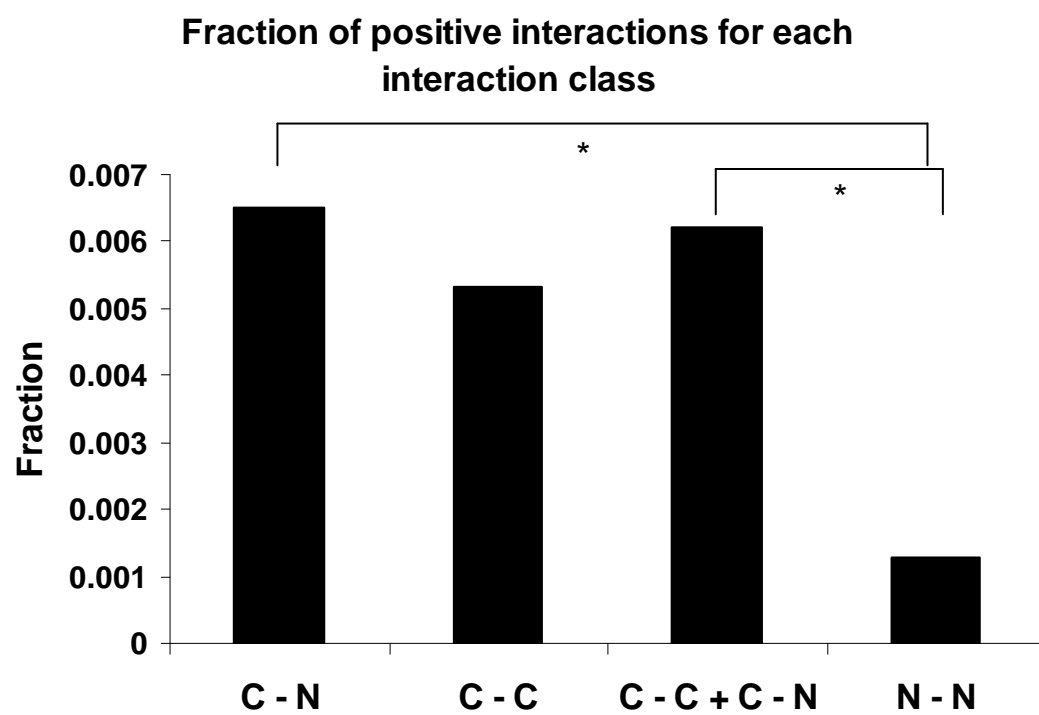

C = core, N = noncore

\* =  $p < 0.05$

Supplement: Figure S11 — Enrichment of interspecies interactions involving core proteins. For each interaction class (C = core and N = noncore) the number of positive interactions were divided on the total number of interactions tested. The fractions of positive interactions were enriched for the core-core and core-noncore classes compared to the noncore-noncore class. Significance was calculated by a chi-square test with 1 degree of freedom, and p-values<0.05 are indicated with an asterix. (0.06 MB PDF) [file ppat.1000570.s012.pdf]

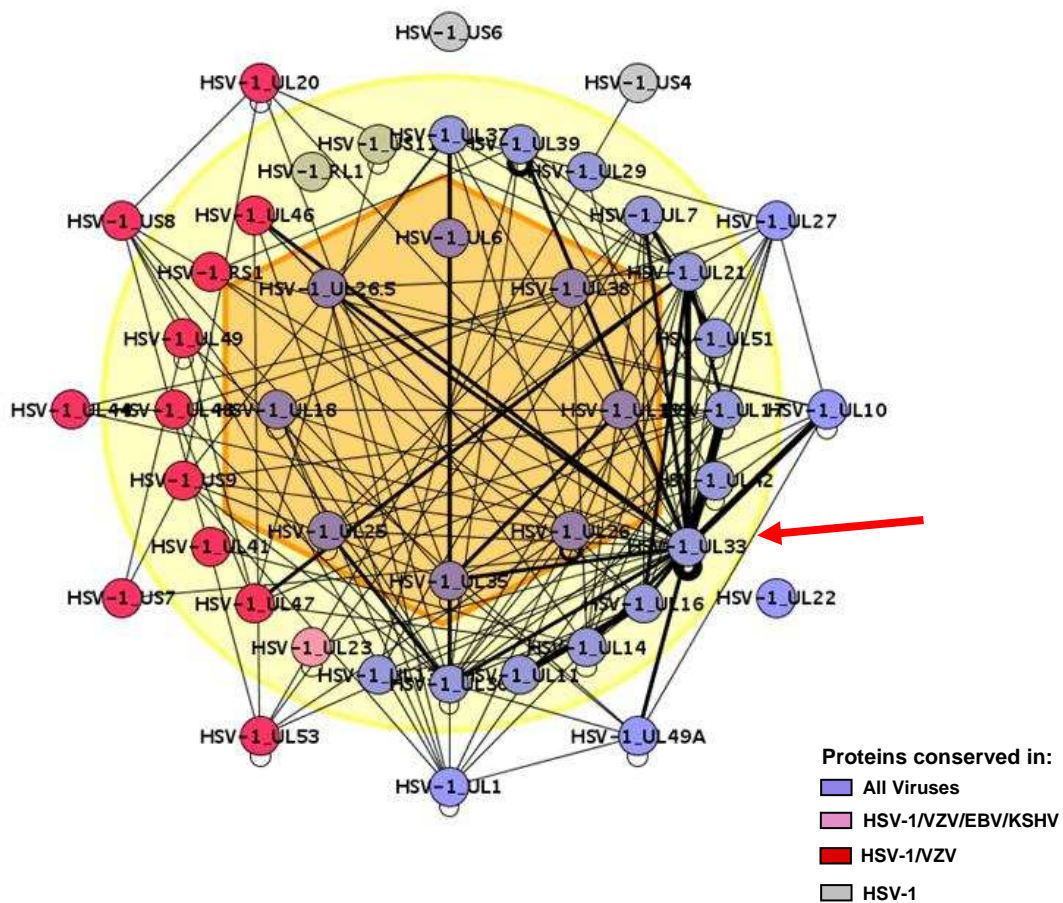

Supplement: Figure S12 — Scheme of protein interactions in HSV-1 virions. The scheme indicates proteins that are present in HSV-1 virus particles. The central nodes indicate capsid proteins, the middle layer tegument proteins and the outer layer glycoproteins. The colour code is similar to Figure 1B. The edges indicate interactions detected in any of the five species and their width indicates the number of species in which the interaction was detected. (0.00 MB PDF) [file ppat.1000570.s013.pdf]

A

+pGADT7

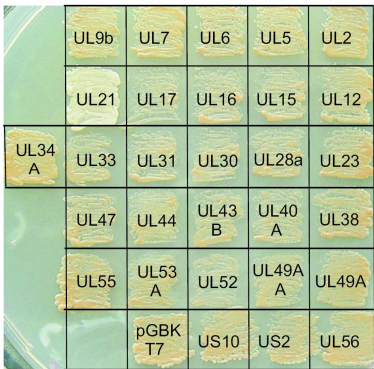

SDC  
-leu  
-trp

+pGADT7-UL33

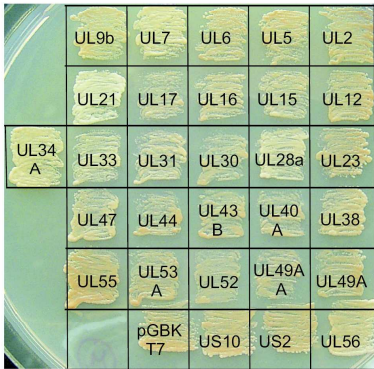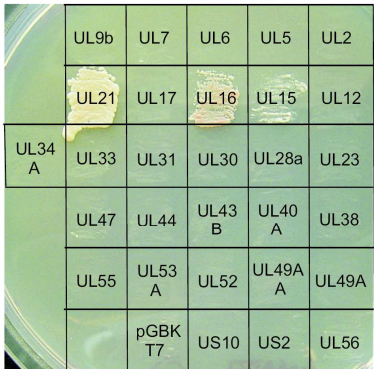

SDC  
-leu  
-trp  
-his

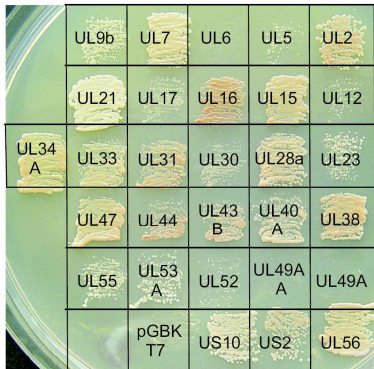

B

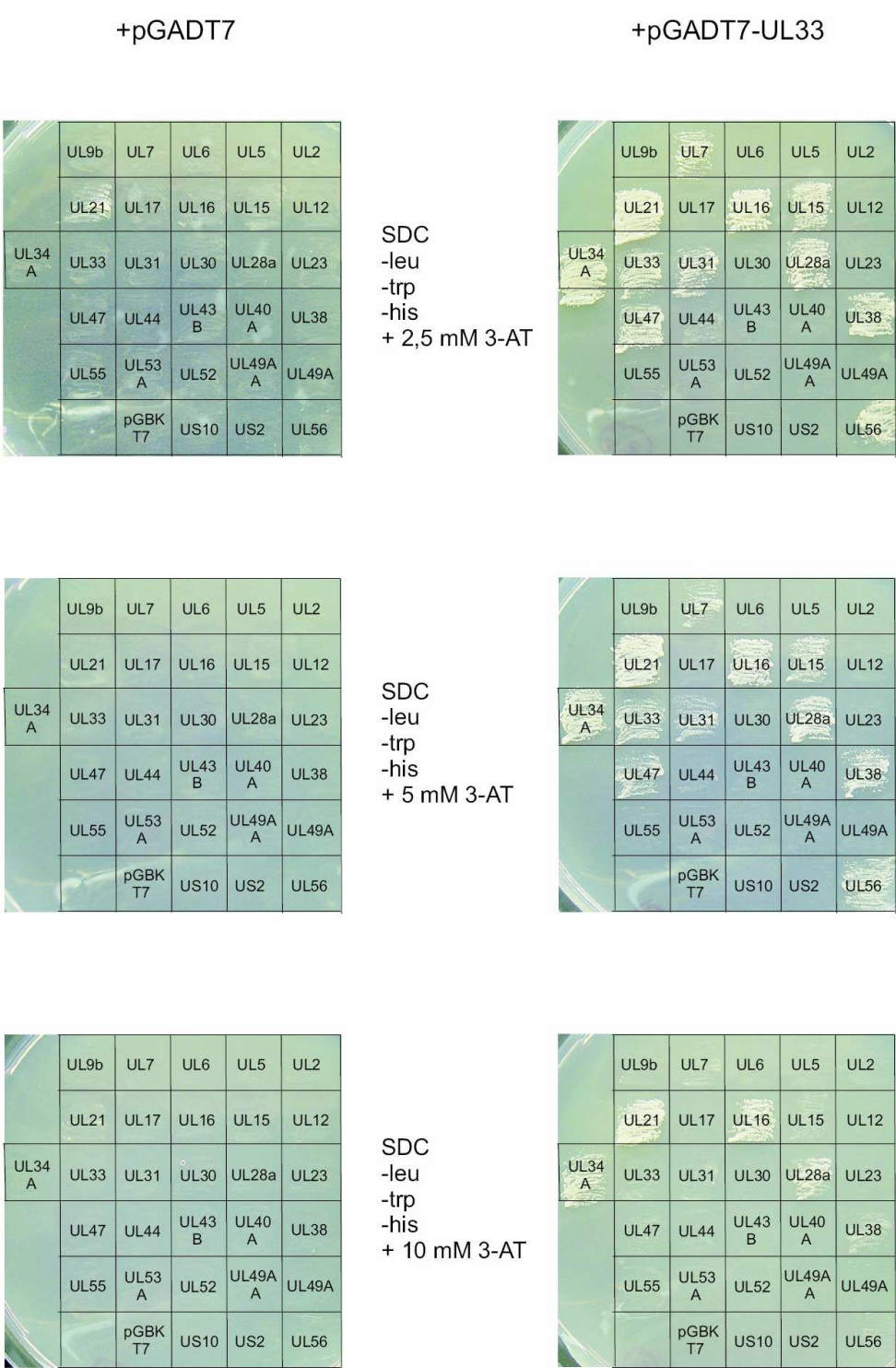

Supplement: Figure S13 — Protein interaction partners of HSV-1 UL33 tested by Y2H. HSV-1 UL33 cloned in pGADT7 (prey) was tested against a variety of interaction partners cloned in pGBKT7 (bait). As negative controls, each bait was tested against the empty pGADT7 prey vector (left plates) while the UL33 prey was tested against the empty pGBKT7 bait vector (right plates). (A) Evaluation of mated yeast clones on double and triple selective plates with empty pGADT7 vector used as a control. (B) Evaluation of mated yeast clones on increasing amounts of 3-AT (0, 2.5, 5, 10 mM) with empty pGADT7 vector as a control. Self-activation of UL15, UL16 and UL21 at 0 mM 3-AT was suppressed at 5 mM 3-AT, while the interactions with UL33 were still found to be positive. (0.08 MB PDF) [file ppat.1000570.s014.pdf]
