# Supplementary material for: Evolutionarily Conserved Herpesviral Protein Interaction Networks
Source: PLoS Pathog. 2009 Sep 4;5(9):e1000570. doi: 10.1371/journal.ppat.1000570 (PMC2731838; doi:10.1371/journal.ppat.1000570)
Supplement: Figure S10 — Interspecies interactions between core proteins. A set of four interactions which were observed in at least two different species in the original Y2H screens were analysed for interspecies interactions in an all-against-all manner using both Y2H (A) and LUMIER (B). Positive interactions are indicated in green, while negative interactions are indicated in orange. The interactions observed in the original screens are indicated by red boxes. Two of the interactions were between structural components of the virion and are indicated as tegument and capsid. The other two interactions were chosen between proteins which either had a high or low sequence similarity (taken from Table S6). (0.36 MB PDF) [file ppat.1000570.s011.pdf]

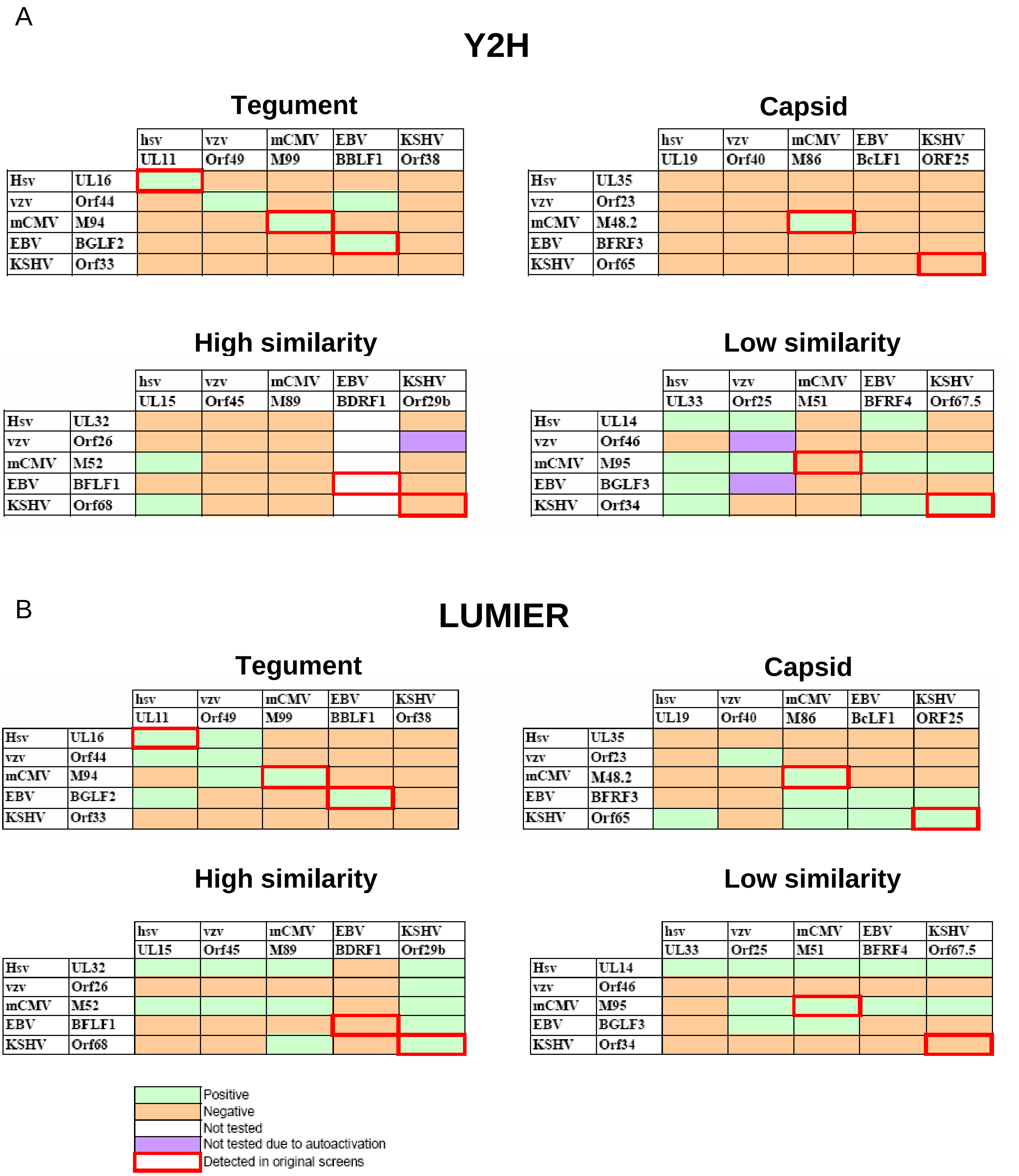
A
Y2H
Tegument
hsv UL11 | vzv Orf49 | mCMV M99 | EBV BBLF1 | KSHV Orf38
Hsv UL16
vzv Orf44
mCMV M94
EBV BGLF2
KSHV Orf33
Capsid
hsv UL19 | vzv Orf40 | mCMV M86 | EBV BcLF1 | KSHV ORF25
Hsv UL35
vzv Orf23
mCMV M48.2
EBV BFRF3
KSHV Orf65
High similarity
hsv UL15 | vzv Orf45 | mCMV M89 | EBV BDRF1 | KSHV Orf29b
Hsv UL32
vzv Orf26
mCMV M52
EBV BFLF1
KSHV Orf68
Low similarity
hsv UL33 | vzv Orf25 | mCMV M51 | EBV BFRF4 | KSHV Orf67.5
Hsv UL14
vzv Orf46
mCMV M95
EBV BGLF3
KSHV Orf34
B
LUMIER
Tegument
hsv UL11 | vzv Orf49 | mCMV M99 | EBV BBLF1 | KSHV Orf38
Hsv UL16
vzv Orf44
mCMV M94
EBV BGLF2
KSHV Orf33
Capsid
hsv UL19 | vzv Orf40 | mCMV M86 | EBV BcLF1 | KSHV ORF25
Hsv UL35
vzv Orf23
mCMV M48.2
EBV BFRF3
KSHV Orf65
High similarity
hsv UL15 | vzv Orf45 | mCMV M89 | EBV BDRF1 | KSHV Orf29b
Hsv UL32
vzv Orf26
mCMV M52
EBV BFLF1
KSHV Orf68
Low similarity
hsv UL33 | vzv Orf25 | mCMV M51 | EBV BFRF4 | KSHV Orf67.5
Hsv UL14
vzv Orf46
mCMV M95
EBV BGLF3
KSHV Orf34
Positive
Negative
Not tested
Not tested due to autoactivation
Detected in original screens
