# Supplementary material for: Evolutionarily Conserved Herpesviral Protein Interaction Networks
Source: PLoS Pathog. 2009 Sep 4;5(9):e1000570. doi: 10.1371/journal.ppat.1000570 (PMC2731838; doi:10.1371/journal.ppat.1000570)
Supplement: Table S1 — Summary of prey and bait hit-rates for HSV-1, mCMV and EBV. Overview of the total number of preys and baits included in the Y2H screens, including the number of preys and baits which yielded interactions. The total number of preys and baits exceed the total number of proteins tested due to many of the proteins being cloned as both fragments and full-length proteins. (0.75 MB PDF) [file ppat.1000570.s015.pdf]

Table S1: Summary of prey and bait hit-rates for HSV-1, mCMV and EBV

	HSV	mCMV	EBV
**# total preys**	95	284	155
**# interacting preys**	34	94	38
**# total baits**	94	283	155
**# interacting baits**	48	114	54
**# tested ORFs**	70	173	100
**# interacting ORFs**	48	111	61
