# Supplementary material for: Evolutionarily Conserved Herpesviral Protein Interaction Networks
Source: PLoS Pathog. 2009 Sep 4;5(9):e1000570. doi: 10.1371/journal.ppat.1000570 (PMC2731838; doi:10.1371/journal.ppat.1000570)
Supplement: Table S2 — Protein interactions in HSV-1, VZV, mCMV, EBV and KSHV. List of interactions observed in the individual herpesviruses (bait-prey pairs). The interactions in VZV and KSHV have been reported by Uetz et al. [23]. (0.01 MB PDF) [file ppat.1000570.s016.pdf]

Table S2: Protein interactions in HSV-1, VZV, mCMV, EBV and KSHV.

		HSV-1			VZV[1]			mCMV			EBV			KSHV [23]	
1		RL2	UL49		1	25		M1	M88		A73	BALF3		6	52
2		UL2	UL12		1	27		M3	M9		A73	BARF0		6	K15
3		UL2	UL14		1	60		M3	M10		A73	BBLF2		23	28
4		UL2	UL33		1	62		M3	M50		A73	BDRF1		23	29b
5		UL2	UL37		3	16		M3	M55		A73	BFRF4		23	30
6		UL2	UL40		3	39		M3	M72		A73	BGLF2		23	45
7		UL2	UL53		3	46		M3	M80		A73	BGLF3		23	57
8		UL3	UL4		4	38		M3	M106		A73	BGLF5		23	60
9		UL7	UL7		7	7		M3	M119.2		A73	BLLF2		23	63
10		UL7	UL14		7	53		M3	M119.3		A73	BTRF1		23	K09
11		UL7	UL15		8	19		M7	M125		BALF1	BALF3		25	65
12		UL7	UL16		9	9a		M7	M163		BALF1	BFRF4		27	58
13		UL7	UL33		9	56		M7	M164		BALF1	BGLF5		28	28
14		UL7	UL45		9	67		M7	M169		BALF1	BSRF1		28	29b
15		UL7	UL53		9a	22		M8	M50		BALF1	EBNA3A		28	30
16		UL7	US2		9a	23		M10	M95		BALF2	BALF3		28	K11
17		UL9	UL15		9a	56		M11	M26		BALF2	BFRF4		29b	50
18		UL9	UL33		10	57		M11	M72		BALF3	BGLF5		29b	54
19		UL9	UL45		12	33		M11	M87		BALF3	BORF1		29b	72
20		UL10	UL49A		12	33.5		M11	M93		BALF3	BTRF1		29b	K08.1
21		UL10	US8A		16	16		M11	M125		BALF4	BALF3		29b	K10.5
22		UL11	UL16		16	19		M11	M126		BALF4	BARF0		30	29b
23		UL14	UL14		16	24		M11	M162		BALF4	BBLF2		31	30
24		UL16	UL14		16	33		M11	M168		BALF4	BBRF3		31	31
25		UL16	UL21		16	42		M14	M119.3		BALF4	BCRF1		31	41
26		UL16	UL33		16	59		M17	M3		BALF4	BDLF4		31	67.5
27		UL17	UL33		16	64		M17	M9		BALF4	BDRF1		31	68
28		UL17	UL45		18	33.5		M17	M10		BALF4	BFLF2		31	K11
29		UL21	UL20		18	41		M17	M13		BALF4	BFRF4		36	48
30		UL23	UL40		18	59		M17	M14		BALF4	BGLF2		36	56
31		UL23	UL45		18	65		M17	M48.2		BALF4	BGLF3		36	61
32		UL26	UL45		19	18		M17	M55		BALF4	BGLF5		37	K08
33		UL26	UL53		19	19		M17	M72		BALF4	BKRF3		39	9
34		UL28	UL33		19	43		M17	M106		BALF4	BLLF2		41	9
35		UL28	UL40		21	33		M17	M107		BALF4	BLRF2		41	28
36		UL28	UL45		21	33.5		M17	M119.2		BALF4	BOLF1		41	29b
37		UL28	UL53		21	42		M17	M119.3		BALF4	BORF1		41	63
38		UL28	US2		21	65		M17	M126		BALF4	BPLF1		45	36
39		UL30	UL14		22	21		M17	M163		BALF4	BSRF1		45	50
40		UL30	UL33		22	33		M17	M164		BALF4	BTRF1		45	72
41		UL30	UL40		22	33.5		M17	M169		BALF4	EBNA3A		47	9
42		UL30	UL45		22	34		M18	M119.2		BALF4	EBNA-LP		50	57
43		UL30	UL53		22	41		M18	M163		BALF5	BALF3		50	75
44		UL31	UL34		22	43		M20	M40		BALF5	BARF0		52	34
45		UL31	UL45		22	59		M20	M119.2		BALF5	BFRF4		52	49
46		UL33	UL33		22	65		M22	M48.2		BALF5	BGLF2		52	52
47		UL34	UL14		22	68		M22	M72		BALF5	BNRF1		52	57
48		UL36	UL48		23	18		M22	M87		BALF5	EBNA3A		52	59
49		UL38	UL14		23	21		M22	M88		BARF0	BFRF4		52	60
50		UL38	UL18		23	65		M22	M93		BARF1	EBNA3A		52	69
51		UL38	UL33		23	68		M22	M107		BBLF1	BGLF2		54	36
52		UL38	UL45		24	52		M22	M125		BBLF1	BTRF1		54	62
53		UL38	UL48		25	2		M22	M126		BBLF2	BALF3		56	60
54		UL40	UL14		25	3		M22	M128		BBLF2	BARF0		57	57
55		UL40	UL15		25	8		M22	M134		BBLF2	BDRF1		60	60
56		UL40	UL33		25	9a		M22	M142		BBLF2	BFLF2		60	K01

		HSV-1			VZV[1]			mCMV			EBV			KSHV [23]	
57		UL40	UL37		25	12		M22	M144		BBLF2	BFRF4		61	57
58		UL40	UL40		25	15		M22	M162		BBLF2	BORF1		61	60
59		UL40	UL53		25	18		M22	M168		BBLF3	EBNA3A		61	61
60		UL43	UL33		25	19		M22	M169		BBLF4	BALF3		65	63
61		UL43	UL40		25	24		M23.1	M29.1		BBLF4	BFRF4		67.5	9
62		UL43	UL45		25	25		M23.1	M51		BBRF1	BMLF1		67.5	23
63		UL43	UL49A		25	27		M23.1	M87		BBRF2	BTRF1		67.5	28
64		UL43	UL53		25	30		M23.1	M88		BBRF3	BDLF2		67.5	29b
65		UL43	US2		25	32		M25.1a	M168		BBRF3	BFRF1		67.5	34
66		UL44	UL17		25	33		M25.2	M69		BBRF3	BNLF2a		67.5	59
67		UL44	UL21		25	33.5		M26	M126		BcLF1	BNLF2a		67.5	60
68		UL44	UL33		25	36		M26	M168		BCRF1	EBNA3A		67.5	63
69		UL44	UL40		25	38		M28	M106		BDLF2	BALF3		67.5	69
70		UL45	UL45		25	39		M28	M119.3		BDLF2	BARF0		67.5	75
71		UL45	UL53		25	41		M28	M169		BDLF2	BDLF2		68	9
72		UL46	UL33		25	42		M29.1	M29.1		BDLF2	BDRF1		68	29b
73		UL46	UL45		25	43		M29.1	M30		BDLF2	BFRF4		68	57
74		UL46	UL53		25	44		M29.1	M51		BDLF2	BGLF3		68	59
75		UL47	UL14		25	49		M29.1	M72		BDLF2	BSRF1		68	60
76		UL47	UL15		25	50		M29.1	M97		BDLF3	EBNA3A		68	75
77		UL47	UL17		25	51		M29.1	M106		BdRF1	BALF3		72	37
78		UL47	UL21		25	52		M29.1	M107		BdRF1	BARF0		74	27
79		UL47	UL40		25	55		M29.1	M127		BdRF1	BBLF2		74	29b
80		UL47	UL48		25	56		M29.1	M144		BdRF1	BBRF3		K03	53
81		UL47	UL49		25	57		M29.1	M162		BdRF1	BCRF1		K03	60
82		UL47	US1		25	59		M29.1	M169		BdRF1	BDRF1		K03	K03
83		UL47	US11		25	64		M30	M106		BdRF1	BFRF4		K03	K07
84		UL49	UL49		25	65		M30	M144		BdRF1	BGLF2		K05	6
85		UL49	US8		25	67		M32	M168		BdRF1	BGLF3		K05	28
86		UL49A	UL15		25	68		M35	M106		BdRF1	BGLF5		K05	34
87		UL49A	UL33		25	S/L		M35	M144		BdRF1	BLLF2		K05	53
88		UL49A	UL49A		26	26		M36	M25.1a		BdRF1	BMLF1		K05	59
89		UL53	UL33		27	9a		M36	M126		BdRF1	BTRF1		K05	60
90		UL53	UL53		27	11		M36	M144		BDRF1	BALF3		K07	74
91		UL54	UL54		27	16		M36	M163		BDRF1	BDRF1		K07	K05
92		UL55	UL33		27	19		M36	M164		BDRF1	BGLF2		K08	57
93		UL55	UL45		27	21		M36	M168		BDRF1	BGLF5		K08	60
94		UL55	UL53		27	22		M36	M169		BDRF1	BTRF1		K08.1	75
95		UL56	UL21		27	23		M37	M10		BERF3	BALF3		K09	63
96		UL56	UL49		27	24		M37	M55		BERF3	BARF0		K09	69
97		UL56	US1		27	27		M37	M119.2		BERF3	BBLF2		K10	2
98		UL56	US11		27	33		M37	M144		BERF3	BDRF1		K10	9
99		US1	US7		27	33.5		M37	M163		BERF3	BFLF2		K10	28
100		US2	UL33		27	34		M37	M169		BERF3	BFRF4		K10	29b
101		US2	UL45		27	38		M40	M106		BERF3	BGLF3		K10	31
102		US2	UL53		27	42		M40	M119.3		BERF3	BGLF5		K10	37
103		US2	US2		27	43		M40	M146		BERF3	BMLF1		K10	39
104		US4	UL53		27	50		M40	M168		BERF3	BORF1		K10	41
105		US5	UL43		27	60		M41	M9		BERF3	BTRF1		K10	47
106		US8	UL53		27	62		M41	M50		BERF3	EBNA3A		K10	49
107		US10	UL14		27	64		M41	M72		BFLF1	BDRF1		K10	59
108		US10	UL23		27	65		M41	M119.2		BFLF1	BFLF1		K10	60
109		US10	UL33		27	68		M41	M147		BFLF1	BFLF2		K10	61
110		US10	UL45		33	33		M43	M29.1		BFLF1	BGLF2		K10	67.5
111		US11	US11		33	33.5		M43	M87		BFLF1	BORF1		K10	68
112					33	66		M43	M88		BFLF2	BALF3		K10	K12
113					34	34		M43	M93		BFLF2	BCRF1		K10.5	56
114					34	42		M43	M103		BFLF2	BDRF1		K10.5	75
115					36	36		M43	M144		BFLF2	BFRF4		K11	34

		HSV-1			VZV[1]			mCMV			EBV			KSHV [23]	
116					36	S/L		M43	M168		BFLF2	BGLF2		K11	29b
117					38	11		M45	M45		BFRF1	BDLF2		K11	59
118					38	19		M45	M48		BFRF1	BFLF2		K11	60
119					38	26		M45	M51		BFRF1	BFRF1		K11	61
120					38	44		M45	M72		BFRF1	BFRF4		K11	69
121					39	8		M45	M168		BFRF3	BFRF4		K12	29b
122					39	23		M48	M10		BFRF4	BALF3		K12	60
123					39	24		M48	M94		BFRF4	BBRF3		K12	K12
124					39	32		M48	M137		BFRF4	BCRF1			
125					39	39		M48.1	M51		BFRF4	BDRF1			
126					39	42		M48.2	M48.2		BFRF4	BFRF4			
127					39	59		M48.2	M51		BFRF4	BTRF1			
128					39	64		M48.2	M86		BGLF1	BARF0			
129					39	65		M48.2	M87		BGLF1	BDRF1			
130					39	68		M48.2	M91		BGLF1	BFRF1			
131					39	S/L		M48.2	M119.2		BGLF1	BFRF4			
132					42	65		M50	M10		BGLF1	BLLF2			
133					43	65		M50	M14		BGLF1	BNLF2a			
134					44	61		M50	M55		BGLF1	BTRF1			
135					46	17		M50	M119.2		BGLF1	EBNA3A			
136					46	24		M50	M119.3		BHRF1	BALF3			
137					46	26		M50	M126		BHRF1	BDLF2			
138					46	38		M50	M168		BHRF1	BFRF1			
139					46	45		M50	M169		BHRF1	BFRF3			
140					50	50		M51	M48		BHRF1	BFRF4			
141					56	14		M51	M51		BHRF1	BNLF2a			
142					56	33		M51	M54		BHRF1	EBNA3A			
143					56	33.5		M51	M97		BKRF2	BDLF2			
144					56	41		M51	M126		BKRF2	BFRF1			
145					56	65		M52	M75		BKRF2	BFRF4			
146					60	3		M52	M97		BKRF2	BNLF2a			
147					60	9a		M53	M50		BLLF1	BLLF1			
148					60	12		M53	M51		BLLF1	BLLF2			
149					60	15		M53	M169		BLLF1	EBNA3A			
150					60	23		M55	M125		BLRF1	BDLF2			
151					60	24		M55	M163		BLRF1	BNLF2a			
152					60	28		M55	M164		BLRF1	EBNA3A			
153					60	33		M56	M1		BLRF2	BLRF2			
154					60	33.5		M56	M14		BLRF2	EBNA-LP			
155					60	41		M69	M69		BMRF2	BDLF2			
156					60	42		M69	M72		BMRF2	BFRF1			
157					60	43		M69	M89		BMRF2	BNLF2a			
158					60	60		M72	M51		BNLF2a	BDLF2			
159					60	65		M73	M55		BNLF2a	BFRF1			
160					60	68		M73	M119.3		BNLF2a	BNLF2a			
161					61	50		M73	M168		BNLF2b	EBNA3A			
162					61	55		M73	M169		BNRF1	BBLF1			
163					61	61		M77	M48		BNRF1	BFRF4			
164					61	68		M77	M51		BNRF1	BLRF2			
165					62	4		M77	M88		BNRF1	EBNA-LP			
166					62	19		M77	M103		BOLF1	BPLF1			
167					62	24		M77	M126		BPLF1	BALF3			
168					62	44		M77	M168		BPLF1	BARF0			
169					62	50		M80	M80		BPLF1	BBRF3			
170					62	61		M84	M23.1		BPLF1	BCRF1			
171					62	S/L		M84	M33		BPLF1	BDRF1			
172					66	33.5		M85	M50		BPLF1	BFLF2			
173					67	65		M85	M77		BPLF1	BFRF4			
174								M85	M85		BPLF1	BHRF1			

		HSV-1			VZV[1]			mCMV			EBV			KSHV [23]	
175								M86	M48.1		BPLF1	BPLF1			
176								M87	M163		BPLF1	BTRF1			
177								M87	M164		BPLF1	EBNA3A			
178								M89	M93		BRLF1	BDLF2			
179								M90	M35		BSRF1	BARF0			
180								M90	M51		BSRF1	BBRF2			
181								M90	M72		BSRF1	BDRF1			
182								M90	M88		BSRF1	BFLF2			
183								M90	M93		BSRF1	BFRF4			
184								M90	M103		BSRF1	BGLF5			
185								M90	M107		BSRF1	BTRF1			
186								M90	M119.2		BSRF1	EBNA3A			
187								M90	M144		BTRF1	BLRF2			
188								M90	M168		BTRF1	EBNA-LP			
189								M90	M169		BXRF1	BMLF1			
190								M90	M29.1		BZLF1	BGLF2			
191								M93	M50		BZLF1	BSRF1			
192								M93	M51		BZLF2	BDLF2			
193								M93	M72		BZLF2	BLLF1			
194								M93	M77		BZLF2	BNLF2a			
195								M93	M85		EBNA3C	BBLF2			
196								M93	M87		EBNA3C	BFRF4			
197								M93	M88		EBNA3C	BLLF2			
198								M93	M93		EBNA-LP	BDLF2			
199								M93	M106		EBNA-LP	BFRF1			
200								M93	M107		EBNA-LP	BNLF2a			
201								M93	M25.1a		LF2	LF2			
202								M94	M99		LMP1	BcLF1			
203								M95	M51		LMP1	BDLF2			
204								M97	M54		LMP1	BFRF1			
205								M97	M95		LMP1	BNLF2a			
206								M98	M97		LMP2A	BALF3			
207								M98	M106		LMP2A	BARF0			
208								M98	M114		LMP2A	BDRF1			
209								M99	M10		LMP2A	BFLF2			
210								M99	M35		LMP2A	BFRF4			
211								M99	M72		LMP2A	BLLF2			
212								M99	M90		LMP2A	BTRF1			
213								M99	M106		LMP2A	EBNA3A			
214								M99	M119.2		LMP2B	BDLF2			
215								M100	M26		RPMS1	BBLF2			
216								M100	M48.2		RPMS1	BGLF3			
217								M100	M51		RPMS1	BLLF2			
218								M100	M72		RPMS1	BMLF1			
219								M100	M73.5						
220								M100	M87						
221								M100	M168						
222								M103	M51						
223								M103	M53						
224								M103	M89						
225								M103	M97						
226								M108	M25.1a						
227								M108	M26						
228								M108	M48.2						
229								M108	M51						
230								M108	M72						
231								M108	M87						
232								M108	M88						
233								M108	M93						

		HSV-1			VZV[1]			mCMV			EBV			KSHV [23]	
234								M108	M103						
235								M117.1	M3						
236								M117.1	M9						
237								M117.1	M10						
238								M117.1	M13						
239								M117.1	M14						
240								M117.1	M55						
241								M117.1	M91						
242								M117.1	M106						
243								M117.1	M119.2						
244								M117.1	M119.3						
245								M117.1	M163						
246								M117.1	M164						
247								M117.1	M169						
248								M119.1	M26						
249								M119.1	M48.2						
250								M119.1	M51						
251								M119.1	M72						
252								M119.1	M87						
253								M119.1	M126						
254								M119.1	M152						
255								M119.1	M155						
256								M119.1	M162						
257								M119.1	M163						
258								M119.1	M168						
259								M119.2	M10						
260								M119.2	M12						
261								M119.2	M14						
262								M119.2	M55						
263								M119.2	M71						
264								M119.2	M72						
265								M119.2	M91						
266								M119.2	M106						
267								M119.2	M119.2						
268								M119.2	M125						
269								M119.2	M128						
270								M119.2	M136						
271								M119.2	M164						
272								M119.2	M169						
273								M119.3	M119.2						
274								M119.4	M29.1						
275								M119.4	M126						
276								M119.4	M152						
277								M119.4	M155						
278								M119.5	M87						
279								M119.5	M88						
280								M119.5	M162						
281								M119.5	M168						
282								M120	M125						
283								M120	M126						
284								M120	M127						
285								M120	M144						
286								M120	M168						
287								M120	M169						
288								M124	M10						
289								M124	M106						
290								M124	M119.3						
291								M124	M169						
292								M124.1	M50						

		HSV-1			VZV[1]			mCMV			EBV			KSHV [23]	
293								M125	M72						
294								M125	M126						
295								M125	M163						
296								M125	M168						
297								M125	M169						
298								M126	M72						
299								M126	M87						
300								M126	M88						
301								M126	M126						
302								M126	M168						
303								M127	M78						
304								M127	M88						
305								M127	M93						
306								M127	M103						
307								M130	M71						
308								M131	M26						
309								M131	M126						
310								M131	M134						
311								M131	M48.2						
312								M131	M87						
313								M134	M9						
314								M134	M48.2						
315								M134	M126						
316								M134	M168						
317								M135	M119.3						
318								M138	M2						
319								M138	M3						
320								M138	M9						
321								M138	M10						
322								M138	M12						
323								M138	M13						
324								M138	M14						
325								M138	M20						
326								M138	M29.1						
327								M138	M30						
328								M138	M40						
329								M138	M48.2						
330								M138	M55						
331								M138	M72						
332								M138	M107						
333								M138	M119.2						
334								M138	M119.3						
335								M138	M119.5						
336								M138	M164						
337								M138	M169						
338								M139	M26						
339								M140	M50						
340								M140	M72						
341								M140	M88						
342								M140	M93						
343								M140	M107						
344								M140	M124.1						
345								M140	M126						
346								M140	M128						
347								M140	M136						
348								M140	M141						
349								M140	M168						
350								M142	M126						
351								M142	M128						

		HSV-1			VZV[1]			mCMV			EBV			KSHV [23]	
352								M142	M143						
353								M142	M161						
354								M142	M168						
355								M144	M144						
356								M146	M48.2						
357								M147	M72						
358								M150	M40						
359								M150	M136						
360								M150	M169						
361								M151	M9						
362								M151	M10						
363								M151	M13						
364								M151	M14						
365								M151	M48.2						
366								M151	M51						
367								M151	M55						
368								M151	M72						
369								M151	M87						
370								M151	M119.2						
371								M151	M119.3						
372								M151	M162						
373								M154	M119.2						
374								M154	M169						
375								M155	M119.2						
376								M159	M3						
377								M159	M10						
378								M159	M13						
379								M159	M14						
380								M159	M55						
381								M159	M91						
382								M159	M106						
383								M159	M119.3						
384								M159	M163						
385								M159	M164						
386								M159	M169						
387								M161	M10						
388								M161	M13						
389								M161	M55						
390								M161	M106						
391								M161	M119.2						
392								M162	M26						
393								M162	M48.2						
394								M162	M72						
395								M162	M88						
396								M162	M125						
397								M162	M126						
398								M162	M134						
399								M162	M162						
400								M162	M168						
401								M163	M119.2						
402								M163	M119.3						
403								M163	M163						
404								M163	M169						
405								M168	M119.2						
406								M170	M78						
