# Supplementary material for: Evolutionarily Conserved Herpesviral Protein Interaction Networks
Source: PLoS Pathog. 2009 Sep 4;5(9):e1000570. doi: 10.1371/journal.ppat.1000570 (PMC2731838; doi:10.1371/journal.ppat.1000570)
Supplement: Table S3 — Comparison of Y2H results with published protein interactions. List of interactions found for HSV-1, VZV, HCMV, mCMV, EBV and KSHV through literature mining compared to interactions detected by Y2H. (0.10 MB PDF) [file ppat.1000570.s017.pdf]

Table S3: Comparison of Y2H results with published protein interactions.

	Protein 1						Protein 2					Confirmed interactions					# species
	Species	HSV-1	VZV	mCMV	EBV	KSHV	HSV-1	VZV	mCMV	EBV	KSHV	HSV-1	VZV	mCMV	EBV	KSHV	
1	HSV-1	UL48	10	-	-	-	UL19	40	M86	BcLF1	25	0	0	-	-	-	0
2	HSV-1	UL48	10	-	-	-	UL47	11	-	-	-	1	0	-	-	-	1
3	HSV-1	UL48	10	-	-	-	UL46	12	-	-	-	0	0	-	-	-	0
4	HSV-1	UL48	10	-	-	-	UL22	37	M75	BXLF2	22	0	0	-	-	-	0
5	HSV-1	UL49	9	-	-	-	UL48	10	-	-	-	0	0	-	-	-	0
6	HSV-1	UL49	9	-	-	-	US6	-	-	-	-	0	-	-	-	-	0
7	HSV-1	US11	-	-	-	-	US11	-	-	-	-	1	-	-	-	-	1
8	HSV-1	UL49	9	-	-	-	US9	65	-	-	-	0	0	-	-	-	0
9	HSV-1	UL49	9	-	-	-	UL46	12	-	-	-	0	0	-	-	-	0
10	HSV-1	UL49	9	-	-	-	UL49	9	-	-	-	1	0	-	-	-	1
11	HSV-1	UL46	12	-	-	-	UL18	41	M85	BDLF1	26	0	0	-	-	-	0
12	HSV-1	RL2	61	-	-	-	RL2	61	-	-	-	0	1	-	-	-	1
13	HSV-1	RL2	61	-	-	-	RS1	62	-	-	-	0	1	-	-	-	1
14	HSV-1	UL19	40	M86	BcLF1	25	UL16	44	M94	BGLF2	33	0	0	0	0	0	0
15	HSV-1	UL19	40	M86	BcLF1	25	UL21	38	M88	BTRF1	23	0	0	0	0	0	0
16	HSV-1	UL13	47	M97	BGLF4	36	US8	68	-	-	-	0	0	-	-	-	0
17	HSV-1	UL9	51	-	-	-	UL8	52	M102	BBLF2/BBLF3	40/41	0	0	-	-	-	0
18	HSV-1	UL15	42/45	M89	BGRF1/BDRF1	29a/29b	UL6	54	M104	BBRF1	43	0	0	0	0	0	0
19	HSV-1	UL16	44	M94	BGLF2	33	UL11	49	M99	BBLF1	38	1	0	1	1	0	3
20	HSV-1	UL46	12	-	-	-	UL21	38	M88	BTRF1	23	0	0	-	-	-	0
21	HSV-1	UL46	12	-	-	-	US3	66	-	-	-	0	0	-	-	-	0
22	HSV-1	UL46	12	-	-	-	UL19	40	M86	BcLF1	25	0	0	-	-	-	0
23	HSV-1	UL46	12	-	-	-	UL25	34	M77	BVRF1	19	0	0	-	-	-	0
24	HSV-1	UL25	34	M77	BVRF1	19	UL19	40	M86	BcLF1	25	0	0	0	0	0	0
25	HSV-1	UL26.5	33.5	M80.5	BdRF1	17.5	UL6	54	M104	BBRF1	43	0	0	0	0	0	0
26	HSV-1	UL46	12	-	-	-	US10	64	-	-	-	0	0	-	-	-	0
27	HSV-1	UL46	12	-	-	-	UL10	50	M100	BBRF3	39	0	0	-	-	-	0
28	HSV-1	UL56	0	-	-	-	UL4	56	-	-	-	0	0	-	-	-	0
29	HSV-1	UL35	23	M48.2	BFRF3	65	UL48	10	-	-	-	0	0	-	-	-	0
30	HSV-1	UL35	23	M48.2	BFRF3	65	UL51	7	M71	BSRF1	55	0	0	0	0	0	0

	Protein 1						Protein 2					Confirmed interactions					# species
	Species	HSV-1	VZV	mCMV	EBV	KSHV	HSV-1	VZV	mCMV	EBV	KSHV	HSV-1	VZV	mCMV	EBV	KSHV	
31	HSV-1	UL35	23	M48.2	BFRF3	65	US3	66	-	-	-	0	0	-	-	-	0
32	HSV-1	UL35	23	M48.2	BFRF3	65	UL14	46	M95	BGLF3	34	0	0	0	0	0	0
33	HSV-1	UL35	23	M48.2	BFRF3	65	UL16	44	M94	BGLF2	33	0	0	0	0	0	0
34	HSV-1	UL35	23	M48.2	BFRF3	65	UL21	38	M88	BTRF1	23	0	0	0	0	0	0
35	HSV-1	UL30	28	M54	BALF5	9	UL8	52	M102	BBLF2/BBLF3	40/41	0	0	0	0	1	1
36	HSV-1	UL27	31	M55	BALF4	8	UL27	31	M55	BALF4	8	0	0	0	0	0	0
37	HSV-1	UL27	31	M55	BALF4	8	UL48	10	-	-	-	0	0	-	-	-	0
38	HSV-1	UL34	24	M50	BFRF1	67	UL19	40	M86	BcLF1	25	0	0	0	0	0	0
39	HSV-1	UL34	24	M50	BFRF1	67	US3	66	-	-	-	0	0	-	-	-	0
40	HSV-1	UL33	25	M51	BFRF4	67.5	UL28	30	M56	BALF3	7	1	1	0	1	0	3
41	HSV-1	UL38	20	M46	BORF1	62	UL37	21	M47	BOLF1	63	0	0	0	0	0	0
42	HSV-1	UL38	20	M46	BORF1	62	UL46	12	-	-	-	0	0	-	-	-	0
43	HSV-1	UL37	21	M47	BOLF1	63	UL37	21	M47	BOLF1	63	0	0	0	0	0	0
44	HSV-1	UL40	18	-	BaRF1	60	UL39	19	M45	BORF2	61	0	1	-	0	1	2
45	HSV-1	UL38	20	M46	BORF1	62	UL25	34	M77	BVRF1	19	0	0	0	0	0	0
46	HSV-1	UL38	20	M46	BORF1	62	UL35	23	M48.2	BFRF3	65	0	0	0	0	0	0
47	HSV-1	UL35	23	M48.2	BFRF3	65	UL18	41	M85	BDLF1	26	0	0	0	0	0	0
48	HSV-1	UL35	23	M48.2	BFRF3	65	UL25	34	M77	BVRF1	19	0	0	0	0	0	0
49	HSV-1	UL35	23	M48.2	BFRF3	65	UL11	49	M99	BBLF1	38	0	0	0	0	0	0
50	HSV-1	UL37	21	M47	BOLF1	63	UL35	23	M48.2	BFRF3	65	0	1	0	0	1	2
51	HSV-1	UL37	21	M47	BOLF1	63	UL46	12	-	-	-	0	0	-	-	-	0
52	HSV-1	UL29	29	M57	BALF2	6	UL9	51	-	-	-	0	0	-	-	-	0
53	HSV-1	UL29	29	M57	BALF2	6	UL12	48	M98	BGLF5	37	0	0	0	0	0	0
54	HSV-1	UL29	29	M57	BALF2	6	UL54	4	M69	BSLF2/BMLF1	57	0	0	0	0	0	0
55	HSV-1	UL29	29	M57	BALF2	6	UL8	52	M102	BBLF2/BBLF3	40/41	0	0	0	0	0	0
56	HSV-1	UL36	22	M48	BPLF1	64	UL48	10	-	-	-	1	0	-	-	-	1
57	HSV-1	UL28	30	M56	BALF3	7	UL6	54	M104	BBRF1	43	0	0	0	0	0	0
58	HSV-1, KSHV	UL37	21	M47	BOLF1	63	UL36	22	M48	BPLF1	64	0	1	0	1	0	2
59	HSV-1, VZV	UL54	4	M69	BSLF2/BMLF1	57	RS1	62	-	-	-	0	1	-	-	-	1
60	VZV	UL49	9	-	-	-	RS1	62	-	-	-	0	0	-	-	-	0
61	VZV	UL22	37	M75	BXLF2	22	US8	68	-	-	-	0	0	-	-	-	0
62	VZV	US8	68	-	-	-	US8	68	-	-	-	0	0	-	-	-	0

	Protein 1						Protein 2					Confirmed interactions					# species
	Species	HSV-1	VZV	mCMV	EBV	KSHV	HSV-1	VZV	mCMV	EBV	KSHV	HSV-1	VZV	mCMV	EBV	KSHV	
63	VZV	US7	67	-	-	-	US8	68	-	-	-	0	0	-	-	-	0
64	VZV	RS1	62	-	-	-	US3	66	-	-	-	0	0	-	-	-	0
65	VZV	RS1	62	-	-	-	US1	63	-	-	-	0	0	-	-	-	0
66	HCMV	UL26	33	M80	BVRF2	17	UL26	33	M80	BVRF2	17	0	1	1	0	0	2
67	HCMV	-	-	M35	-	-	-	-	M82	-	-	-	-	0	-	-	0
68	HCMV	-	-	M83	-	-	-	-	M84	-	-	-	-	0	-	-	0
69	HCMV	UL13	47	M97	BGLF4	36	-	-	M83	-	-	-	-	0	-	-	0
70	mCMV	-	-	M142	-	-	-	-	M143	-	-	-	-	1	-	-	1
71	HCMV	UL42	16	M44	BMRF1	59	UL13	47	M97	BGLF4	36	0	0	0	0	0	0
72	mCMV	-	-	M139	-	-	-	-	M141	-	-	-	-	0	-	-	0
73	HCMV	UL42	16	M44	BMRF1	59	-	-	M84	-	-	-	-	0	-	-	0
74	HCMV	UL42	16	M44	BMRF1	59	UL28	30	M56	BALF3	7	0	0	0	0	0	0
75	HCMV	-	-	M84	-	-	-	-	M84	-	-	-	-	0	-	-	0
76	HCMV	UL42	16	M44	BMRF1	59	UL2	59	M114	BKRF3	46	0	1	0	0	0	1
77	HCMV, EBV	UL49A	9a	M73	BLRF1	53	UL10	50	M100	BBRF3	39	1	0	0	0	0	1
78	mCMV, EBV, HSV-1	UL34	24	M50	BFRF1	67	UL31	27	M53	BFLF2	69	1	1	1	1	0	4
79	HCMV, EBV, HSV-1	UL52	6	M70	BSLF1	56	UL5	55	M105	BBLF4	44	0	0	0	0	0	0
80	HCMV, EBV, KSHV	UL42	16	M44	BMRF1	59	UL42	16	M44	BMRF1	59	0	1	0	0	0	1
81	HCMV, HSV-1	UL52	6	M70	BSLF1	56	UL8	52	M102	BBLF2/BBLF3	40/41	0	0	0	0	0	0
82	HCMV, HSV-1	UL38	20	M46	BORF1	62	UL18	41	M85	BDLF1	26	1	0	0	0	0	1
83	HCMV, HSV-1	UL26.5	33.5	M80.5	BdRF1	17.5	UL26.5	33.5	M80.5	BdRF1	17.5	0	0	0	0	0	0
84	HCMV, HSV-1	UL18	41	M85	BDLF1	26	UL18	41	M85	BDLF1	26	0	0	1	0	0	1
85	HCMV, HSV-1	UL15	42/45	M89	BGRF1/BDRF1	29a/29b	UL15	42/45	M89	BGRF1/BDRF1	29a/29b	0	0	0	1	0	1
86	HCMV, HSV-1	UL26	33	M80	BVRF2	17	UL19	40	M86	BcLF1	25	0	0	0	0	0	0
87	HCMV, HSV-1	UL26	33	M80	BVRF2	17	UL26.5	33.5	M80.5	BdRF1	17.5	0	1	0	0	0	1
88	HCMV, HSV-1	UL8	52	M102	BBLF2/BBLF3	40/41	UL5	55	M105	BBLF4	44	0	0	0	0	0	0
89	HCMV, HSV-1	UL26.5	33.5	M80.5	BdRF1	17.5	UL19	40	M86	BcLF1	25	0	0	0	0	0	0
90	HCMV, HSV-1,	UL42	16	M44	BMRF1	59	UL30	28	M54	BALF5	9	0	0	0	0	0	0

	Protein 1						Protein 2					Confirmed interactions					# species
	**Species**	**HSV-1**	**VZV**	**mCMV**	**EBV**	**KSHV**	**HSV-1**	**VZV**	**mCMV**	**EBV**	**KSHV**	**HSV-1**	**VZV**	**mCMV**	**EBV**	**KSHV**	
	KSHV																
91	HCMV, HSV-1, VZV	UL28	30	M56	BALF3	7	UL15	42/45	M89	BGRF1/BDRF1	29a/29b	0	0	0	1	0	1
92	HCMV, HSV-1, VZV	UL22	37	M75	BXLF2	22	UL1	60	M115	BKRF2	47	0	0	0	0	0	0
93	EBV	UL22	37	M75	BXLF2	22	-	-	-	BZLF2	-	-	-	-	0	-	0
94	EBV	UL24	35	M76	BXRF1	20	-	-	-	BILF1	-	-	-	-	0	-	0
95	EBV	UL26.5	33.5	M80.5	BdRF1	17.5	-	-	-	BALF1	-	-	-	-	0	-	0
96	EBV	-	-	-	LF2	11	-	-	-	LF2	11	-	-	-	1	0	1
97	EBV	UL23	36	-	BXLF1	21	UL13	47	M97	BGLF4	36	0	0	-	0	0	0
98	EBV	UL23	36	-	BXLF1	21	-	-	-	EBNA3C	-	-	-	-	0	-	0
99	EBV	UL23	36	-	BXLF1	21	-	-	-	BILF1	-	-	-	-	0	-	0
100	EBV	UL19	40	M86	BcLF1	25	-	-	-	LMP1	-	-	-	-	1	-	1
101	EBV	UL21	38	M88	BTRF1	23	-	-	-	BZLF2	-	-	-	-	0	-	0
102	EBV	UL11	49	M99	BBLF1	38	-	-	-	BLLF1	-	-	-	-	0	-	0
103	EBV	-	-	-	BDLF2	27	-	-	-	BYRF1/EBNA2	-	-	-	-	0	-	0
104	EBV	UL18	41	M85	BDLF1	26	-	-	-	BNLF2b	-	-	-	-	0	-	0
105	EBV	UL52	6	M70	BSLF1	56	UL16	44	M94	BGLF2	33	0	0	0	0	0	0
106	EBV	UL30	28	M54	BALF5	9	UL5	55	M105	BBLF4	44	0	0	0	0	0	0
107	EBV	UL30	28	M54	BALF5	9	UL52	6	M70	BSLF1	56	0	0	0	0	0	0
108	EBV	-	-	-	BZLF1	K08	UL5	55	M105	BBLF4	44	-	-	-	0	0	0
109	EBV	UL51	7	M71	BSRF1	55	UL6	54	M104	BBRF1	43	0	0	0	0	0	0
110	EBV	UL49A	9a	M73	BLRF1	53	-	-	-	LMP1	-	-	-	-	0	-	0
111	EBV	-	-	-	BRLF1	50	-	-	-	LF2	11	-	-	-	0	0	0
112	EBV	-	-	-	BRLF1	50	UL23	36	-	BXLF1	21	-	-	-	0	0	0
113	EBV	-	-	-	BRRF2	48	-	-	-	LMP2A/LMP2B	K15	-	-	-	0	0	0
114	EBV	UL27	31	M55	BALF4	8	-	-	-	EBNA3B	-	-	-	-	0	-	0
115	EBV	-	-	-	BZLF1	K08	-	-	-	BZLF1	K08	-	-	-	0	0	0
116	EBV	UL18	41	M85	BDLF1	26	-	-	-	EBNA3B	-	-	-	-	0	-	0
117	EBV	-	-	-	BZLF1	K08	-	-	-	BYRF1/EBNA2	-	-	-	-	0	-	0
118	EBV	-	-	-	BKRF1/EBNA	-	-	-	-	EBNA3C	-	-	-	-	0	-	0

	Protein 1						Protein 2					Confirmed interactions					# species
	**Species**	**HSV-1**	**VZV**	**mCMV**	**EBV**	**KSHV**	**HSV-1**	**VZV**	**mCMV**	**EBV**	**KSHV**	**HSV-1**	**VZV**	**mCMV**	**EBV**	**KSHV**	
					1												
119	EBV	UL40	18	-	BaRF1	60	-	-	-	BILF1	-	-	-	-	0	-	0
120	EBV	UL40	18	-	BaRF1	60	UL49A	9a	M73	BLRF1	53	0	0	-	0	0	0
121	EBV	UL40	18	-	BaRF1	60	-	-	-	BNRF1	75	-	-	-	0	0	0
122	EBV	-	-	-	BLLF2	-	-	-	-	EBNA3B	-	-	-	-	0	-	0
123	EBV	-	-	-	EBNA3A	-	-	-	-	EBNA3C	-	-	-	-	0	-	0
124	EBV	-	-	-	BLLF2	-	-	-	-	LMP1	-	-	-	-	0	-	0
125	EBV	UL40	18	-	BaRF1	60	UL36	22	M48	BPLF1	64	0	0	-	0	0	0
126	EBV	-	-	-	BYRF1/EBNA2	-	-	-	-	BYRF1/EBNA2	-	-	-	-	0	-	0
127	EBV	-	-	-	BYRF1/EBNA2	-	-	-	-	EBNA3A	-	-	-	-	0	-	0
128	EBV	-	-	-	EBNA3A	-	-	-	-	EBNA3A	-	-	-	-	0	-	0
129	EBV	UL40	18	-	BaRF1	60	UL40	18	-	BaRF1	60	1	0	-	0	1	2
130	EBV	-	-	-	BNLF2a	-	-	-	-	BNLF2a	-	-	-	-	1	-	1
131	EBV	UL1	60	M115	BKRF2	47	-	-	-	LMP1	-	-	-	-	0	-	0
132	EBV	UL42	16	M44	BMRF1	59	-	-	-	EBNA3B	-	-	-	-	0	-	0
133	EBV	UL42	16	M44	BMRF1	59	UL12	48	M98	BGLF5	37	0	0	0	0	0	0
134	EBV	-	-	-	EBNA-LP	-	-	-	-	EBNA-LP	-	-	-	-	0	-	0
135	EBV	UL42	16	M44	BMRF1	59	-	-	-	BZLF1	K08	-	-	-	0	0	0
136	EBV	-	-	-	BILF1	-	-	-	-	BKRF1/EBNA1	-	-	-	-	0	-	0
137	EBV	-	-	-	BKRF1/EBNA1	-	-	-	-	BKRF1/EBNA1	-	-	-	-	0	-	0
138	EBV	-	-	-	BYRF1/EBNA2	-	-	-	-	EBNA-LP	-	-	-	-	0	-	0
139	EBV	-	-	-	BCRF1	-	-	-	-	BCRF1	-	-	-	-	0	-	0
140	EBV, HSV-1, KSHV	UL54	4	M69	BSLF2/BMLF1	57	UL54	4	M69	BSLF2/BMLF1	57	1	0	1	0	1	3
141	EBV, HSV-1, KSHV	UL35	23	M48.2	BFRF3	65	UL19	40	M86	BcLF1	25	0	0	1	0	1	2
142	EBV, KSHV	-	-	-	BRLF1	50	-	-	-	BRLF1	50	-	-	-	0	0	0
143	EBV, KSHV	UL36	22	M48	BPLF1	64	UL36	22	M48	BPLF1	64	0	0	0	1	0	1
144	EBV, KSHV	-	-	-	BLRF2	52	-	-	-	BNRF1	75	-	-	-	1	0	1
145	KSHV	UL36	22	M48	BPLF1	64	UL23	36	-	BXLF1	21	0	0	-	0	0	0

	Protein 1						Protein 2					Confirmed interactions					# species
	Species	HSV-1	VZV	mCMV	EBV	KSHV	HSV-1	VZV	mCMV	EBV	KSHV	HSV-1	VZV	mCMV	EBV	KSHV	
146	KSHV	UL37	21	M47	BOLF1	63	UL23	36	-	BXLF1	21	0	0	-	0	0	0
147	KSHV	UL36	22	M48	BPLF1	64	-	-	-	BKRF4	45	-	-	-	0	0	0
148	KSHV	UL37	21	M47	BOLF1	63	-	-	-	BKRF4	45	-	-	-	0	0	0
149	KSHV	UL37	21	M47	BOLF1	63	UL49A	9a	M73	BLRF1	53	0	0	0	0	0	0
150	KSHV	UL37	21	M47	BOLF1	63	UL1	60	M115	BKRF2	47	0	0	0	0	0	0
151	KSHV	UL49A	9a	M73	BLRF1	53	-	-	-	BLRF2	52	-	-	-	0	0	0
152	KSHV	UL18	41	M85	BDLF1	26	-	-	-	BKRF4	45	-	-	-	0	0	0
153	KSHV	UL36	22	M48	BPLF1	64	UL18	41	M85	BDLF1	26	0	1	0	0	0	1
154	KSHV	-	-	-	LF2	11	-	-	-	BKRF4	45	-	-	-	0	0	0
155	KSHV	-	-	-	BDLF3	28	UL16	44	M94	BGLF2	33	-	-	-	0	0	0
156	KSHV	UL36	22	M48	BPLF1	64	UL19	40	M86	BcLF1	25	0	0	0	0	0	0
157	KSHV	-	-	-	LF2	11	UL1	60	M115	BKRF2	47	-	-	-	0	0	0
158	KSHV	-	-	-	BDLF2	27	UL1	60	M115	BKRF2	47	-	-	-	1	0	1
159	KSHV	-	-	-	BDLF2	27	-	-	-	BKRF4	45	-	-	-	0	0	0
160	KSHV	UL36	22	M48	BPLF1	64	-	-	-	BLRF2	52	-	-	-	0	0	0
161	KSHV	-	-	-	BDLF2	27	UL10	50	M100	BBRF3	39	-	-	-	1	0	1
162	KSHV	UL49A	9a	M73	BLRF1	53	-	-	-	BKRF4	45	-	-	-	0	0	0
163	KSHV	UL38	20	M46	BORF1	62	-	-	-	BKRF4	45	-	-	-	0	0	0
164	KSHV	UL54	4	M69	BSLF2/BMLF1	57	-	-	-	BRLF1	50	-	-	-	0	1	1
165	KSHV	UL38	20	M46	BORF1	62	-	-	-	BNRF1	75	-	-	-	0	0	0
166	KSHV	UL38	20	M46	BORF1	62	UL36	22	M48	BPLF1	64	0	0	0	0	0	0
167	KSHV	UL49A	9a	M73	BLRF1	53	UL23	36	-	BXLF1	21	0	0	-	0	0	0
168	KSHV	UL49A	9a	M73	BLRF1	53	-	-	-	LF2	11	-	-	-	0	0	0
169	KSHV	-	-	-	-	K12	-	-	-	-	K12	-	-	-	-	1	1
170	KSHV	UL38	20	M46	BORF1	62	UL23	36	-	BXLF1	21	0	0	-	0	0	0
171	KSHV	UL49A	9a	M73	BLRF1	53	UL16	44	M94	BGLF2	33	0	0	0	0	0	0
172	KSHV	UL49A	9a	M73	BLRF1	53	-	-	-	BDLF2	27	-	-	-	1	0	1
173	KSHV	UL16	44	M94	BGLF2	33	UL10	50	M100	BBRF3	39	0	0	0	0	0	0

	Protein 1						Protein 2					Confirmed interactions					# species
	Species	HSV-1	VZV	mCMV	EBV	KSHV	HSV-1	VZV	mCMV	EBV	KSHV	HSV-1	VZV	mCMV	EBV	KSHV	
174	KSHV	UL16	44	M94	BGLF2	33	-	-	-	BKRF4	45	-	-	-	0	0	0
175	KSHV	-	-	-	BKRF4	45	-	-	-	BNRF1	75	-	-	-	0	0	0
176	KSHV	-	-	-	BKRF4	45	UL1	60	M115	BKRF2	47	-	-	-	0	0	0
177	KSHV	UL10	50	M100	BBRF3	39	-	-	-	BKRF4	45	-	-	-	0	0	0
178	KSHV	UL10	50	M100	BBRF3	39	-	-	-	BNRF1	75	-	-	-	0	0	0
179	KSHV	UL22	37	M75	BXLF2	22	UL16	44	M94	BGLF2	33	0	0	0	0	0	0
180	KSHV	UL22	37	M75	BXLF2	22	-	-	-	BDLF2	27	-	-	-	0	0	0
181	KSHV	UL22	37	M75	BXLF2	22	UL23	36	-	BXLF1	21	0	0	-	0	0	0
182	KSHV	UL36	22	M48	BPLF1	64	UL1	60	M115	BKRF2	47	0	0	0	0	0	0
183	KSHV	UL36	22	M48	BPLF1	64	-	-	-	BDLF3	28	-	-	-	0	0	0
184	KSHV	UL36	22	M48	BPLF1	64	UL10	50	M100	BBRF3	39	0	0	0	1	0	1
185	KSHV	UL36	22	M48	BPLF1	64	UL22	37	M75	BXLF2	22	0	0	0	0	0	0
186	KSHV	UL22	37	M75	BXLF2	22	-	-	-	BKRF4	45	-	-	-	0	0	0
187	KSHV	-	-	-	BLRF2	52	-	-	-	BKRF4	45	-	-	-	0	0	0
188	KSHV	-	-	-	BLRF2	52	UL10	50	M100	BBRF3	39	-	-	-	0	0	0
189	KSHV	-	-	-	BLRF2	52	UL1	60	M115	BKRF2	47	-	-	-	0	0	0
190	KSHV	UL35	23	M48.2	BFRF3	65	-	-	-	BKRF4	45	-	-	-	0	0	0
191	KSHV	UL49A	9a	M73	BLRF1	53	-	-	-	BNRF1	75	-	-	-	0	0	0
192	KSHV	-	-	-	BRLF1	50	-	-	-	-	73	-	-	-	-	0	0
193	KSHV	-	-	-	BLRF2	52	UL16	44	M94	BGLF2	33	-	-	-	0	0	0
194	KSHV	UL23	36	-	BXLF1	21	-	-	-	BKRF4	45	-	-	-	0	0	0
195	KSHV	UL23	36	-	BXLF1	21	UL1	60	M115	BKRF2	47	0	0	-	0	0	0
196	KSHV	UL23	36	-	BXLF1	21	-	-	-	BNRF1	75	-	-	-	0	0	0
197	KSHV	UL36	22	M48	BPLF1	64	-	-	-	LF2	11	-	-	-	0	0	0
198	KSHV	UL36	22	M48	BPLF1	64	UL16	44	M94	BGLF2	33	0	0	1	0	0	1
199	KSHV	UL23	36	-	BXLF1	21	UL19	40	M86	BcLF1	25	0	0	-	0	0	0
200	KSHV	UL23	36	-	BXLF1	21	-	-	-	LF2	11	-	-	-	0	0	0
201	KSHV	-	-	-	BLRF2	52	UL18	41	M85	BDLF1	26	-	-	-	0	0	0
202	KSHV	UL36	22	M48	BPLF1	64	-	-	-	BNRF1	75	-	-	-	0	0	0
203	KSHV	UL23	36	-	BXLF1	21	UL23	36	-	BXLF1	21	0	1	-	0	0	1
