# Supplementary material for: Evolutionarily Conserved Herpesviral Protein Interaction Networks
Source: PLoS Pathog. 2009 Sep 4;5(9):e1000570. doi: 10.1371/journal.ppat.1000570 (PMC2731838; doi:10.1371/journal.ppat.1000570)
Supplement: Table S4 — Network parameters in herpesviruses. Topological parameters describing the network structure for all five viral interactomes. Self-interactions were not included for the computation of average clustering coefficients, characteristic path length and network diameter as well as enrichment values. For the enrichment over ES values, rewiring was performed 104 times and clustering coefficients of the rewired networks were averaged. The expected clustering coefficient of the ER networks was computed as with K the total number of edges in the network and N the total number of nodes. (0.07 MB PDF) [file ppat.1000570.s018.pdf]

Table S4: Network parameters in herpesviruses.

Network Parameters	HSV-1	VZV	mCMV	EBV	KSHV
**# Protein**	48	57	111	61	50
**# Interactions**	111	173	406	218	123
**# Interactions (without self-interactions)**	100	160	393	208	115
**Average degree**	4.63	6.07	7.32	7.15	4.92
**Average degree (without self-interactions)**	4.17	5.61	7.08	6.82	4.60
**Power coefficient**	0.99	0.79	0.94	0.74	0.82
**R2**	0.84	0.74	0.77	0.66	0.70
**Characteristic path length**	2.79	2.34	2.84	2.44	2.84
**Diameter**	6	5	7	5	7
**Clustering coefficient**	0.25	0.39	0.24	0.40	0.15
**Enrichment over ER**	2.52	3.61	3.66	3.36	1.45
**Enrichment over ES**	0.80	1.00	1.24	1.14	0.69
**% published interactions (published only for this virus)**	8/111=7.2%	0/173=0.0%	4/406=1.0%	1/218=0.5%	4/123=3.3%
**% published interactions (published for this virus or homologs in other viruses)**	9/111=8.1%	9/173=5.2%	8/406=2.0%	5/218=2.3%	7/123=5.7%
**% published interactions found (published only for this virus)**	8/59=13.6%	0/5=0.0%	4/20=20.0%	1/25=4.0%	4/11=36.4%
**% published interactions found (published for this virus or homologs in other viruses)**	9/71=12.7%	9/67=13.4%	8/51=15.7%	5/58=8.6%	7/54=13.0%
