# Supplementary material for: Evolutionarily Conserved Herpesviral Protein Interaction Networks
Source: PLoS Pathog. 2009 Sep 4;5(9):e1000570. doi: 10.1371/journal.ppat.1000570 (PMC2731838; doi:10.1371/journal.ppat.1000570)
Supplement: Table S5 — Ortholog proteins in five herpesvirus species. List of orthologous genes conserved between the five herpesviral species based on Davison et al [31]. (0.01 MB PDF) [file ppat.1000570.s019.pdf]

Table S5: Ortholog proteins in five herpesvirus species.

HSV-1	VZV	mCMV	EBV	KSHV
UL56	0	-	-	-
UL55	3	-	-	-
UL42	16	M44	BMRF1	59
UL41	17	-	-	-
UL40	18	-	BaRF1	60
UL39	19	M45	BORF2	61
UL38	20	M46	BORF1	62
UL37	21	M47	BOLF1	63
UL36	22	M48	BPLF1	64
UL35	23	M48.2	BFRF3	65
-	-	M49	BFRF2	66
UL34	24	M50	BFRF1	67
UL33	25	M51	BFRF4	67.5
UL32	26	M52	BFLF1	68
UL31	27	M53	BFLF2	69
UL30	28	M54	BALF5	9
UL27	31	M55	BALF4	8
UL28	30	M56	BALF3	7
UL29	29	M57	BALF2	6
-	-	-	BMRF2	58
UL54	4	M69	BSLF2	57
-	-	-	BMLF1	-
UL53	5	-	-	-
UL52	6	M70	BSLF1	56
UL51	7	M71	BSRF1	55
UL50	8	M72	BLLF3	54
UL49A	9a	M73	BLRF1	53
-	-	-	BLRF2	52
UL49	9	-	-	-
UL48	10	-	-	-
UL47	11	-	-	-
UL46	12	-	-	-
UL44	14	-	-	-
UL43	15	-	-	-
-	-	-	BZLF1	K08
-	-	-	BRLF1	50
-	-	-	BRRF1	49
-	-	-	BRRF2	48
UL22	37	M75	BXLF2	22
UL23	36	-	BXLF1	21
UL24	35	M76	BXRF1	20
UL25	34	M77	BVRF1	19
-	-	M79	BVLF1.5	18
UL26	33	M80	BVRF2	17

HSV-1	VZV	mCMV	EBV	KSHV
UL26.5	33.5	M80.5	BdRF1	17.5
-	-	-	LF2	11
-	-	-	BDLF3	28
-	-	-	BDLF2	27
UL18	41	M85	BDLF1	26
UL19	40	M86	BcLF1	25
-	-	M87	BcRF1	24
UL20	39	-	-	-
UL21	38	M88	BTRF1	23
UL15	42	M89	BGRF1	29a
-	45	-	BDRF1	29b
-	-	M91	BDLF3.5	30
-	-	M92	BDLF4	31
UL17	43	M93	BGLF1	32
UL16	44	M94	BGLF2	33
UL14	46	M95	BGLF3	34
-	-	M96	BGLF3.5	35
UL13	47	M97	BGLF4	36
UL12	48	M98	BGLF5	37
UL11	49	M99	BBLF1	38
UL10	50	M100	BBRF3	39
UL9	51	-	-	-
UL8	52	M102	BBLF2	40
-	-	-	BBLF3	41
UL7	53	M103	BBRF2	42
UL6	54	M104	BBRF1	43
UL5	55	M105	BBLF4	44
-	-	-	BKRF4	45
UL4	56	-	-	-
UL3	58	-	-	-
UL2	59	M114	BKRF3	46
UL1	60	M115	BKRF2	47
RL2	61	-	-	-
RS1	62	-	-	-
US1	63	-	-	-
US3	66	-	-	-
-	-	-	BNRF1	75
-	-	-	LMP2A	K15
-	-	-	LMP2B	-
US7	67	-	-	-
US8	68	-	-	-
US9	65	-	-	-
US10	64	-	-	-
