# Supplementary material for: Evolutionarily Conserved Herpesviral Protein Interaction Networks
Source: PLoS Pathog. 2009 Sep 4;5(9):e1000570. doi: 10.1371/journal.ppat.1000570 (PMC2731838; doi:10.1371/journal.ppat.1000570)
Supplement: Table S6 — Average sequence similarity of core proteins between five herpesvirus species. Average sequence similarity for each of the core herpesviral proteins. The average similarities were calculated as specified in material and methods. (0.02 MB PDF) [file ppat.1000570.s020.pdf]

**Table S6: Average sequence similarity of core proteins between five herpesvirus species.**

| Protein | Avg. sequence similarity |
|---|---|
| KSHV_59/EBV_BMRF1/mCMV_M44/HSV-1_UL42/VZV_16 | 23.553 |
| KSHV_61/EBV_BORF2/mCMV_M45/HSV-1_UL39/VZV_19 | 34.257 |
| KSHV_62/EBV_BORF1/mCMV_M46/HSV-1_UL38/VZV_20 | 29.161 |
| KSHV_63/EBV_BOLF1/mCMV_M47/HSV-1_UL37/VZV_21 | 25.133 |
| KSHV_64/EBV_BPLF1/mCMV_M48/HSV-1_UL36/VZV_22 | 30.714 |
| KSHV_65/EBV_BFRF3/mCMV_M48.2/HSV-1_UL35/VZV_23 | 19.156 |
| KSHV_67/EBV_BFRF1/mCMV_M50/HSV-1_UL34/VZV_24 | 32.732 |
| KSHV_67.5/EBV_BFRF4/mCMV_M51/HSV-1_UL33/VZV_25 | 26.298 |
| KSHV_68/EBV_BFLF1/mCMV_M52/HSV-1_UL32/VZV_26 | 37.297 |
| KSHV_69/EBV_BFLF2/mCMV_M53/HSV-1_UL31/VZV_27 | 34.515 |
| KSHV_9/EBV_BALF5/mCMV_M54/HSV-1_UL30/VZV_28 | 51.404 |
| KSHV_8/EBV_BALF4/mCMV_M55/HSV-1_UL27/VZV_31 | 46.551 |
| KSHV_7/EBV_BALF3/mCMV_M56/HSV-1_UL28/VZV_30 | 40.449 |
| KSHV_6/EBV_BALF2/mCMV_M57/HSV-1_UL29/VZV_29 | 42.907 |
| KSHV_57/EBV_BSLF2/mCMV_M69/HSV-1_UL54/VZV_4/EBV_BMLF1 | 22.837 |
| KSHV_56/EBV_BSLF1/mCMV_M70/HSV-1_UL52/VZV_6 | 38.204 |
| KSHV_55/EBV_BSRF1/mCMV_M71/HSV-1_UL51/VZV_7 | 33.883 |
| KSHV_54/EBV_BLLF3/mCMV_M72/HSV-1_UL50/VZV_8 | 29.874 |
| KSHV_53/EBV_BLRF1/mCMV_M73/HSV-1_UL49A/VZV_9a | 29.91 |
| KSHV_22/EBV_BXLF2/mCMV_M75/HSV-1_UL22/VZV_37 | 32.864 |
| KSHV_20/EBV_BXRF1/mCMV_M76/HSV-1_UL24/VZV_35 | 34.643 |
| KSHV_19/EBV_BVRF1/mCMV_M77/HSV-1_UL25/VZV_34 | 39.708 |
| KSHV_17/EBV_BVRF2/mCMV_M80/HSV-1_UL26/VZV_33 | 36.102 |
| KSHV_17.5/EBV_BdRF1/mCMV_M80.5/HSV-1_UL26.5/VZV_33.5 | 30.163 |
| KSHV_26/EBV_BDLF1/mCMV_M85/HSV-1_UL18/VZV_41 | 43.253 |
| KSHV_25/EBV_BcLF1/mCMV_M86/HSV-1_UL19/VZV_40 | 50.092 |
| KSHV_23/EBV_BTRF1/mCMV_M88/HSV-1_UL21/VZV_38 | 26.378 |
| KSHV_29a/EBV_BGRF1/mCMV_M89/HSV-1_UL15/VZV_42/KSHV_29b/EBV_BDRF1/VZV_45 | 48.96 |
| KSHV_32/EBV_BGLF1/mCMV_M93/HSV-1_UL17/VZV_43 | 28.871 |
| KSHV_33/EBV_BGLF2/mCMV_M94/HSV-1_UL16/VZV_44 | 33.507 |
| KSHV_34/EBV_BGLF3/mCMV_M95/HSV-1_UL14/VZV_46 | 24.107 |
| KSHV_36/EBV_BGLF4/mCMV_M97/HSV-1_UL13/VZV_47 | 29.943 |
| KSHV_37/EBV_BGLF5/mCMV_M98/HSV-1_UL12/VZV_48 | 36.244 |
| KSHV_38/EBV_BBLF1/mCMV_M99/HSV-1_UL11/VZV_49 | 28.095 |
| KSHV_39/EBV_BBRF3/mCMV_M100/HSV-1_UL10/VZV_50 | 38.423 |
| KSHV_40/EBV_BBLF2/mCMV_M102/HSV-1_UL8/VZV_52/KSHV_41/EBV_BBLF3 | 26.009 |
| KSHV_42/EBV_BBRF2/mCMV_M103/HSV-1_UL7/VZV_53 | 32.26 |
| KSHV_43/EBV_BBRF1/mCMV_M104/HSV-1_UL6/VZV_54 | 41.13 |
| KSHV_44/EBV_BBLF4/mCMV_M105/HSV-1_UL5/VZV_55 | 51.825 |
| KSHV_46/EBV_BKRF3/mCMV_M114/HSV-1_UL2/VZV_59 | 46.313 |
| KSHV_47/EBV_BKRF2/mCMV_M115/HSV-1_UL1/VZV_60 | 13.7 |
