# Supplementary material for: Evolutionarily Conserved Herpesviral Protein Interaction Networks
Source: PLoS Pathog. 2009 Sep 4;5(9):e1000570. doi: 10.1371/journal.ppat.1000570 (PMC2731838; doi:10.1371/journal.ppat.1000570)
Supplement: Table S7 — Protein interactions between herpesvirus core orthologs. List of interactions observed between the 41 core proteins for all five species. The table indicates the number of species in which an interaction was observed, in addition to the specific species in which the interactions were observed. (0.01 MB PDF) [file ppat.1000570.s021.pdf]

Table S7: Protein interactions between herpesvirus core orthologs.

	Protein 1	Protein2	Virus Occurences	HSV-1	VZV	mCMV	EBV	KSHV
1	HSV-1_UL42/VZV_16/mCMV_M44/EBV_BMRF1/KSHV_59	HSV-1_UL33/VZV_25/mCMV_M51/EBV_BFRF4/KSHV_67.5	1	0	0	0	0	1
2	HSV-1_UL42/VZV_16/mCMV_M44/EBV_BMRF1/KSHV_59	HSV-1_UL32/VZV_26/mCMV_M52/EBV_BFLF1/KSHV_68	1	0	0	0	0	1
3	HSV-1_UL42/VZV_16/mCMV_M44/EBV_BMRF1/KSHV_59	HSV-1_UL42/VZV_16/mCMV_M44/EBV_BMRF1/KSHV_59	1	0	1	0	0	0
4	HSV-1_UL42/VZV_16/mCMV_M44/EBV_BMRF1/KSHV_59	HSV-1_UL39/VZV_19/mCMV_M45/EBV_BORF2/KSHV_61	1	0	1	0	0	0
5	HSV-1_UL42/VZV_16/mCMV_M44/EBV_BMRF1/KSHV_59	HSV-1_UL34/VZV_24/mCMV_M50/EBV_BFRF1/KSHV_67	1	0	1	0	0	0
6	HSV-1_UL42/VZV_16/mCMV_M44/EBV_BMRF1/KSHV_59	HSV-1_UL26/VZV_33/mCMV_M80/EBV_BVRF2/KSHV_17	1	0	1	0	0	0
7	HSV-1_UL42/VZV_16/mCMV_M44/EBV_BMRF1/KSHV_59	HSV-1_UL15/VZV_42/VZV_45/mCMV_M89/EBV_BGRF1/EBV_BDRF1/KSHV_29a/KSHV_29b	1	0	1	0	0	0
8	HSV-1_UL42/VZV_16/mCMV_M44/EBV_BMRF1/KSHV_59	HSV-1_UL2/VZV_59/mCMV_M114/EBV_BKRF3/KSHV_46	1	0	1	0	0	0
9	HSV-1_UL42/VZV_16/mCMV_M44/EBV_BMRF1/KSHV_59	HSV-1_UL31/VZV_27/mCMV_M53/EBV_BFLF2/KSHV_69	1	0	1	0	0	0
10	HSV-1_UL39/VZV_19/mCMV_M45/EBV_BORF2/KSHV_61	HSV-1_UL13/VZV_47/mCMV_M97/EBV_BGLF4/KSHV_36	1	0	0	0	0	1
11	HSV-1_UL39/VZV_19/mCMV_M45/EBV_BORF2/KSHV_61	HSV-1_UL54/VZV_4/mCMV_M69/EBV_BSLF2/EBV_BMLF1/KSHV_57	1	0	0	0	0	1
12	HSV-1_UL39/VZV_19/mCMV_M45/EBV_BORF2/KSHV_61	HSV-1_UL39/VZV_19/mCMV_M45/EBV_BORF2/KSHV_61	3	0	1	1	0	1
13	HSV-1_UL39/VZV_19/mCMV_M45/EBV_BORF2/KSHV_61	HSV-1_UL50/VZV_8/mCMV_M72/EBV_BLLF3/KSHV_54	2	0	1	1	0	0
14	HSV-1_UL39/VZV_19/mCMV_M45/EBV_BORF2/KSHV_61	HSV-1_UL33/VZV_25/mCMV_M51/EBV_BFRF4/KSHV_67.5	2	0	1	1	0	0
15	HSV-1_UL39/VZV_19/mCMV_M45/EBV_BORF2/KSHV_61	HSV-1_UL36/VZV_22/mCMV_M48/EBV_BPLF1/KSHV_64	1	0	0	1	0	0
16	HSV-1_UL39/VZV_19/mCMV_M45/EBV_BORF2/KSHV_61	HSV-1_UL17/VZV_43/mCMV_M93/EBV_BGLF1/KSHV_32	1	0	1	0	0	0
17	HSV-1_UL39/VZV_19/mCMV_M45/EBV_BORF2/KSHV_61	HSV-1_UL31/VZV_27/mCMV_M53/EBV_BFLF2/KSHV_69	1	0	1	0	0	0
18	HSV-1_UL39/VZV_19/mCMV_M45/EBV_BORF2/KSHV_61	HSV-1_UL21/VZV_38/mCMV_M88/EBV_BTRF1/KSHV_23	1	0	1	0	0	0
19	HSV-1_UL38/VZV_20/mCMV_M46/EBV_BORF1/KSHV_62	HSV-1_UL50/VZV_8/mCMV_M72/EBV_BLLF3/KSHV_54	1	0	0	0	0	1
20	HSV-1_UL38/VZV_20/mCMV_M46/EBV_BORF1/KSHV_62	HSV-1_UL28/VZV_30/mCMV_M56/EBV_BALF3/KSHV_7	1	0	0	0	1	0
21	HSV-1_UL38/VZV_20/mCMV_M46/EBV_BORF1/KSHV_62	HSV-1_UL27/VZV_31/mCMV_M55/EBV_BALF4/KSHV_8	1	0	0	0	1	0
22	HSV-1_UL38/VZV_20/mCMV_M46/EBV_BORF1/KSHV_62	HSV-1_UL8/VZV_52/mCMV_M102/EBV_BBLF2/EBV_BBLF3/KSHV_40/KSHV_41	1	0	0	0	1	0
23	HSV-1_UL38/VZV_20/mCMV_M46/EBV_BORF1/KSHV_62	HSV-1_UL32/VZV_26/mCMV_M52/EBV_BFLF1/KSHV_68	1	0	0	0	1	0
24	HSV-1_UL38/VZV_20/mCMV_M46/EBV_BORF1/KSHV_62	HSV-1_UL14/VZV_46/mCMV_M95/EBV_BGLF3/KSHV_34	1	1	0	0	0	0
25	HSV-1_UL38/VZV_20/mCMV_M46/EBV_BORF1/KSHV_62	HSV-1_UL18/VZV_41/mCMV_M85/EBV_BDLF1/KSHV_26	1	1	0	0	0	0
26	HSV-1_UL38/VZV_20/mCMV_M46/EBV_BORF1/KSHV_62	HSV-1_UL33/VZV_25/mCMV_M51/EBV_BFRF4/KSHV_67.5	1	1	0	0	0	0
27	HSV-1_UL37/VZV_21/mCMV_M47/EBV_BOLF1/KSHV_63	HSV-1_UL21/VZV_38/mCMV_M88/EBV_BTRF1/KSHV_23	1	0	0	0	0	1
28	HSV-1_UL37/VZV_21/mCMV_M47/EBV_BOLF1/KSHV_63	HSV-1_UL8/VZV_52/mCMV_M102/EBV_BBLF2/EBV_BBLF3/KSHV_40/KSHV_41	1	0	0	0	0	1
29	HSV-1_UL37/VZV_21/mCMV_M47/EBV_BOLF1/KSHV_63	HSV-1_UL35/VZV_23/mCMV_M48.2/EBV_BFRF3/KSHV_65	2	0	1	0	0	1
30	HSV-1_UL37/VZV_21/mCMV_M47/EBV_BOLF1/KSHV_63	HSV-1_UL33/VZV_25/mCMV_M51/EBV_BFRF4/KSHV_67.5	1	0	0	0	0	1
31	HSV-1_UL37/VZV_21/mCMV_M47/EBV_BOLF1/KSHV_63	HSV-1_UL27/VZV_31/mCMV_M55/EBV_BALF4/KSHV_8	1	0	0	0	1	0
32	HSV-1_UL37/VZV_21/mCMV_M47/EBV_BOLF1/KSHV_63	HSV-1_UL36/VZV_22/mCMV_M48/EBV_BPLF1/KSHV_64	2	0	1	0	1	0
33	HSV-1_UL37/VZV_21/mCMV_M47/EBV_BOLF1/KSHV_63	HSV-1_UL2/VZV_59/mCMV_M114/EBV_BKRF3/KSHV_46	1	1	0	0	0	0
34	HSV-1_UL37/VZV_21/mCMV_M47/EBV_BOLF1/KSHV_63	HSV-1_UL26/VZV_33/mCMV_M80/EBV_BVRF2/KSHV_17	1	0	1	0	0	0
35	HSV-1_UL37/VZV_21/mCMV_M47/EBV_BOLF1/KSHV_63	HSV-1_UL26.5/VZV_33.5/mCMV_M80.5/EBV_BdRF1/KSHV_17.5	1	0	1	0	0	0

	Protein 1	Protein2	Virus Occurences	HSV-1	VZV	mCMV	EBV	KSHV
36	HSV-1_UL37/VZV_21/mCMV_M47/EBV_BOLF1/KSHV_63	HSV-1_UL15/VZV_42/VZV_45/mCMV_M89/EBV_BGRF1/EBV_BDRF1/KSHV_29a/KSHV_29b	1	0	1	0	0	0
37	HSV-1_UL37/VZV_21/mCMV_M47/EBV_BOLF1/KSHV_63	HSV-1_UL31/VZV_27/mCMV_M53/EBV_BFLF2/KSHV_69	1	0	1	0	0	0
38	HSV-1_UL36/VZV_22/mCMV_M48/EBV_BPLF1/KSHV_64	HSV-1_UL27/VZV_31/mCMV_M55/EBV_BALF4/KSHV_8	1	0	0	0	1	0
39	HSV-1_UL36/VZV_22/mCMV_M48/EBV_BPLF1/KSHV_64	HSV-1_UL28/VZV_30/mCMV_M56/EBV_BALF3/KSHV_7	1	0	0	0	1	0
40	HSV-1_UL36/VZV_22/mCMV_M48/EBV_BPLF1/KSHV_64	HSV-1_UL10/VZV_50/mCMV_M100/EBV_BBRF3/KSHV_39	1	0	0	0	1	0
41	HSV-1_UL36/VZV_22/mCMV_M48/EBV_BPLF1/KSHV_64	HSV-1_UL15/VZV_42/VZV_45/mCMV_M89/EBV_BGRF1/EBV_BDRF1/KSHV_29a/KSHV_29b	1	0	0	0	1	0
42	HSV-1_UL36/VZV_22/mCMV_M48/EBV_BPLF1/KSHV_64	HSV-1_UL31/VZV_27/mCMV_M53/EBV_BFLF2/KSHV_69	2	0	1	0	1	0
43	HSV-1_UL36/VZV_22/mCMV_M48/EBV_BPLF1/KSHV_64	HSV-1_UL33/VZV_25/mCMV_M51/EBV_BFRF4/KSHV_67.5	2	0	0	1	1	0
44	HSV-1_UL36/VZV_22/mCMV_M48/EBV_BPLF1/KSHV_64	HSV-1_UL36/VZV_22/mCMV_M48/EBV_BPLF1/KSHV_64	1	0	0	0	1	0
45	HSV-1_UL36/VZV_22/mCMV_M48/EBV_BPLF1/KSHV_64	HSV-1_UL21/VZV_38/mCMV_M88/EBV_BTRF1/KSHV_23	1	0	0	0	2	0
46	HSV-1_UL36/VZV_22/mCMV_M48/EBV_BPLF1/KSHV_64	HSV-1_UL25/VZV_34/mCMV_M77/EBV_BVRF1/KSHV_19	2	0	1	1	0	0
47	HSV-1_UL36/VZV_22/mCMV_M48/EBV_BPLF1/KSHV_64	HSV-1_UL16/VZV_44/mCMV_M94/EBV_BGLF2/KSHV_33	1	0	0	1	0	0
48	HSV-1_UL36/VZV_22/mCMV_M48/EBV_BPLF1/KSHV_64	HSV-1_UL49A/VZV_9a/mCMV_M73/EBV_BLRF1/KSHV_53	1	0	1	0	0	0
49	HSV-1_UL36/VZV_22/mCMV_M48/EBV_BPLF1/KSHV_64	HSV-1_UL26/VZV_33/mCMV_M80/EBV_BVRF2/KSHV_17	1	0	1	0	0	0
50	HSV-1_UL36/VZV_22/mCMV_M48/EBV_BPLF1/KSHV_64	HSV-1_UL26.5/VZV_33.5/mCMV_M80.5/EBV_BdRF1/KSHV_17.5	1	0	1	0	0	0
51	HSV-1_UL36/VZV_22/mCMV_M48/EBV_BPLF1/KSHV_64	HSV-1_UL18/VZV_41/mCMV_M85/EBV_BDLF1/KSHV_26	1	0	1	0	0	0
52	HSV-1_UL36/VZV_22/mCMV_M48/EBV_BPLF1/KSHV_64	HSV-1_UL17/VZV_43/mCMV_M93/EBV_BGLF1/KSHV_32	1	0	1	0	0	0
53	HSV-1_UL36/VZV_22/mCMV_M48/EBV_BPLF1/KSHV_64	HSV-1_UL2/VZV_59/mCMV_M114/EBV_BKRF3/KSHV_46	1	0	1	0	0	0
54	HSV-1_UL35/VZV_23/mCMV_M48.2/EBV_BFRF3/KSHV_65	HSV-1_UL19/VZV_40/mCMV_M86/EBV_BcLF1/KSHV_25	2	0	0	1	0	1
55	HSV-1_UL35/VZV_23/mCMV_M48.2/EBV_BFRF3/KSHV_65	HSV-1_UL33/VZV_25/mCMV_M51/EBV_BFRF4/KSHV_67.5	2	0	0	1	1	0
56	HSV-1_UL35/VZV_23/mCMV_M48.2/EBV_BFRF3/KSHV_65	HSV-1_UL35/VZV_23/mCMV_M48.2/EBV_BFRF3/KSHV_65	1	0	0	1	0	0
57	HSV-1_UL35/VZV_23/mCMV_M48.2/EBV_BFRF3/KSHV_65	HSV-1_UL10/VZV_50/mCMV_M100/EBV_BBRF3/KSHV_39	1	0	0	1	0	0
58	HSV-1_UL35/VZV_23/mCMV_M48.2/EBV_BFRF3/KSHV_65	HSV-1_UL49A/VZV_9a/mCMV_M73/EBV_BLRF1/KSHV_53	1	0	1	0	0	0
59	HSV-1_UL35/VZV_23/mCMV_M48.2/EBV_BFRF3/KSHV_65	HSV-1_UL31/VZV_27/mCMV_M53/EBV_BFLF2/KSHV_69	1	0	1	0	0	0
60	HSV-1_UL35/VZV_23/mCMV_M48.2/EBV_BFRF3/KSHV_65	HSV-1_UL1/VZV_60/mCMV_M115/EBV_BKRF2/KSHV_47	1	0	1	0	0	0
61	HSV-1_UL34/VZV_24/mCMV_M50/EBV_BFRF1/KSHV_67	HSV-1_UL10/VZV_50/mCMV_M100/EBV_BBRF3/KSHV_39	1	0	0	0	1	0
62	HSV-1_UL34/VZV_24/mCMV_M50/EBV_BFRF1/KSHV_67	HSV-1_UL31/VZV_27/mCMV_M53/EBV_BFLF2/KSHV_69	4	1	1	1	1	0
63	HSV-1_UL34/VZV_24/mCMV_M50/EBV_BFRF1/KSHV_67	HSV-1_UL34/VZV_24/mCMV_M50/EBV_BFRF1/KSHV_67	1	0	0	0	1	0
64	HSV-1_UL34/VZV_24/mCMV_M50/EBV_BFRF1/KSHV_67	HSV-1_UL33/VZV_25/mCMV_M51/EBV_BFRF4/KSHV_67.5	2	0	1	0	1	0
65	HSV-1_UL34/VZV_24/mCMV_M50/EBV_BFRF1/KSHV_67	HSV-1_UL17/VZV_43/mCMV_M93/EBV_BGLF1/KSHV_32	2	0	0	1	1	0
66	HSV-1_UL34/VZV_24/mCMV_M50/EBV_BFRF1/KSHV_67	HSV-1_UL1/VZV_60/mCMV_M115/EBV_BKRF2/KSHV_47	2	0	1	0	1	0
67	HSV-1_UL34/VZV_24/mCMV_M50/EBV_BFRF1/KSHV_67	HSV-1_UL18/VZV_41/mCMV_M85/EBV_BDLF1/KSHV_26	1	0	0	1	0	0
68	HSV-1_UL34/VZV_24/mCMV_M50/EBV_BFRF1/KSHV_67	HSV-1_UL27/VZV_31/mCMV_M55/EBV_BALF4/KSHV_8	1	0	0	1	0	0
69	HSV-1_UL34/VZV_24/mCMV_M50/EBV_BFRF1/KSHV_67	HSV-1_UL14/VZV_46/mCMV_M95/EBV_BGLF3/KSHV_34	2	1	1	0	0	0
70	HSV-1_UL34/VZV_24/mCMV_M50/EBV_BFRF1/KSHV_67	HSV-1_UL8/VZV_52/mCMV_M102/EBV_BBLF2/EBV_BBLF3/KSHV_40/KSHV_41	1	0	1	0	0	0
71	HSV-1_UL33/VZV_25/mCMV_M51/EBV_BFRF4/KSHV_67.5	HSV-1_UL30/VZV_28/mCMV_M54/EBV_BALF5/KSHV_9	4	1	0	1	1	1
72	HSV-1_UL33/VZV_25/mCMV_M51/EBV_BFRF4/KSHV_67.5	HSV-1_UL21/VZV_38/mCMV_M88/EBV_BTRF1/KSHV_23	3	0	1	0	1	1
73	HSV-1_UL33/VZV_25/mCMV_M51/EBV_BFRF4/KSHV_67.5	HSV-1_UL14/VZV_46/mCMV_M95/EBV_BGLF3/KSHV_34	2	0	0	1	0	1

	Protein 1	Protein2	Virus Occurences	HSV-1	VZV	mCMV	EBV	KSHV
74	HSV-1_UL33/VZV_25/mCMV_M51/EBV_BFRF4/KSHV_67.5	HSV-1_UL31/VZV_27/mCMV_M53/EBV_BFLF2/KSHV_69	4	0	1	1	1	1
75	HSV-1_UL33/VZV_25/mCMV_M51/EBV_BFRF4/KSHV_67.5	HSV-1_UL15/VZV_42/VZV_45/mCMV_M89/EBV_BGRF1/EBV_BDRF1/KSHV_29a/KSHV_29b	3	0	1	0	1	1
76	HSV-1_UL33/VZV_25/mCMV_M51/EBV_BFRF4/KSHV_67.5	HSV-1_UL29/VZV_29/mCMV_M57/EBV_BALF2/KSHV_6	1	0	0	0	1	0
77	HSV-1_UL33/VZV_25/mCMV_M51/EBV_BFRF4/KSHV_67.5	HSV-1_UL27/VZV_31/mCMV_M55/EBV_BALF4/KSHV_8	1	0	0	0	1	0
78	HSV-1_UL33/VZV_25/mCMV_M51/EBV_BFRF4/KSHV_67.5	HSV-1_UL8/VZV_52/mCMV_M102/EBV_BBLF2/EBV_BBLF3/KSHV_40/KSHV_41	2	0	1	0	2	0
79	HSV-1_UL33/VZV_25/mCMV_M51/EBV_BFRF4/KSHV_67.5	HSV-1_UL5/VZV_55/mCMV_M105/EBV_BBLF4/KSHV_44	2	0	1	0	1	0
80	HSV-1_UL33/VZV_25/mCMV_M51/EBV_BFRF4/KSHV_67.5	HSV-1_UL26.5/VZV_33.5/mCMV_M80.5/EBV_BdRF1/KSHV_17.5	2	0	1	0	1	0
81	HSV-1_UL33/VZV_25/mCMV_M51/EBV_BFRF4/KSHV_67.5	HSV-1_UL28/VZV_30/mCMV_M56/EBV_BALF3/KSHV_7	3	1	1	0	1	0
82	HSV-1_UL33/VZV_25/mCMV_M51/EBV_BFRF4/KSHV_67.5	HSV-1_UL10/VZV_50/mCMV_M100/EBV_BBRF3/KSHV_39	3	0	1	1	1	0
83	HSV-1_UL33/VZV_25/mCMV_M51/EBV_BFRF4/KSHV_67.5	HSV-1_UL33/VZV_25/mCMV_M51/EBV_BFRF4/KSHV_67.5	4	1	1	1	1	0
84	HSV-1_UL33/VZV_25/mCMV_M51/EBV_BFRF4/KSHV_67.5	HSV-1_UL17/VZV_43/mCMV_M93/EBV_BGLF1/KSHV_32	4	1	1	1	1	0
85	HSV-1_UL33/VZV_25/mCMV_M51/EBV_BFRF4/KSHV_67.5	HSV-1_UL1/VZV_60/mCMV_M115/EBV_BKRF2/KSHV_47	1	0	0	0	1	0
86	HSV-1_UL33/VZV_25/mCMV_M51/EBV_BFRF4/KSHV_67.5	HSV-1_UL51/VZV_7/mCMV_M71/EBV_BSRF1/KSHV_55	1	0	0	0	1	0
87	HSV-1_UL33/VZV_25/mCMV_M51/EBV_BFRF4/KSHV_67.5	HSV-1_UL25/VZV_34/mCMV_M77/EBV_BVRF1/KSHV_19	1	0	0	1	0	0
88	HSV-1_UL33/VZV_25/mCMV_M51/EBV_BFRF4/KSHV_67.5	HSV-1_UL50/VZV_8/mCMV_M72/EBV_BLLF3/KSHV_54	2	0	1	1	0	0
89	HSV-1_UL33/VZV_25/mCMV_M51/EBV_BFRF4/KSHV_67.5	HSV-1_UL13/VZV_47/mCMV_M97/EBV_BGLF4/KSHV_36	1	0	0	1	0	0
90	HSV-1_UL33/VZV_25/mCMV_M51/EBV_BFRF4/KSHV_67.5	HSV-1_UL7/VZV_53/mCMV_M103/EBV_BBRF2/KSHV_42	2	1	0	1	0	0
91	HSV-1_UL33/VZV_25/mCMV_M51/EBV_BFRF4/KSHV_67.5	HSV-1_UL16/VZV_44/mCMV_M94/EBV_BGLF2/KSHV_33	2	1	1	0	0	0
92	HSV-1_UL33/VZV_25/mCMV_M51/EBV_BFRF4/KSHV_67.5	HSV-1_UL2/VZV_59/mCMV_M114/EBV_BKRF3/KSHV_46	2	1	1	0	0	0
93	HSV-1_UL33/VZV_25/mCMV_M51/EBV_BFRF4/KSHV_67.5	HSV-1_UL49A/VZV_9a/mCMV_M73/EBV_BLRF1/KSHV_53	2	1	1	0	0	0
94	HSV-1_UL33/VZV_25/mCMV_M51/EBV_BFRF4/KSHV_67.5	HSV-1_UL26/VZV_33/mCMV_M80/EBV_BVRF2/KSHV_17	1	0	1	0	0	0
95	HSV-1_UL33/VZV_25/mCMV_M51/EBV_BFRF4/KSHV_67.5	HSV-1_UL18/VZV_41/mCMV_M85/EBV_BDLF1/KSHV_26	1	0	1	0	0	0
96	HSV-1_UL33/VZV_25/mCMV_M51/EBV_BFRF4/KSHV_67.5	HSV-1_UL11/VZV_49/mCMV_M99/EBV_BBLF1/KSHV_38	1	0	1	0	0	0
97	HSV-1_UL32/VZV_26/mCMV_M52/EBV_BFLF1/KSHV_68	HSV-1_UL30/VZV_28/mCMV_M54/EBV_BALF5/KSHV_9	1	0	0	0	0	1
98	HSV-1_UL32/VZV_26/mCMV_M52/EBV_BFLF1/KSHV_68	HSV-1_UL54/VZV_4/mCMV_M69/EBV_BSLF2/EBV_BMLF1/KSHV_57	1	0	0	0	0	1
99	HSV-1_UL32/VZV_26/mCMV_M52/EBV_BFLF1/KSHV_68	HSV-1_UL15/VZV_42/VZV_45/mCMV_M89/EBV_BGRF1/EBV_BDRF1/KSHV_29a/KSHV_29b	2	0	0	0	1	1
100	HSV-1_UL32/VZV_26/mCMV_M52/EBV_BFLF1/KSHV_68	HSV-1_UL32/VZV_26/mCMV_M52/EBV_BFLF1/KSHV_68	2	0	1	0	1	0
101	HSV-1_UL32/VZV_26/mCMV_M52/EBV_BFLF1/KSHV_68	HSV-1_UL31/VZV_27/mCMV_M53/EBV_BFLF2/KSHV_69	1	0	0	0	1	0
102	HSV-1_UL32/VZV_26/mCMV_M52/EBV_BFLF1/KSHV_68	HSV-1_UL16/VZV_44/mCMV_M94/EBV_BGLF2/KSHV_33	1	0	0	0	1	0
103	HSV-1_UL32/VZV_26/mCMV_M52/EBV_BFLF1/KSHV_68	HSV-1_UL13/VZV_47/mCMV_M97/EBV_BGLF4/KSHV_36	1	0	0	1	0	0
104	HSV-1_UL32/VZV_26/mCMV_M52/EBV_BFLF1/KSHV_68	HSV-1_UL22/VZV_37/mCMV_M75/EBV_BXLF2/KSHV_22	1	0	0	1	0	0
105	HSV-1_UL32/VZV_26/mCMV_M52/EBV_BFLF1/KSHV_68	HSV-1_UL21/VZV_38/mCMV_M88/EBV_BTRF1/KSHV_23	1	0	1	0	0	0
106	HSV-1_UL32/VZV_26/mCMV_M52/EBV_BFLF1/KSHV_68	HSV-1_UL14/VZV_46/mCMV_M95/EBV_BGLF3/KSHV_34	1	0	1	0	0	0
107	HSV-1_UL31/VZV_27/mCMV_M53/EBV_BFLF2/KSHV_69	HSV-1_UL27/VZV_31/mCMV_M55/EBV_BALF4/KSHV_8	1	0	0	0	1	0
108	HSV-1_UL31/VZV_27/mCMV_M53/EBV_BFLF2/KSHV_69	HSV-1_UL8/VZV_52/mCMV_M102/EBV_BBLF2/EBV_BBLF3/KSHV_40/KSHV_41	1	0	0	0	1	0
109	HSV-1_UL31/VZV_27/mCMV_M53/EBV_BFLF2/KSHV_69	HSV-1_UL28/VZV_30/mCMV_M56/EBV_BALF3/KSHV_7	1	0	0	0	1	0
110	HSV-1_UL31/VZV_27/mCMV_M53/EBV_BFLF2/KSHV_69	HSV-1_UL15/VZV_42/VZV_45/mCMV_M89/EBV_BGRF1/EBV_BDRF1/KSHV_29a/KSHV_29b	2	0	1	0	1	0

	Protein 1	Protein2	Virus Occurences	HSV-1	VZV	mCMV	EBV	KSHV
111	HSV-1_UL31/VZV_27/mCMV_M53/EBV_BFLF2/KSHV_69	HSV-1_UL16/VZV_44/mCMV_M94/EBV_BGLF2/KSHV_33	1	0	0	0	1	0
112	HSV-1_UL31/VZV_27/mCMV_M53/EBV_BFLF2/KSHV_69	HSV-1_UL51/VZV_7/mCMV_M71/EBV_BSRF1/KSHV_55	1	0	0	0	1	0
113	HSV-1_UL31/VZV_27/mCMV_M53/EBV_BFLF2/KSHV_69	HSV-1_UL7/VZV_53/mCMV_M103/EBV_BBRF2/KSHV_42	1	0	0	1	0	0
114	HSV-1_UL31/VZV_27/mCMV_M53/EBV_BFLF2/KSHV_69	HSV-1_UL49A/VZV_9a/mCMV_M73/EBV_BLRF1/KSHV_53	1	0	1	0	0	0
115	HSV-1_UL31/VZV_27/mCMV_M53/EBV_BFLF2/KSHV_69	HSV-1_UL31/VZV_27/mCMV_M53/EBV_BFLF2/KSHV_69	1	0	1	0	0	0
116	HSV-1_UL31/VZV_27/mCMV_M53/EBV_BFLF2/KSHV_69	HSV-1_UL26/VZV_33/mCMV_M80/EBV_BVRF2/KSHV_17	1	0	1	0	0	0
117	HSV-1_UL31/VZV_27/mCMV_M53/EBV_BFLF2/KSHV_69	HSV-1_UL26.5/VZV_33.5/mCMV_M80.5/EBV_BdRF1/KSHV_17.5	1	0	1	0	0	0
118	HSV-1_UL31/VZV_27/mCMV_M53/EBV_BFLF2/KSHV_69	HSV-1_UL25/VZV_34/mCMV_M77/EBV_BVRF1/KSHV_19	1	0	1	0	0	0
119	HSV-1_UL31/VZV_27/mCMV_M53/EBV_BFLF2/KSHV_69	HSV-1_UL21/VZV_38/mCMV_M88/EBV_BTRF1/KSHV_23	1	0	1	0	0	0
120	HSV-1_UL31/VZV_27/mCMV_M53/EBV_BFLF2/KSHV_69	HSV-1_UL17/VZV_43/mCMV_M93/EBV_BGLF1/KSHV_32	1	0	1	0	0	0
121	HSV-1_UL31/VZV_27/mCMV_M53/EBV_BFLF2/KSHV_69	HSV-1_UL10/VZV_50/mCMV_M100/EBV_BBRF3/KSHV_39	1	0	1	0	0	0
122	HSV-1_UL31/VZV_27/mCMV_M53/EBV_BFLF2/KSHV_69	HSV-1_UL1/VZV_60/mCMV_M115/EBV_BKRF2/KSHV_47	1	0	1	0	0	0
123	HSV-1_UL30/VZV_28/mCMV_M54/EBV_BALF5/KSHV_9	HSV-1_UL10/VZV_50/mCMV_M100/EBV_BBRF3/KSHV_39	1	0	0	0	0	1
124	HSV-1_UL30/VZV_28/mCMV_M54/EBV_BALF5/KSHV_9	HSV-1_UL8/VZV_52/mCMV_M102/EBV_BBLF2/EBV_BBLF3/KSHV_40/KSHV_41	1	0	0	0	0	1
125	HSV-1_UL30/VZV_28/mCMV_M54/EBV_BALF5/KSHV_9	HSV-1_UL1/VZV_60/mCMV_M115/EBV_BKRF2/KSHV_47	2	0	1	0	0	1
126	HSV-1_UL30/VZV_28/mCMV_M54/EBV_BALF5/KSHV_9	HSV-1_UL28/VZV_30/mCMV_M56/EBV_BALF3/KSHV_7	1	0	0	0	1	0
127	HSV-1_UL30/VZV_28/mCMV_M54/EBV_BALF5/KSHV_9	HSV-1_UL16/VZV_44/mCMV_M94/EBV_BGLF2/KSHV_33	1	0	0	0	1	0
128	HSV-1_UL30/VZV_28/mCMV_M54/EBV_BALF5/KSHV_9	HSV-1_UL13/VZV_47/mCMV_M97/EBV_BGLF4/KSHV_36	1	0	0	1	0	0
129	HSV-1_UL30/VZV_28/mCMV_M54/EBV_BALF5/KSHV_9	HSV-1_UL14/VZV_46/mCMV_M95/EBV_BGLF3/KSHV_34	1	1	0	0	0	0
130	HSV-1_UL27/VZV_31/mCMV_M55/EBV_BALF4/KSHV_8	HSV-1_UL28/VZV_30/mCMV_M56/EBV_BALF3/KSHV_7	1	0	0	0	1	0
131	HSV-1_UL27/VZV_31/mCMV_M55/EBV_BALF4/KSHV_8	HSV-1_UL8/VZV_52/mCMV_M102/EBV_BBLF2/EBV_BBLF3/KSHV_40/KSHV_41	1	0	0	0	1	0
132	HSV-1_UL27/VZV_31/mCMV_M55/EBV_BALF4/KSHV_8	HSV-1_UL10/VZV_50/mCMV_M100/EBV_BBRF3/KSHV_39	1	0	0	0	1	0
133	HSV-1_UL27/VZV_31/mCMV_M55/EBV_BALF4/KSHV_8	HSV-1_UL15/VZV_42/VZV_45/mCMV_M89/EBV_BGRF1/EBV_BDRF1/KSHV_29a/KSHV_29b	1	0	0	0	1	0
134	HSV-1_UL27/VZV_31/mCMV_M55/EBV_BALF4/KSHV_8	HSV-1_UL16/VZV_44/mCMV_M94/EBV_BGLF2/KSHV_33	1	0	0	0	1	0
135	HSV-1_UL27/VZV_31/mCMV_M55/EBV_BALF4/KSHV_8	HSV-1_UL14/VZV_46/mCMV_M95/EBV_BGLF3/KSHV_34	1	0	0	0	1	0
136	HSV-1_UL27/VZV_31/mCMV_M55/EBV_BALF4/KSHV_8	HSV-1_UL12/VZV_48/mCMV_M98/EBV_BGLF5/KSHV_37	1	0	0	0	1	0
137	HSV-1_UL27/VZV_31/mCMV_M55/EBV_BALF4/KSHV_8	HSV-1_UL2/VZV_59/mCMV_M114/EBV_BKRF3/KSHV_46	1	0	0	0	1	0
138	HSV-1_UL27/VZV_31/mCMV_M55/EBV_BALF4/KSHV_8	HSV-1_UL51/VZV_7/mCMV_M71/EBV_BSRF1/KSHV_55	1	0	0	0	1	0
139	HSV-1_UL27/VZV_31/mCMV_M55/EBV_BALF4/KSHV_8	HSV-1_UL21/VZV_38/mCMV_M88/EBV_BTRF1/KSHV_23	1	0	0	0	1	0
140	HSV-1_UL27/VZV_31/mCMV_M55/EBV_BALF4/KSHV_8	HSV-1_UL49A/VZV_9a/mCMV_M73/EBV_BLRF1/KSHV_53	1	0	0	1	0	0
141	HSV-1_UL28/VZV_30/mCMV_M56/EBV_BALF3/KSHV_7	HSV-1_UL29/VZV_29/mCMV_M57/EBV_BALF2/KSHV_6	1	0	0	0	1	0
142	HSV-1_UL28/VZV_30/mCMV_M56/EBV_BALF3/KSHV_7	HSV-1_UL12/VZV_48/mCMV_M98/EBV_BGLF5/KSHV_37	1	0	0	0	1	0
143	HSV-1_UL28/VZV_30/mCMV_M56/EBV_BALF3/KSHV_7	HSV-1_UL21/VZV_38/mCMV_M88/EBV_BTRF1/KSHV_23	1	0	0	0	1	0
144	HSV-1_UL28/VZV_30/mCMV_M56/EBV_BALF3/KSHV_7	HSV-1_UL8/VZV_52/mCMV_M102/EBV_BBLF2/EBV_BBLF3/KSHV_40/KSHV_41	1	0	0	0	2	0
145	HSV-1_UL28/VZV_30/mCMV_M56/EBV_BALF3/KSHV_7	HSV-1_UL5/VZV_55/mCMV_M105/EBV_BBLF4/KSHV_44	1	0	0	0	1	0
146	HSV-1_UL28/VZV_30/mCMV_M56/EBV_BALF3/KSHV_7	HSV-1_UL26.5/VZV_33.5/mCMV_M80.5/EBV_BdRF1/KSHV_17.5	1	0	0	0	1	0
147	HSV-1_UL28/VZV_30/mCMV_M56/EBV_BALF3/KSHV_7	HSV-1_UL15/VZV_42/VZV_45/mCMV_M89/EBV_BGRF1/EBV_BDRF1/KSHV_29a/KSHV_29b	1	0	0	0	1	0
148	HSV-1_UL54/VZV_4/mCMV_M69/EBV_BSLF2/EBV_BMLF1/KSHV_57	HSV-1_UL21/VZV_38/mCMV_M88/EBV_BTRF1/KSHV_23	2	0	1	0	0	1

	Protein 1	Protein2	Virus Occurences	HSV-1	VZV	mCMV	EBV	KSHV
149	HSV-1_UL54/VZV_4/mCMV_M69/EBV_BSLF2/EBV_BMLF1/KSHV_57	HSV-1_UL54/VZV_4/mCMV_M69/EBV_BSLF2/EBV_BMLF1/KSHV_57	3	1	0	1	0	1
150	HSV-1_UL54/VZV_4/mCMV_M69/EBV_BSLF2/EBV_BMLF1/KSHV_57	HSV-1_UL6/VZV_54/mCMV_M104/EBV_BBRF1/KSHV_43	1	0	0	0	1	0
151	HSV-1_UL54/VZV_4/mCMV_M69/EBV_BSLF2/EBV_BMLF1/KSHV_57	HSV-1_UL26.5/VZV_33.5/mCMV_M80.5/EBV_BdRF1/KSHV_17.5	1	0	0	0	1	0
152	HSV-1_UL54/VZV_4/mCMV_M69/EBV_BSLF2/EBV_BMLF1/KSHV_57	HSV-1_UL24/VZV_35/mCMV_M76/EBV_BXRF1/KSHV_20	1	0	0	0	1	0
153	HSV-1_UL54/VZV_4/mCMV_M69/EBV_BSLF2/EBV_BMLF1/KSHV_57	HSV-1_UL15/VZV_42/VZV_45/mCMV_M89/EBV_BGRF1/EBV_BDRF1/KSHV_29a/KSHV_29b	1	0	0	1	0	0
154	HSV-1_UL54/VZV_4/mCMV_M69/EBV_BSLF2/EBV_BMLF1/KSHV_57	HSV-1_UL50/VZV_8/mCMV_M72/EBV_BLLF3/KSHV_54	1	0	0	1	0	0
155	HSV-1_UL52/VZV_6/mCMV_M70/EBV_BSLF1/KSHV_56	HSV-1_UL13/VZV_47/mCMV_M97/EBV_BGLF4/KSHV_36	1	0	0	0	0	1
156	HSV-1_UL51/VZV_7/mCMV_M71/EBV_BSRF1/KSHV_55	HSV-1_UL7/VZV_53/mCMV_M103/EBV_BBRF2/KSHV_42	2	0	1	0	1	0
157	HSV-1_UL51/VZV_7/mCMV_M71/EBV_BSRF1/KSHV_55	HSV-1_UL15/VZV_42/VZV_45/mCMV_M89/EBV_BGRF1/EBV_BDRF1/KSHV_29a/KSHV_29b	1	0	0	0	1	0
158	HSV-1_UL51/VZV_7/mCMV_M71/EBV_BSRF1/KSHV_55	HSV-1_UL12/VZV_48/mCMV_M98/EBV_BGLF5/KSHV_37	1	0	0	0	1	0
159	HSV-1_UL51/VZV_7/mCMV_M71/EBV_BSRF1/KSHV_55	HSV-1_UL21/VZV_38/mCMV_M88/EBV_BTRF1/KSHV_23	1	0	0	0	1	0
160	HSV-1_UL51/VZV_7/mCMV_M71/EBV_BSRF1/KSHV_55	HSV-1_UL51/VZV_7/mCMV_M71/EBV_BSRF1/KSHV_55	1	0	1	0	0	0
161	HSV-1_UL50/VZV_8/mCMV_M72/EBV_BLLF3/KSHV_54	HSV-1_UL13/VZV_47/mCMV_M97/EBV_BGLF4/KSHV_36	1	0	0	0	0	1
162	HSV-1_UL50/VZV_8/mCMV_M72/EBV_BLLF3/KSHV_54	HSV-1_UL15/VZV_42/VZV_45/mCMV_M89/EBV_BGRF1/EBV_BDRF1/KSHV_29a/KSHV_29b	1	0	0	0	0	1
163	HSV-1_UL50/VZV_8/mCMV_M72/EBV_BLLF3/KSHV_54	HSV-1_UL11/VZV_49/mCMV_M99/EBV_BBLF1/KSHV_38	1	0	0	1	0	0
164	HSV-1_UL50/VZV_8/mCMV_M72/EBV_BLLF3/KSHV_54	HSV-1_UL17/VZV_43/mCMV_M93/EBV_BGLF1/KSHV_32	1	0	0	1	0	0
165	HSV-1_UL50/VZV_8/mCMV_M72/EBV_BLLF3/KSHV_54	HSV-1_UL10/VZV_50/mCMV_M100/EBV_BBRF3/KSHV_39	1	0	0	1	0	0
166	HSV-1_UL49A/VZV_9a/mCMV_M73/EBV_BLRF1/KSHV_53	HSV-1_UL10/VZV_50/mCMV_M100/EBV_BBRF3/KSHV_39	1	1	0	0	0	0
167	HSV-1_UL49A/VZV_9a/mCMV_M73/EBV_BLRF1/KSHV_53	HSV-1_UL49A/VZV_9a/mCMV_M73/EBV_BLRF1/KSHV_53	1	1	0	0	0	0
168	HSV-1_UL49A/VZV_9a/mCMV_M73/EBV_BLRF1/KSHV_53	HSV-1_UL15/VZV_42/VZV_45/mCMV_M89/EBV_BGRF1/EBV_BDRF1/KSHV_29a/KSHV_29b	1	1	0	0	0	0
169	HSV-1_UL49A/VZV_9a/mCMV_M73/EBV_BLRF1/KSHV_53	HSV-1_UL1/VZV_60/mCMV_M115/EBV_BKRF2/KSHV_47	1	0	1	0	0	0
170	HSV-1_UL25/VZV_34/mCMV_M77/EBV_BVRF1/KSHV_19	HSV-1_UL17/VZV_43/mCMV_M93/EBV_BGLF1/KSHV_32	1	0	0	1	0	0
171	HSV-1_UL25/VZV_34/mCMV_M77/EBV_BVRF1/KSHV_19	HSV-1_UL18/VZV_41/mCMV_M85/EBV_BDLF1/KSHV_26	1	0	0	1	0	0
172	HSV-1_UL25/VZV_34/mCMV_M77/EBV_BVRF1/KSHV_19	HSV-1_UL21/VZV_38/mCMV_M88/EBV_BTRF1/KSHV_23	1	0	0	1	0	0
173	HSV-1_UL25/VZV_34/mCMV_M77/EBV_BVRF1/KSHV_19	HSV-1_UL7/VZV_53/mCMV_M103/EBV_BBRF2/KSHV_42	1	0	0	1	0	0
174	HSV-1_UL25/VZV_34/mCMV_M77/EBV_BVRF1/KSHV_19	HSV-1_UL25/VZV_34/mCMV_M77/EBV_BVRF1/KSHV_19	1	0	1	0	0	0
175	HSV-1_UL25/VZV_34/mCMV_M77/EBV_BVRF1/KSHV_19	HSV-1_UL15/VZV_42/VZV_45/mCMV_M89/EBV_BGRF1/EBV_BDRF1/KSHV_29a/KSHV_29b	1	0	1	0	0	0
176	HSV-1_UL26/VZV_33/mCMV_M80/EBV_BVRF2/KSHV_17	HSV-1_UL26/VZV_33/mCMV_M80/EBV_BVRF2/KSHV_17	2	0	1	1	0	0
177	HSV-1_UL26/VZV_33/mCMV_M80/EBV_BVRF2/KSHV_17	HSV-1_UL26.5/VZV_33.5/mCMV_M80.5/EBV_BdRF1/KSHV_17.5	1	0	1	0	0	0
178	HSV-1_UL26/VZV_33/mCMV_M80/EBV_BVRF2/KSHV_17	HSV-1_UL1/VZV_60/mCMV_M115/EBV_BKRF2/KSHV_47	1	0	1	0	0	0
179	HSV-1_UL26.5/VZV_33.5/mCMV_M80.5/EBV_BdRF1/KSHV_17.5	HSV-1_UL8/VZV_52/mCMV_M102/EBV_BBLF2/EBV_BBLF3/KSHV_40/KSHV_41	1	0	0	0	1	0
180	HSV-1_UL26.5/VZV_33.5/mCMV_M80.5/EBV_BdRF1/KSHV_17.5	HSV-1_UL10/VZV_50/mCMV_M100/EBV_BBRF3/KSHV_39	1	0	0	0	1	0
181	HSV-1_UL26.5/VZV_33.5/mCMV_M80.5/EBV_BdRF1/KSHV_17.5	HSV-1_UL15/VZV_42/VZV_45/mCMV_M89/EBV_BGRF1/EBV_BDRF1/KSHV_29a/KSHV_29b	1	0	0	0	1	0
182	HSV-1_UL26.5/VZV_33.5/mCMV_M80.5/EBV_BdRF1/KSHV_17.5	HSV-1_UL16/VZV_44/mCMV_M94/EBV_BGLF2/KSHV_33	1	0	0	0	1	0
183	HSV-1_UL26.5/VZV_33.5/mCMV_M80.5/EBV_BdRF1/KSHV_17.5	HSV-1_UL14/VZV_46/mCMV_M95/EBV_BGLF3/KSHV_34	1	0	0	0	1	0

	Protein 1	Protein2	Virus Occurences	HSV-1	VZV	mCMV	EBV	KSHV
184	HSV-1_UL26.5/VZV_33.5/mCMV_M80.5/EBV_BdRF1/KSHV_17.5	HSV-1_UL12/VZV_48/mCMV_M98/EBV_BGLF5/KSHV_37	1	0	0	0	1	0
185	HSV-1_UL26.5/VZV_33.5/mCMV_M80.5/EBV_BdRF1/KSHV_17.5	HSV-1_UL21/VZV_38/mCMV_M88/EBV_BTRF1/KSHV_23	1	0	0	0	1	0
186	HSV-1_UL26.5/VZV_33.5/mCMV_M80.5/EBV_BdRF1/KSHV_17.5	HSV-1_UL1/VZV_60/mCMV_M115/EBV_BKRF2/KSHV_47	1	0	1	0	0	0
187	HSV-1_UL18/VZV_41/mCMV_M85/EBV_BDLF1/KSHV_26	HSV-1_UL17/VZV_43/mCMV_M93/EBV_BGLF1/KSHV_32	1	0	0	1	0	0
188	HSV-1_UL18/VZV_41/mCMV_M85/EBV_BDLF1/KSHV_26	HSV-1_UL18/VZV_41/mCMV_M85/EBV_BDLF1/KSHV_26	1	0	0	1	0	0
189	HSV-1_UL18/VZV_41/mCMV_M85/EBV_BDLF1/KSHV_26	HSV-1_UL1/VZV_60/mCMV_M115/EBV_BKRF2/KSHV_47	1	0	1	0	0	0
190	HSV-1_UL21/VZV_38/mCMV_M88/EBV_BTRF1/KSHV_23	HSV-1_UL15/VZV_42/VZV_45/mCMV_M89/EBV_BGRF1/EBV_BDRF1/KSHV_29a/KSHV_29b	2	0	0	0	1	1
191	HSV-1_UL21/VZV_38/mCMV_M88/EBV_BTRF1/KSHV_23	HSV-1_UL11/VZV_49/mCMV_M99/EBV_BBLF1/KSHV_38	1	0	0	0	1	0
192	HSV-1_UL21/VZV_38/mCMV_M88/EBV_BTRF1/KSHV_23	HSV-1_UL7/VZV_53/mCMV_M103/EBV_BBRF2/KSHV_42	1	0	0	0	1	0
193	HSV-1_UL21/VZV_38/mCMV_M88/EBV_BTRF1/KSHV_23	HSV-1_UL17/VZV_43/mCMV_M93/EBV_BGLF1/KSHV_32	2	0	0	1	1	0
194	HSV-1_UL21/VZV_38/mCMV_M88/EBV_BTRF1/KSHV_23	HSV-1_UL16/VZV_44/mCMV_M94/EBV_BGLF2/KSHV_33	2	1	1	0	0	0
195	HSV-1_UL21/VZV_38/mCMV_M88/EBV_BTRF1/KSHV_23	HSV-1_UL14/VZV_46/mCMV_M95/EBV_BGLF3/KSHV_34	1	0	1	0	0	0
196	HSV-1_UL15/VZV_42/VZV_45/mCMV_M89/EBV_BGRF1/EBV_BDRF1/KSHV_29a/KSHV_29b	HSV-1_UL8/VZV_52/mCMV_M102/EBV_BBLF2/EBV_BBLF3/KSHV_40/KSHV_41	2	0	0	0	1	1
197	HSV-1_UL15/VZV_42/VZV_45/mCMV_M89/EBV_BGRF1/EBV_BDRF1/KSHV_29a/KSHV_29b	HSV-1_UL15/VZV_42/VZV_45/mCMV_M89/EBV_BGRF1/EBV_BDRF1/KSHV_29a/KSHV_29b	1	0	0	0	1	0
198	HSV-1_UL15/VZV_42/VZV_45/mCMV_M89/EBV_BGRF1/EBV_BDRF1/KSHV_29a/KSHV_29b	HSV-1_UL16/VZV_44/mCMV_M94/EBV_BGLF2/KSHV_33	1	0	0	0	1	0
199	HSV-1_UL15/VZV_42/VZV_45/mCMV_M89/EBV_BGRF1/EBV_BDRF1/KSHV_29a/KSHV_29b	HSV-1_UL12/VZV_48/mCMV_M98/EBV_BGLF5/KSHV_37	1	0	0	0	1	0
200	HSV-1_UL15/VZV_42/VZV_45/mCMV_M89/EBV_BGRF1/EBV_BDRF1/KSHV_29a/KSHV_29b	HSV-1_UL17/VZV_43/mCMV_M93/EBV_BGLF1/KSHV_32	2	0	0	1	1	0
201	HSV-1_UL15/VZV_42/VZV_45/mCMV_M89/EBV_BGRF1/EBV_BDRF1/KSHV_29a/KSHV_29b	HSV-1_UL7/VZV_53/mCMV_M103/EBV_BBRF2/KSHV_42	2	1	0	1	0	0
202	HSV-1_UL15/VZV_42/VZV_45/mCMV_M89/EBV_BGRF1/EBV_BDRF1/KSHV_29a/KSHV_29b	HSV-1_UL14/VZV_46/mCMV_M95/EBV_BGLF3/KSHV_34	1	0	1	0	0	0
203	HSV-1_UL15/VZV_42/VZV_45/mCMV_M89/EBV_BGRF1/EBV_BDRF1/KSHV_29a/KSHV_29b	HSV-1_UL1/VZV_60/mCMV_M115/EBV_BKRF2/KSHV_47	1	0	1	0	0	0
204	HSV-1_UL17/VZV_43/mCMV_M93/EBV_BGLF1/KSHV_32	HSV-1_UL17/VZV_43/mCMV_M93/EBV_BGLF1/KSHV_32	1	0	0	1	0	0
205	HSV-1_UL17/VZV_43/mCMV_M93/EBV_BGLF1/KSHV_32	HSV-1_UL1/VZV_60/mCMV_M115/EBV_BKRF2/KSHV_47	1	0	1	0	0	0
206	HSV-1_UL16/VZV_44/mCMV_M94/EBV_BGLF2/KSHV_33	HSV-1_UL11/VZV_49/mCMV_M99/EBV_BBLF1/KSHV_38	3	1	0	1	1	0
207	HSV-1_UL16/VZV_44/mCMV_M94/EBV_BGLF2/KSHV_33	HSV-1_UL14/VZV_46/mCMV_M95/EBV_BGLF3/KSHV_34	1	1	0	0	0	0
208	HSV-1_UL16/VZV_44/mCMV_M94/EBV_BGLF2/KSHV_33	HSV-1_UL7/VZV_53/mCMV_M103/EBV_BBRF2/KSHV_42	1	1	0	0	0	0

	Protein 1	Protein2	Virus Occurences	HSV-1	VZV	mCMV	EBV	KSHV
209	HSV-1_UL14/VZV_46/mCMV_M95/EBV_BGLF3/KSHV_34	HSV-1_UL13/VZV_47/mCMV_M97/EBV_BGLF4/KSHV_36	1	0	0	1	0	0
210	HSV-1_UL14/VZV_46/mCMV_M95/EBV_BGLF3/KSHV_34	HSV-1_UL14/VZV_46/mCMV_M95/EBV_BGLF3/KSHV_34	1	1	0	0	0	0
211	HSV-1_UL14/VZV_46/mCMV_M95/EBV_BGLF3/KSHV_34	HSV-1_UL2/VZV_59/mCMV_M114/EBV_BKRF3/KSHV_46	1	1	0	0	0	0
212	HSV-1_UL14/VZV_46/mCMV_M95/EBV_BGLF3/KSHV_34	HSV-1_UL7/VZV_53/mCMV_M103/EBV_BBRF2/KSHV_42	1	1	0	0	0	0
213	HSV-1_UL13/VZV_47/mCMV_M97/EBV_BGLF4/KSHV_36	HSV-1_UL12/VZV_48/mCMV_M98/EBV_BGLF5/KSHV_37	1	0	0	1	0	0
214	HSV-1_UL13/VZV_47/mCMV_M97/EBV_BGLF4/KSHV_36	HSV-1_UL7/VZV_53/mCMV_M103/EBV_BBRF2/KSHV_42	1	0	0	1	0	0
215	HSV-1_UL12/VZV_48/mCMV_M98/EBV_BGLF5/KSHV_37	HSV-1_UL2/VZV_59/mCMV_M114/EBV_BKRF3/KSHV_46	2	1	0	1	0	0
216	HSV-1_UL10/VZV_50/mCMV_M100/EBV_BBRF3/KSHV_39	HSV-1_UL10/VZV_50/mCMV_M100/EBV_BBRF3/KSHV_39	1	0	1	0	0	0
217	HSV-1_UL7/VZV_53/mCMV_M103/EBV_BBRF2/KSHV_42	HSV-1_UL7/VZV_53/mCMV_M103/EBV_BBRF2/KSHV_42	1	1	0	0	0	0
218	HSV-1_UL1/VZV_60/mCMV_M115/EBV_BKRF2/KSHV_47	HSV-1_UL1/VZV_60/mCMV_M115/EBV_BKRF2/KSHV_47	1	0	1	0	0	0
