# Supplementary material for: Evolutionarily Conserved Herpesviral Protein Interaction Networks
Source: PLoS Pathog. 2009 Sep 4;5(9):e1000570. doi: 10.1371/journal.ppat.1000570 (PMC2731838; doi:10.1371/journal.ppat.1000570)
Supplement: Table S8 — Average degree values for core and non-core proteins in all viruses. Average degree of core vs non-core proteins for all five interactomes. P-values were calculated with a Wilcoxon rank test between the degree values of core and non-core proteins. (0.06 MB PDF) [file ppat.1000570.s022.pdf]

Table S8: Average degree values for *core* and *non-core* proteins in all viruses.

Average Degree	HSV-1	VZV	mCMV	EBV	KSHV
**Core**	2.46	5.75	5.37	6.51	2.85
**Non-Core**	3.67	3.33	4.29	3.13	2.75
**P-value**	0.101	0.197	0.128	0.004	0.88
