# Supplementary material for: Evolutionarily Conserved Herpesviral Protein Interaction Networks
Source: PLoS Pathog. 2009 Sep 4;5(9):e1000570. doi: 10.1371/journal.ppat.1000570 (PMC2731838; doi:10.1371/journal.ppat.1000570)
Supplement: Table S9 — Ortholog protein interactions (predicted from KSHV) tested by Y2H and CoIP. List of orthologous interactions predicted from the KSHV interactome [23] which were tested by co-immunoprecipitation in HSV-1, mCMV and EBV. Results from the Y2H analysis of the predicted interactions are also indicated. (0.00 MB PDF) [file ppat.1000570.s023.pdf]

Table S9: Ortholog protein interactions (predicted from KSHV) tested by Y2H and CoIP.

HSV-1					mCMV					EBV					KSHV [23]			
		Y2H	CoIP				Y2H	CoIP				Y2H	CoIP				Y2H	CoIP
UL13	UL39	0	1		M97	M45	0	0		BGLF4	BORF2	0	0		Orf36	Orf61	1	1
										BMRF1	BFLF1	0	0		Orf59	Orf68	1	1
										BDLF3	BDLF3	0	0		Orf28	Orf28	1	0
										BGLF4	BKRF4	0	0		Orf36	Orf45	1	0
										BKRF4	BRLF1	0	1		Orf45	Orf50	1	0
										BRRF1	BLRF2	0	1		Orf49	Orf52	1	0
										BMRF1	BFRF4	0	0		Orf59	Orf67.5	1	0
										BFLF2	BLRF2	0	0		Orf69	Orf52	1	0
										BcLF1	BFRF3	0	1		Orf25	Orf65	1	1
										BDRF1	BDLF3	0	1		Orf29b	Orf28	1	1
										BLRF2	BLRF2	1	1		Orf52	Orf52	1	1
										BLLF3	BORF1	0	1		Orf54	Orf62	1	1
UL52	UL13	0	0		M70	M97	0	0		BSLF1	BGLF4	0	1		Orf56	Orf36	1	1
UL40	UL52	0	0							BaRF1	BSLF1	0	1		Orf60	Orf56	1	1
UL40	UL33	1	1							BaRF1	BFRF4	0	1		Orf60	Orf67.5	1	1
UL40	UL32	0	0							BaRF1	BFLF1	0	1		Orf60	Orf68	1	1
										BOLF1	BTRF1	0	1		Orf63	Orf23	1	1
										BOLF1	BFRF3	0	1		Orf63	Orf65	1	1
										BOLF1	BFRF4	0	1		Orf63	Orf67.5	1	1
										BNRF1	BRLF1	0	0		Orf75	Orf50	1	1
										BNRF1	BFRF4	1	0		Orf75	Orf67.5	1	0
										BALF2	BLRF2	0	1		Orf6	Orf52	1	0
										BALF5	BKRF2	0	0		Orf9	Orf47	1	0
										BTRF1	BDLF3	0	0		Orf23	Orf28	1	0
										BTRF1	BKRF4	0	0		Orf23	Orf45	1	0

HSV-1					mCMV					EBV					KSHV [23]			
		**Y2H**	**CoIP**				**Y2H**	**CoIP**				**Y2H**	**CoIP**				**Y2H**	**CoIP**
										BDLF3	BFRF4	0	0		Orf28	Orf67.5	1	0
										BDRF1	BRLF1	0	0		Orf29b	Orf50	1	0
										BGLF3	BLRF2	0	1		Orf34	Orf52	1	0
										BMLF1	BRLF1	0	1		Orf57	Orf50	1	0
										BMLF1	BLRF2	0	1		Orf57	Orf52	1	0
										BMRF2	BDLF2	1	0		Orf58	Orf27	1	0
										BMRF1	BLRF2	0	1		Orf59	Orf52	1	0
										BaRF1	BTRF1	0	1		Orf60	Orf23	1	0
										BaRF1	BLRF2	0	1		Orf60	Orf52	1	0
										BTRF1	BFRF4	1	1		Orf23	Orf67.5	1	1
										BDRF1	BTRF1	1	1		Orf29b	Orf23	1	1
										BDLF4	BDLF4	0	1		Orf31	Orf31	1	1
					M92	M92	0	1		BDLF4	BFRF4	0	1		Orf31	Orf67.5	1	1
					M92	M51	0	1		BDLF4	BFLF1	0	1		Orf31	Orf68	1	1
					M92	M52	0	1		BMLF1	BTRF1	0	1		Orf57	Orf23	1	1
UL15	UL50	0	0		M89	M72	0	1		BDRF1	BLLF3	0	1		Orf29b	Orf54	1	0
					M95	M51	1	1		BGLF3	BFRF4	0	1		Orf34	Orf67.5	1	0
UL40	UL40	1	1							BaRF1	BaRF1	0	1		Orf60	Orf60	1	1
UL40	UL39	0	0							BaRF1	BORF2	0	1		Orf60	Orf61	1	1
UL30	UL33	1	0		M54	M51	1	0		BALF5	BFRF4	1	1		Orf9	Orf67.5	1	1
UL15	UL33	0	1		M89	M51	0	1		BDRF1	BFRF4	1	1		Orf29b	Orf67.5	1	1
UL54	UL54	1	1		M69	M69	1	1		BMLF1	BMLF1	0	1		Orf57	Orf57	1	1
UL54	UL39	0	1		M69	M45	0	1		BMLF1	BORF2	0	1		Orf57	Orf61	1	1
UL54	UL32	0	1		M69	M52	0	0		BMLF1	BFLF1	0	0		Orf57	Orf68	1	1
UL31	UL33	0	1		M53	M51	1	1		BFLF2	BFRF4	1	1		Orf69	Orf67.5	1	1
UL30	UL10	0	0		M54	M100	0	0		BALF5	BBRF3	0	0		Orf9	Orf39	1	0
UL30	UL32	0	1		M54	M52	0	0		BALF5	BFLF1	0	0		Orf9	Orf68	1	0
UL15	UL32	0	1		M89	M52	0	1		BDRF1	BFLF1	1	0		Orf29b	Orf68	1	0

HSV-1					mCMV					EBV					KSHV [23]			
		**Y2H**	**CoIP**				**Y2H**	**CoIP**				**Y2H**	**CoIP**				**Y2H**	**CoIP**
UL13	UL50	0	0		M97	M72	0	1		BGLF4	BLLF3	0	1		Orf36	Orf54	1	0
UL39	UL39	0	1		M45	M45	1	1		BORF2	BORF2	0	0		Orf61	Orf61	1	1
