# Supplementary material for: Evolutionarily Conserved Herpesviral Protein Interaction Networks
Source: PLoS Pathog. 2009 Sep 4;5(9):e1000570. doi: 10.1371/journal.ppat.1000570 (PMC2731838; doi:10.1371/journal.ppat.1000570)
Supplement: Table S10 — Negatively predicted orthologous protein interactions (predicted from core interaction network) tested by Y2H and CoIP. Ten interactions were predicted to be negative, based on the fact that they were not observed in any of the five viral interactomes, and analysed by co-immunoprecipitation. (0.02 MB PDF) [file ppat.1000570.s024.pdf]

Table S10: Negatively predicted orthologous protein interactions (predicted from core interaction network) tested by Y2H and CoIP.

HSV-1					mCMV					EBV					KSHV			
		Y2H	CoIP				Y2H	CoIP				Y2H	CoIP				Y2H	CoIP
UL21	UL21	0	0		M88	M88	0	0		BTRF1	BTRF1	0	0		Orf 23	Orf 23	0	1
UL39	UL25	0	0		M71	M86	0	0		BSRF1	BcLF1	0	0		Orf 55	Orf 25	0	0
UL51	UL19	0	NA		M45	M77	0	0		BORF2	BVRF1	0	0		Orf 19	Orf 61	0	0
UL12	UL6	0	1		M98	M104	0	0		BGLF5	BBRF1	0	1		Orf 37	Orf 43	0	0
UL13	UL5	0	0		M97	M105	0	0		BGLF4	BBLF4	0	0		Orf 36	Orf 44	0	0
UL10	UL19	0	0		M100	M86	0	0		BBRF3	BcLF1	0	0		ORF39	ORF26	0	0
UL5	UL22	0	1		M105	M75	0	0		BBLF4	BXLF2	0	0		ORF44	ORF22	0	1
UL24	UL52	0	0		M70	M76	0	1		BSLF1	BXRF1	0	0		ORF56	ORF20	0	0
UL54	UL30	0	0		M69	M54	0	0		BSLF2	BALF5	0	0		ORF57	ORF9	0	0
UL24	UL18	0	0		M76	M85	0	0		BXRF1	BDLF1	0	0		ORF20	ORF26	0	0
