# Supplementary material for: Evolutionarily Conserved Herpesviral Protein Interaction Networks
Source: PLoS Pathog. 2009 Sep 4;5(9):e1000570. doi: 10.1371/journal.ppat.1000570 (PMC2731838; doi:10.1371/journal.ppat.1000570)
Supplement: Table S11 — Analysis of interspecies interactions. VZV core and noncore baits were analysed for Y2H interactions against prey libraries of VZV, HSV-1, mCMV, EBV and KSHV. The species of the interacting prey is included, in addition to whether it was a core or a noncore protein. (0.02 MB PDF) [file ppat.1000570.s025.pdf]

Table S11: Analysis of interspecies interactions

Bait ID, VZV	Bait: CORE/ NON-CORE	Prey	Prey ID	Prey: CORE/ NON-CORE
ORF 19	CORE	VZV	ORF 16	CORE
ORF 19	CORE	VZV	ORF 18	NON-CORE
ORF 19	CORE	VZV	ORF 19	CORE
ORF 19	CORE	VZV	ORF 25	CORE
ORF 19	CORE	VZV	ORF 38	CORE
ORF 23	CORE	VZV	ORF 25	CORE
ORF 23	CORE	VZV	ORF 60	CORE
ORF 24	CORE	VZV	ORF 27	CORE
ORF 25	CORE	VZV	ORF 25	CORE
ORF 25	CORE	VZV	ORF 38	CORE
ORF 25	CORE	VZV	ORF 62	NON-CORE
ORF 17	NON-CORE	VZV	ORF 42	CORE
ORF 36	NON-CORE	VZV	ORF 16	CORE
ORF 36	NON-CORE	VZV	ORF 25	CORE
ORF 36	NON-CORE	VZV	ORF 36	NON-CORE
ORF 51	NON-CORE	VZV	ORF 25	CORE
ORF 19	CORE	HSV-1	UL 40	NON-CORE
ORF 19	CORE	mCMV	M32	NON-CORE
ORF 36	NON-CORE	mCMV	M10	NON-CORE
ORF 19	CORE	mCMV	M51	CORE
ORF 19	CORE	mCMV	M73	CORE
ORF 19	CORE	mCMV	M107	NON-CORE
ORF 23	CORE	mCMV	M87	NON-CORE
ORF 25	CORE	mCMV	M119.3	NON-CORE
ORF 19	CORE	mCMV	M134	NON-CORE
ORF 19	CORE	mCMV	M141	NON-CORE
ORF 19	CORE	mCMV	M168	NON-CORE
ORF 25	CORE	mCMV	M163	NON-CORE
ORF 19	CORE	EBV	BFRF4	CORE
ORF 19	CORE	EBV	BDLF2	NON-CORE
ORF 24	CORE	EBV	BCRF2	NON-CORE
ORF 19	CORE	KSHV	ORF 45	NON-CORE
ORF 19	CORE	KSHV	K10	NON-CORE
ORF 19	CORE	KSHV	ORF 62	CORE
ORF 51	NON-CORE	KSHV	K3	NON-CORE
ORF 24	CORE	KSHV	ORF 74	NON-CORE
ORF 24	CORE	KSHV	K3	NON-CORE
