# Supplementary material for: Evolutionarily Conserved Herpesviral Protein Interaction Networks
Source: PLoS Pathog. 2009 Sep 4;5(9):e1000570. doi: 10.1371/journal.ppat.1000570 (PMC2731838; doi:10.1371/journal.ppat.1000570)
Supplement: Table S12 — M51 interactions tested by CoIP. Interactions observed with mCMV M51, or with other orthologs of M51, with a subset of interaction partners from the Y2H analysis. Tegument proteins, and other virion components, were determined based on whether they were reported to be present in the CMV virion [59],[60]. (0.01 MB PDF) [file ppat.1000570.s026.pdf]

Table S12: M51 interactions tested by CoIP.

		Function	Positive CoIP
M51	M45	Tegument	Yes
M51	M46	Capsid	No
M51	M48	Tegument	No
M51	M50	glycoprotein	Yes
M51	M53	Non-structural	Yes
M51	M54	Tegument	Yes
M51	M55	gB	No
M51	M56	Tegument	Yes
M51	M69	Tegument	No
M51	M70	Tegument	Yes
M51	M72	Tegument	Yes
M51	M77	Tegument	Yes
M51	M80	Scaffold protein	Yes
M51	M85	Capsid	Yes
M51	M89	Tegument	Yes
M51	M93	Tegument	Yes
M51	M94	Tegument	Yes
M51	M97	Tegument	Yes
M51	M100	gM	No
M51	M103	Tegument	Yes
M51	M105	Tegument	Yes
M51	M114	Non-structural	Yes

.
