# Supplementary material for: Evolutionarily Conserved Herpesviral Protein Interaction Networks
Source: PLoS Pathog. 2009 Sep 4;5(9):e1000570. doi: 10.1371/journal.ppat.1000570 (PMC2731838; doi:10.1371/journal.ppat.1000570)
Supplement: Figure S3 — Network of reported protein interactions in HSV-1, VZV, mCMV, KSHV and EBV. Network structure of protein interactions reported previously for the five herpesvirus species which were identified by text mining and drawn with the Cytoscape software [58]. The width of the edges indicates for how many of the five species the interaction was reported. Interactions observed in our Y2H analysis are indicated with red edges. (0.24 MB PDF) [file ppat.1000570.s004.pdf]

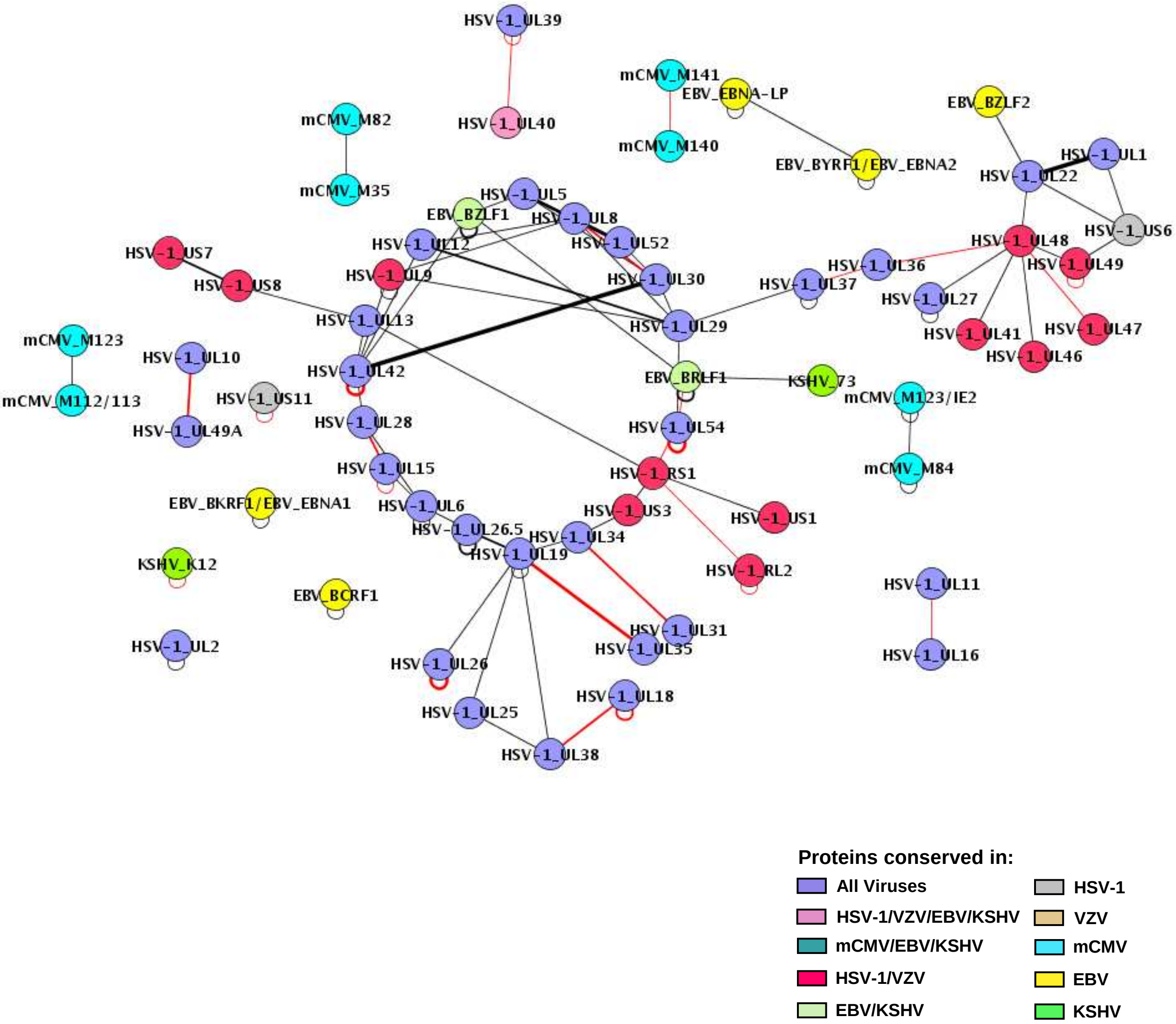
HSV-1_UL39
HSV-1_UL40
mCMV_M141
EBV_EBNA-LP
mCMV_M140
EBV_BZLF2
mCMV_M82
mCMV_M35
EBV_BYRF1/EBV_EBNA2
HSV-1_UL1
HSV-1_UL22
HSV-1_UL5
EBV_BZLF1
HSV-1_UL8
HSV-1_UL52
HSV-1_UL12
HSV-1_US6
HSV-1_US7
HSV-1_UL48
HSV-1_UL36
HSV-1_UL49
HSV-1_UL9
HSV-1_UL30
HSV-1_US8
HSV-1_UL37
HSV-1_UL27
HSV-1_UL13
HSV-1_UL29
HSV-1_UL41
HSV-1_UL47
mCMV_M123
HSV-1_UL10
HSV-1_UL46
HSV-1_UL42
EBV_BRLF1
KSHV_73
HSV-1_US11
mCMV_M112/113
mCMV_M123/IE2
HSV-1_UL49A
HSV-1_UL28
HSV-1_UL54
mCMV_M84
HSV-1_UL15
HSV-1_RS1
EBV_BKRF1/EBV_EBNA1
HSV-1_UL6
HSV-1_US3
HSV-1_US1
HSV-1_UL26.5
HSV-1_UL34
HSV-1_UL19
KSHV_K12
HSV-1_RL2
EBV_BCRF1
HSV-1_UL11
HSV-1_UL31
HSV-1_UL35
HSV-1_UL2
HSV-1_UL16
HSV-1_UL26
HSV-1_UL18
HSV-1_UL25
HSV-1_UL38
Proteins conserved in:
All Viruses
HSV-1/VZV/EBV/KSHV
mCMV/EBV/KSHV
HSV-1/VZV
EBV/KSHV
HSV-1
VZV
mCMV
EBV
KSHV
